# Supplementary material for: Ultraviolet light blocking optically clear adhesives for foldable displays via highly efficient visible-light curing
Source: Nat Commun. 2024 Apr 2;15:2829. doi: 10.1038/s41467-024-47104-y (PMC10987679; doi:10.1038/s41467-024-47104-y)
Supplement: Supplementary file 1 — Supplementary Information [file 41467_2024_47104_MOESM1_ESM.pdf]

Supplementary Information

Ultraviolet light blocking optically clear adhesives for foldable displays via highly efficient visible-light curing

Yonghwan Kwon[1,†], Seokju Lee[1,†], Junkyu Kim[1], Jinwon Jun[2], Woojin Jeon[1], Youngjoo Park[1], Hyun-Joong Kim[3], Johannes Gierschner[4], Jaesang Lee[2,*], Youngdo Kim[5,*], and Min Sang Kwon[1,*]

[1]Department of Materials Science and Engineering, Seoul National University, Seoul, Republic of Korea

[2]Department of Electrical and Computer Engineering, Seoul National University, Seoul, Republic of Korea

[3]Department of Agriculture, Forestry and Bioresources, Seoul National University, Seoul, Republic of Korea

[4]Madrid Institute for Advanced Studies, IMDEA Nanoscience, Calle Faraday 9, Campus Cantoblanco, 28049 Madrid, Spain

[5]Samsung Display Co., Ltd., Cheonan, Republic of Korea

[†]These authors contributed equally: Yonghwan Kwon (Y.Kwon), Seokju Lee (S.L.)

e-mail: minsang@snu.ac.kr (M.S.K.), colour.kim@samsung.com (Y.Kim), jsanglee@snu.ac.kr (J.L.)

Contents

Supplementary Note 1. General information 3

1.1. Chemicals 3

1.2. General experimental procedures 3

1.3. Instrumentation 4

1.4. Computational methods 9

Supplementary Note 2. Characterization of PC, co-initiator and UVA 10

2.1. Syntheses of PCs and UVA-1 10

2.2. Characterization of PC, co-initiator and UVA 13

2.3. Computational characterization of PIS: kinetic simulation 24

Supplementary Note 3. Visible-light driven PIS for synthesis of UV-blocking OCA 32

3.1. Characterization of OCA film 32

3.2. Synthesis of UV-blocking OCA 33

Supplementary Note 4. Characterization of UV-blocking OCA 40

4.1. Optical properties of UV-blocking OCA 40

4.2. Mechanical properties of UV-blocking OCA 41

Supplementary Note 5. Coordinates of molecular structures obtained by DFT calculation 48

Supplementary References 71

Supplementary Note 1. General information

1.1. Chemicals

4DP-IPN, 4Cz-IPN and dimethyl 2-(4-(dimethylamino)benzylidene)malonate) (UVA-1) were synthesized according to the procedures previously reported by our group.[1–3] All chemicals and solvents for the syntheses were commercially purchased and used without further purifications. Ethyl 2-cyano-3,3-diphenylacrylate (UVA-2, TCI), 2-ethylhexyl acrylate (EHA, Aldrich), 4-hydroxybutyl acrylate (HBA, TCI), butyl acrylate (BA, Aldrich), 2-dimethylaminoethyl acrylate (DMAEA, Aldrich), 2-dimethylaminoethyl acetate (DMAEAc, TCI), [4-(Octyloxy)phenyl](phenyl)iodonium hexafluoroantimonate (HNu 254, TCI) and 2-butanoyloxyethyl(trimethyl)azanium butyl(triphenyl)boranuide (Borate V, Spectra Group Limited, Inc.) were purchased commercially. Acrylic monomers were purified by passing from basic alumina (Aldrich) to remove the inhibitors. Polyethylene terephthalate (PETE, 50 μm, youngwoo trading), release film (silicon-treated PETE film, 100 μm, youngwoo trading), super release film (silicon-treated PETE film, 100 μm, youngwoo trading) and colorless polyimide (CPI, Kolon Industries, 50 μm) were commercially purchased.

1.2. General experimental procedures

■ General experimental procedures for synthesis of acrylic syrup

A 20 ml vial (glass, Sungho SIGMA) equipped with a stirring bar was charged with monomer mixture, PCs, and co-initiators. Afterwards, the vial was capped with a rubber septum and sealed with parafilm and degassed with 99.999% N_2 for 30 min. PCs and co-initiators were used after dilution in acrylate monomers for the reproducibility. In the case of addition of UV absorbers (UVAs), they were added in this reaction solution before a bulk polymerization in the newly designed PIS. During the degassing process, the reaction solution was kept in dark condition to block the room light preventing undesired polymerization. Subsequently, acrylic syrup was synthesized under irradiation of 3W MR 16 light-emitting diode (LED) (λ_{max} = 455 nm, 25–100 mW cm^{-2}) at RT.

■ General experimental procedures for film curing

For film curing, two 15 W string-type blue LEDs (λ_{max} = 452 nm) were used, and their intensities were set up as 10 mW cm^{-2}. Film curing was carried out by additionally irradiating visible light to the prepared acrylic syrup. Since the PIS used in this process reuses the PC within the catalyst cycle of the acrylic syrup preparation process, no new additives were added during the film process. Moreover, a crosslinking agent was also not included in this process. For film curing, acrylic syrup was coated between two release films and casted to a thickness of 50 μm using a film casting applicator. The samples for measurements of optical and viscoelastic properties were cured for 40 min for the previous PIS (i.e., 4Cz-IPN and tertiary amines) and 30 min for the newly designed PIS (i.e., 4DP-IPN, HNu 254 and Borate V) to obtain fully cured OCAs.

1.3. Instrumentation

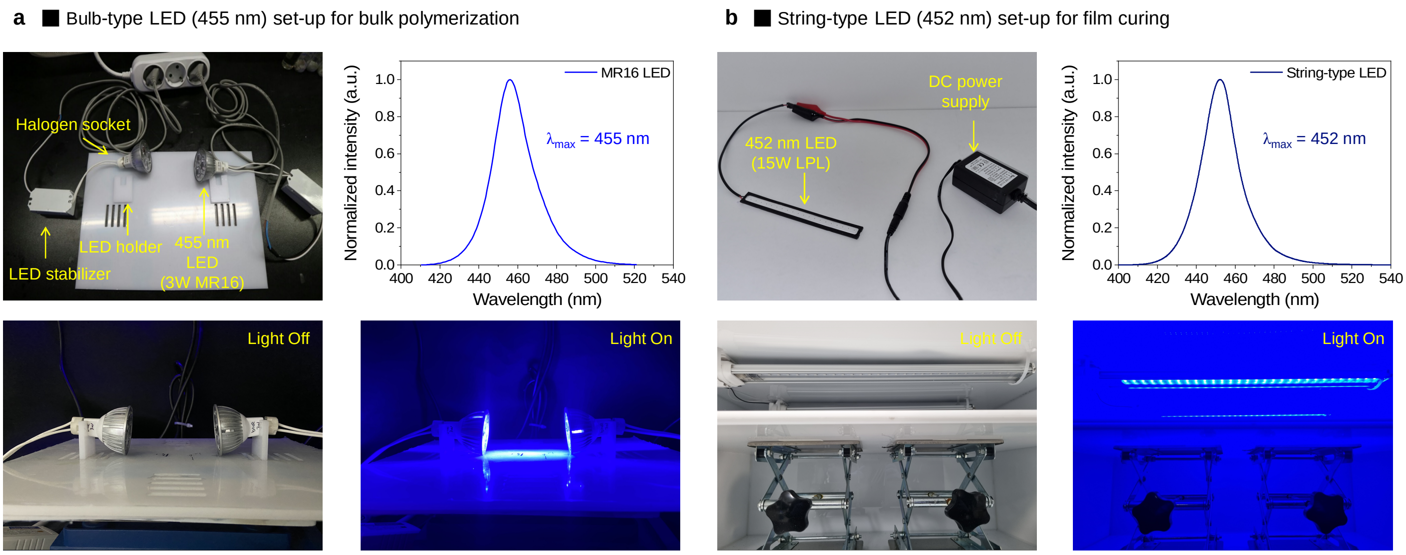

Supplementary Fig. 1 Experimental set-up for bulk polymerization and film curing. Blue LEDs set-up for **a** bulk polymerization for the synthesis of acrylic syrup and **b** film curing. LED set-up emission spectra were also given.

■ Photophysical measurements

UV/vis spectrophotometer at RT were measured with a V-770 (JASCO) UV-Vis-NIR spectrometer equipped with a halogen lamp and photomultiplier tube (PMT) for a correction in UV/vis region. Steady-state PL emission spectra at RT were obtained with QuantaMaster 40 UV/vis steady state spectrofluorometer (Photon Technology International Inc.) equipped with a 75W Xe short arc lamp; the emission spectra were corrected for the sensitivity of the PMT. Low-temperature steady-state PL spectra was measured with a FP-8300 (JASCO) spectrofluorometer and their gated low-temperature gated PL spectra were acquired with a delay of 100 ms. PL decay measurements were carried out by the time-correlated single photon counting (TCSPC) technique on a FluoTime 200 (PicoQuant) spectrometer equipped with a PMA182 PMT (PicoQuant) using a PicoHarp 300 TCSPC board (for prompt fluorescence) and NanoHarp 250 TCSPC (for delayed fluorescence). The excitation source was a 377 nm pulsed diode laser (FWHM ~ 70 ps) (LDH series PicoQuant). The decay time fitting procedure was carried out by using the Fluofit software (PicoQuant).

■ Electrochemical measurements

Cyclic voltammetry (CV) experiments were carried out with VSP-300 Potentiostat (Bio-Logic SAS) using a one compartment electrolysis cell consisting of a glassy carbon working electrode, a Pt wire counter electrode, and a quasi Ag^+/Ag (saturated KCl aqueous solution) reference electrode (AT FRONTIER, Part No. R303). Specifically, the reference electrode is an Ag wire coated with a thin layer of AgCl and consists of a porous plug on the one end, which allow the contact between the field environment with the AgCl electrolyte. Saturated KCl aqueous solution was added inside the body of the reference electrode to stabilize the concentration of AgCl, and in this condition the electrode's reference potential is known to be +0.197 V at 25 ℃. The measurements were done in 0.2 mM CH_3CN solution with 0.1 M n-Bu_4NPF_6 (Aldrich, Electrochemical grade) as supporting electrolyte at a scan rate of 100 mV s^{-1}. All redox potentials were calibrated after each measurement against Fc^+/Fc and converted to the aqueous saturated calomel electrode (SCE) scaled by using E^0 (Fc^+/Fc) = 0.42 V vs SCE in CH_3CN.[4] The sample solutions were degassed with 99.9999% argon for 15 min before the measurements and then kept under the positive argon pressure during the measurements.

■ Fourier-transform infrared (FT-IR) spectroscopy measurements

FT-IR spectroscopy was performed with Nicolet iS50 (Thermo Fisher Scientific) coupled with four position source mirror (involving polaris long-lifetime mid-IR source, tungsten-halogen NIR/Vis source, Raman InGaAs detector, focused emission port), three position detector mirror equipped with DLaTGS detector, and attenuated total reflectance (ATR, iS50, all-reflective diamond ATR, mid- to far-IR capable: 80 to over 5000 cm^{-1}, pressure applied to 60 lbs). Conversion of acrylic syrup and film was measured ATR mode, and scan number was set as n = 16. For the measurement of conversion of the prepared acrylic syrup and OCA film, the samples were kept under dark conditions to avoid undesired exposure to external light and measured without any post-treatment.

■ Gel permeation chromatography (GPC) measurements

Molecular weight was measured with GPC (Waters system; Waters 1515 isocratic pump, Waters 2707 autosampler) coupled with refractive index (RI) detector (Waters 2414 RI detector), UV/vis detector (Waters 2489 UV/vis detector), Gel permeation chromatography-multi-angle laser light scattering (GPC-MALS, Wyatt DAWN 8) and three different columns (Agilent Polypore 300 × 7.5 mm, Jordi mixed bed 300 × 8.0 mm, Waters Styragel HR4 300 × 7.8 mm).

■ Dynamic mechanical analysis (DMA) measurements

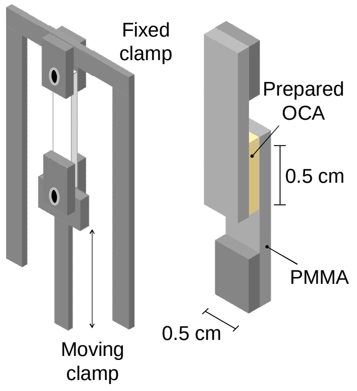

Supplementary Fig. 2 Experimental setup for DMA.

Viscoelastic properties including strain recovery, stress relaxation and maximum stress were evaluated with Q800 (TA instrument). Film tension clamp used for evaluation of soft materials such as rubber was employed.[5] Specimen for DMA was prepared by laminating the adhesive to a thickness of 50–100 μm and then cutting to a certain size (1 cm × 0.5 cm). After that, specimen was prepared by bonding between polymethylmethacrylate substrates. The measurements of strain recovery were carried out at 25 ℃ over 5 min, after the specimen was kept for 10 min at 300% strain,[6] with preload force set as 0.001 N.

■ **Peel strength measurements**

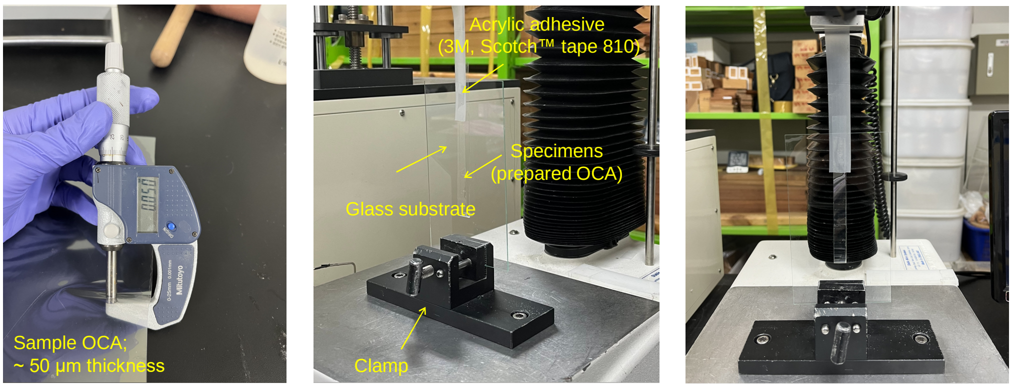

Supplementary Fig. 3 Experimental setup for peel strength measurement. Images of setup are also given.

The peel strength test was conducted with LS1 (AMETEK, USA) and followed the Korean Industrial Standards (KS T 1028). A load cell with a capacity of 10 kgf was used, and the test specimens were fixed by clamp used for a tensile test. For the peel test specimens, the acrylic syrup was cured between PETE film and release films, and the tested specimens were prepared as 1 cm wide. After removal of the release films from the prepared specimens, the OCA film was attached to the adherend using a 2 kg roller (2 round trips over the film). After 24 h of attachment time, the peel strength of tape-type adhesive was measured. Peel strength was obtained from the average of measured strength values from 20% to 80% of working range.

■ **Dynamic folding test measurements**

Folding durability was evaluated with Foldy-200 (FlexiGO, Republic of Korea) coupled with an inspection system capable of micro vision, macro vision, display inspection, and surface profiling. The samples were prepared by stacking colorless polyimide (CPI, Kolon Industries, 50 μm), prepared OCA (50 μm), yellow PI (GL140A, 35 μm), CEF 3602 (3M, 50 μm), and yellow PI (GL140A, 35 μm) in this order (see Supplementary Fig. 31). The samples were measured with a fixed radius of curvature of 1.5 mm along with folding cycles as 0.5 Hz by 5 x 10^4 times at -20 °C.

■ **Rheometer measurements**

Storage modulus (G'), loss modulus (G''), and damping factor (tan δ) were evaluated with Discovery HR-3 (TA instrument) coupled with optical encoder dual reader, 2nd generation magnetic thrust bearing (Patent US # 7,137, 290 & 7,017,393), advanced drag cup motor (Patent US # 6,798,099), normal force rebalance transducer, new true position sensor (TPS), and active temperature control (ATC, Patent US # 6,931,915). Specimen for rheometer was prepared by laminating the OCA to a thickness of 0.8–1 mm and then cutting to a specific size (8 mm in diameter.). The measurements were carried out at -50–90 °C, 1 Hz 0.5% shear.

■ Gel content measurements

The fraction of crosslinked polymers was evaluated by gel content measurement. Approximately 0.5 g of OCA sample was dissolved in 35 ml toluene at RT for 24 h, then linear polymers were completely dissolved, whereas the crosslinked polymers were swelled. The crosslinked polymers were isolated by filtration using a steel mesh (#200, ~74 μm). After the filtration, the sample was dried under reduced pressure at 80 °C for 24 h. Gel content was calculated by following equation.

$$\text{Gel content (\%)} = \frac{W_{\text{filtered OCA}}}{W_{\text{initial OCA}}} \times 100$$

where $W_{\text{filtered OCA}}$ and $W_{\text{initial OCA}}$ mean weight of the crosslinked polymers filtered over mesh and weight of OCA samples that initially dissolved in toluene.

■ Preparation of organic light-emitting diode (OLED)

We fabricated bottom-emitting, blue fluorescent OLEDs through a thermal evaporation process carried out under a base pressure of 3 x 10^{-7} Torr. The device structure is as follows: 70 nm indium tin oxide as an anode, 5 nm hole-injection layer (HIL), 25 nm hole transport layer (HTL), 60 nm emission layer (EML), 30 nm electron transport layer (ETL), 1 nm electron injection layer (EIL) and 100 nm aluminum as a cathode. All the organic compounds used to construct these blue OLEDs were sourced from SFC Co., Ltd. The OLEDs had dimensions of 2 mm x 2 mm, defined as the overlapping area between the cathode and anode. To minimize the undesirable effects of environmental defects, all devices were encapsulated within a N_2 atmosphere, with O_2 and moisture concentrations maintained below 1 ppm. The OCA film was directly applied on to one side of the glass substrate where devices were not present. The OCA film serves the dual purpose of extracting photons emitted by OLEDs and allowing external UV light to penetrate through it and illuminate the OLEDs.

■ UV-blocking test of OLEDs covered by OCAs

Both an OLED covered by non-UV-blocking OCA and the same device with UV-blocking OCA underwent simultaneous illumination using a UV LED source (Kessil, PR160L) with a peak wavelength of λ_{max} = 368 nm and an intensity of 70 mW cm^{-2} at RT. Both devices were consistently operated under a constant current density of J = 25–100 mA cm^{-2}, utilizing a multi-channel source measure unit (Agilent Technologies, U2722A). During the measurement of luminance levels over time with a 10 min interval, we momentarily deactivated the UV LED for 5 s to prevent the detection of photoluminescent signals originating from the OLEDs due to UV irradiation. Upon turning off the UV LED, a waiting period of 3 s was included, followed by a 2 s measurement of electroluminescence from the OLEDs. This measurement involved the averaging of 20 collected signals, each spaced at 0.1 s intervals, in order to minimize detection errors. Degradation of OLEDs' luminance over time was captured using a photodiode (Thorlabs, SM1PD1A) and subsequently recorded through a data logger (Keysight, DAQ970A). All stages of OLED testing were fully automated through an in-house LabVIEW virtual instruments.

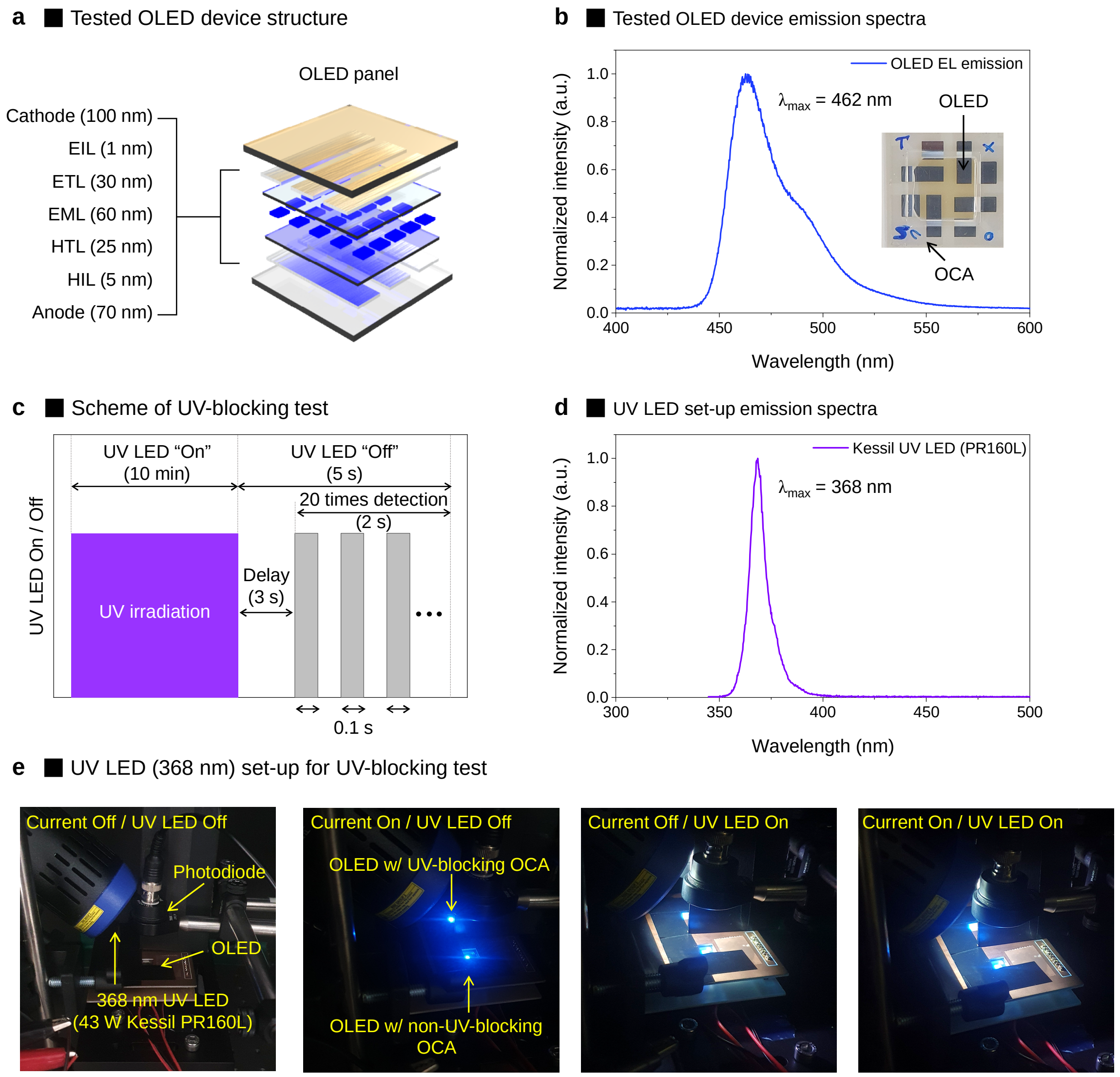

Supplementary Fig. 4 Experimental setup for UV-blocking test. **a** Structure of OLED devices tested in UV-blocking test. **b** Electroluminescence (EL) emission spectra of tested OLED devices. **c** Scheme of UV-blocking test of OCAs. **d** UV LED emission spectra used in UV-blocking test setup. **e** Images of experimental set-up for UV-blocking test.

1.4. Computational methods

■ Time-dependent density functional theory (TD-DFT) and kinetic simulation

Density functional theory (DFT) and time-dependent (TD)DFT calculations were performed with the B3LYP functional and 6–311++G* basis set as all implemented in the Gaussian16 program package. The geometries optimization, single point energies and oscillator strengths of vertical transition were calculated in ethyl acetate solution employing the polarizable continuum model (PCM). In all geometry optimization calculations, the frequency calculations were performed both to verify that the geometries were true minima and to obtain thermochemistry-correction values. To obtain the calculated excited state energy of PCs and UVAs, vertical transition energies for S_1 and T_1 were obtained by single-point calculations on the optimized S_0 geometries, respectively. The frontier molecular orbitals (MOs) were drawn by GaussView 6.0 software.

Kinetic simulation were performed with the ordinary differential equations (ODE) based on the rate law using ODE 15s or ODE 23s solvers implemented in the Matlab program package.[2,7] The concentration of S_1 and T_1 can be described by following system of ordinary differential equations (ODE).

$$\frac{d[\mathrm{S_1}]}{dt} = P_1 + k_{\mathrm{RISC}}[\mathrm{T_1}] - \left(k_{\mathrm{ISC}} + k_{\mathrm{r,S_1}} + k_{\mathrm{nr,S_1}}\right)[\mathrm{S_1}] \qquad (1)$$

$$\frac{d[\mathrm{T_1}]}{dt} = k_{\mathrm{ISC}}[\mathrm{S_1}] - (k_{\mathrm{RISC}} + k_{\mathrm{r,T_1}} + k_{\mathrm{nr,T_1}})[\mathrm{T_1}] \qquad (2)$$

where P_1 is a rate of S_1 generation from S_0 via photoexcitation (i.e., $S_0 \rightarrow S_n$) and fast internal conversion (i.e., $S_n \rightarrow S_1$), and k_{ISC}, k_{RISC}, $k_{\mathrm{r,S1}}$, $k_{\mathrm{r,T1}}$, $k_{\mathrm{nr,S1}}$ and $k_{\mathrm{nr,T1}}$ denote the rate constants of intersystem crossing, reverse-intersystem crossing, radiative decay from S_1, radiative decay from T_1, non-radiative decay from S_1, and non-radiative decay from T_1, respectively (see Supplementary Note 2.3 for the more detail).

Supplementary Note 2. Characterization of PC, co-initiator and UVA

2.1. Syntheses of PCs and UVA-1

■ Synthesis of 4DP-IPN

A solution of NaH (60% in mineral oil, 0.48 g, 11.94 mmol) and diphenylamine (1.48 g, 8.75 mmol) in anhydrous DMAc (5 ml) was stirred for 30 min in ice bath under N_2 atmosphere. After 30 min, 2,4,5,6-tetrafluoroisophthalonitrile (0.40 g, 1.99 mmol) dissolved in DMAc (5 ml) was slowly added to the reaction mixture and stirred further at 100 °C for 10 h. Afterwards, distilled water (2 ml) was poured into the reaction mixture to quench the excess NaH and, methanol was added to precipitate the crude product, which was further purified by column chromatography on silica gel (CH_2Cl_2:hexanes, 2:3 v/v) to give pure product as yellow powder (1.32 g, 83%). ^{1}H NMR data in full agreement with those reported in literature.[1,2] ^{1}H NMR (400 MHz, DMSO-d_6): δ 7.31–7.21 (m, 8H), 7.10–7.05 (t, 8H), 7.03–6.97 (t. 2H), 6.91–6.75 (m, 16H), 6.68–6.62 (m, 6H).

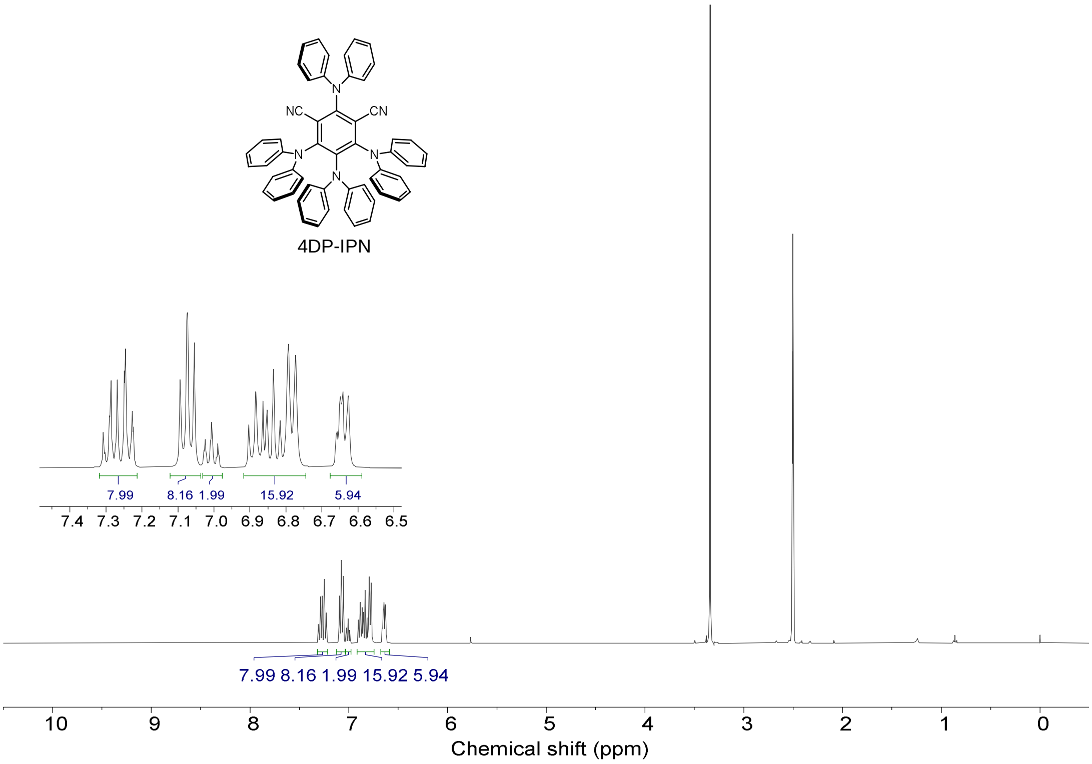

Supplementary Fig. 5 ^{1}H NMR data of 4DP-IPN at RT (400 MHz, DMSO-d_6). ^{1}H NMR (400 MHz, DMSO-d_6): δ 7.31–7.21 (m, 8H), 7.10–7.05 (t, 8H), 7.03–6.97 (t. 2H), 6.91–6.75 (m, 16H), 6.68–6.62 (m, 6H).

■ **Synthesis of 4Cz-IPN**

A solution of t-BuOK (0.19 g, 1.72 mmol) and 3,6-di-tert-butyl-9H-carbazole (0.40 g, 1.43 mmol) in anhydrous THF (40 ml) was stirred for 30 min in ice bath under N_2 atmosphere. After, 2,4,5,6-tetrafluoroisophthalonitrile (0.06 g, 0.28 mmol) was slowly added to the reaction mixture and stirred further for 12 h. After completion of the reaction, distilled water (2 ml) was poured into the reaction mixture to quench the excess t-BuOK. The resulting solution was concentrated under reduced pressure followed by washing several times with water and ethanol to yield the crude product, which was purified by column chromatography on silica gel (CH_2Cl_2:hexane, 2:1 v/v) to give pure product as pale yellow powder (0.16 g, 45%). ^{1}H NMR data in full agreement with those reported in literature.[1,2] ^{1}H NMR (400 MHz, $CDCl_3$): δ 8.21 (d, 2H), 7.74 (dd, 2H), 7.61–7.59 (m, 6H), 7.18 (d, 2H), 7.05–7.00 (m, 8H), 6.51 (dd, 2H), 6.44 (d, 2H), 1.53 (s, 18H), 1.30 (s, 36H), 1.22 (s, 18H).

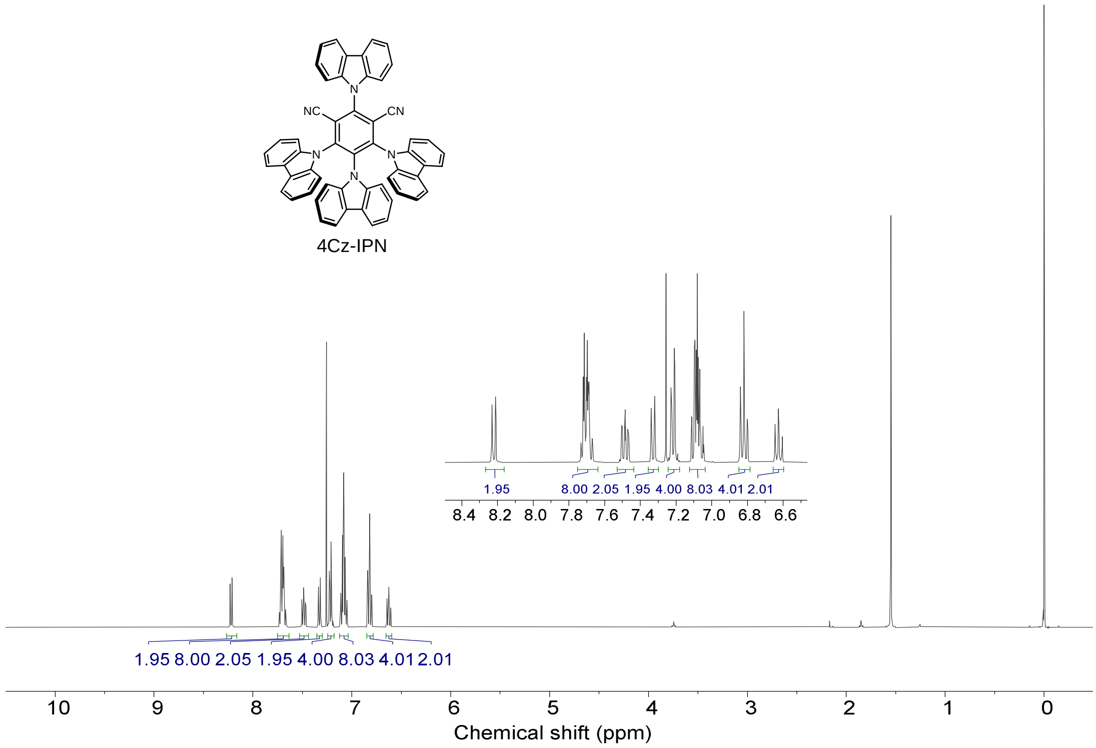

Supplementary Fig. 6 ^{1}H NMR data of 4Cz-IPN at RT (400 MHz, $CDCl_3$). ^{1}H NMR (400 MHz, $CDCl_3$): δ 8.22 (dt, 2H), 7.74–7.67 (m, 8H), 7.49 (ddd, 2H), 7.33 (dt, 2H), 7.23–7.21 (m, 4H), 7.12–7.05 (m, 8H), 6.82 (td, 4H), 6.63 (ddd, 2H).

■ **Synthesis of UVA-1**

4-(dimethylamino)benzaldehyde (5.00 g, 33.51 mmol) and dimethyl malonate (4.87 g, 36.86 mmol) were dissolved in methanol (50 ml). Then, DBU (1,8-diazabicyclo[5.4.0]undec-7-ene, 0.45 mL, 3.01 mmol) was added into the solution. The mixture was stirred at RT for 24 h. The solvent was partially removed under reduced pressure and the solution was poured into distilled water (100 ml). The generated precipitate was filtered and washed with water and methanol. After drying under reduced pressure, 7.54 g of dimethyl 2-(4-(dimethylamino)benzylidene)malonate) was obtained as yellow powder (yield: 85%). ^{1}H NMR data in full agreement with those reported in literature.[3] ^{1}H NMR (400 MHz, $CDCl_3$): δ 7.67 (s, 1H), 7.36–7.29 (m, 2H), 6.67–6.60 (m, 2H), 3.88 (d, 3H), 3.81 (d, 3H), 3.03 (d, 6H).

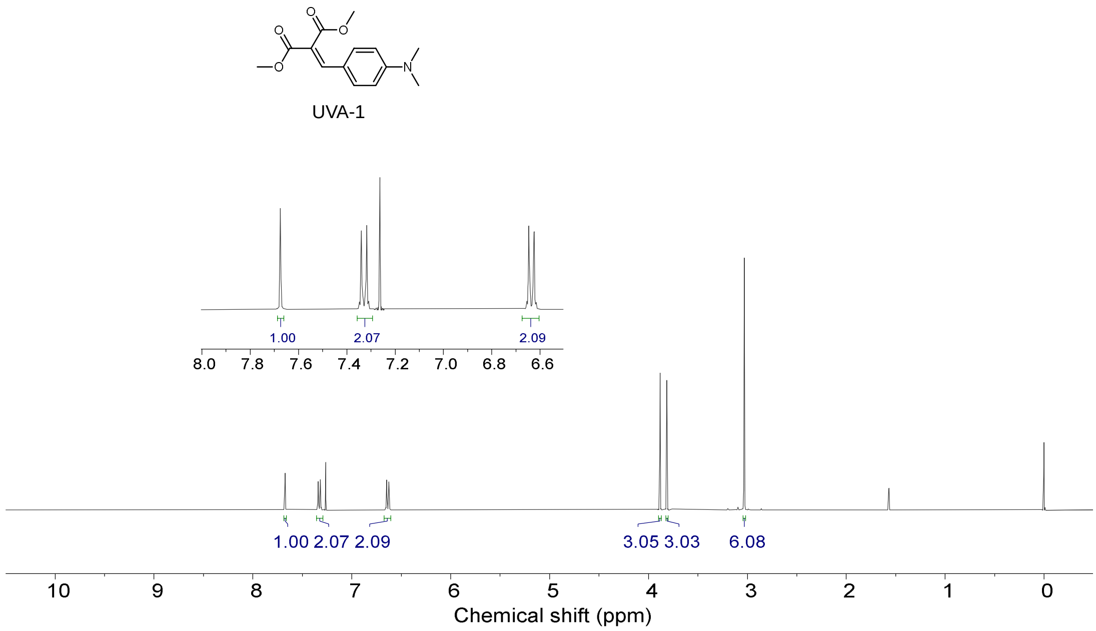

Supplementary Fig. 7 ^{1}H NMR data of UVA-1 at RT (400 MHz, $CDCl_3$). ^{1}H NMR (400 MHz, $CDCl_3$): δ 7.67 (s, 1H), 7.36–7.29 (m, 2H), 6.67–6.60 (m, 2H), 3.88 (d, 3H), 3.81 (d, 3H), 3.03 (d, 6H).

2.2. Characterization of PC, co-initiator and UVA

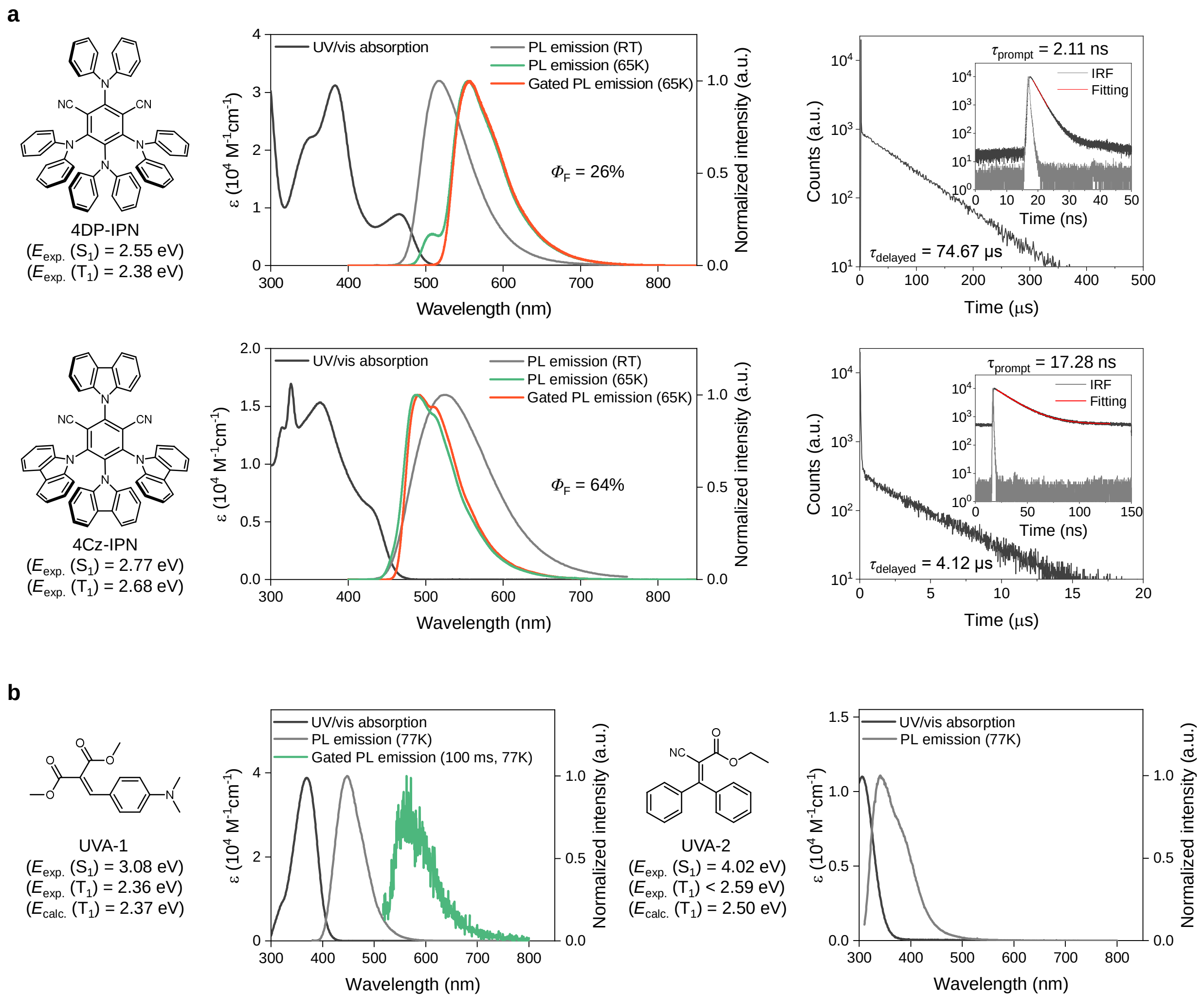

Supplementary Fig. 8 Chemical structures and photophysical properties of **a** PCs and **b** UVAs including UV/vis absorption and photoluminescence (PL) emission spectra in ethyl acetate acetate (1.0 × 10^{-5} M).[3] Delayed fluorescence decay spectra of PCs were obtained at RT after the degassing process by purging with 99.9999% argon for 10 min, however, prompt fluorescence decay were obtained without degassing process. E (S_1) and E (T_1) were extracted from the onset of PL and onset of gated PL in ethyl acetate at 65 K, respectively. The onsets were obtained by tangential method, i.e., the intersection of the tangent, set at the high energy slope of the spectrum, with the x-axis. Because the phosphorescence of UVA-2 was not observed, therefore E (T_1) of UVA-2 was referred to the literature, where E (T_1) was estimated from the phosphorescence quenching experiments with the quencher having E (T_1) = 2.59 eV.[8] E (T_1) of UVAs calculated by TD-DFT were also given.

Supplementary Table 1 Photophysical properties of PCs in ethyl acetate.[3] The photophysical rate constants were derived by experimental and computational methods in this work followed by the procedure that our group studied[2,3] under assumptions, i) $k_{nr,T1}$, $k_{r,T1}$ << k_{RISC} (i.e., $k_{nr,T1}$ ~ 0 and $k_{r,T1}$ ~ 0) and ii) k_{ISC} >> k_{RISC}.

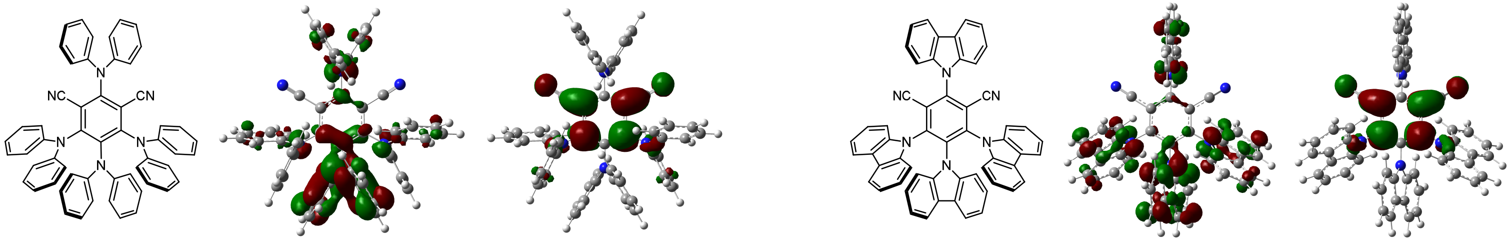

4DP-IPN HOMO LUMO 4Cz-IPN HOMO LUMO

PC	E (S$_1$) (eV)[a]	E (T$_1$) (eV)[b]	τ_{prompt} (ns)	$\tau_{delayed}$ (μs)	$\lambda_{max,abs}$ (nm)	$\lambda_{max,em}$ (nm)	$f_{S0\rightarrow S1}$[c]	Φ_F[d]	Φ_{PF}[e]	Φ_{DF}[f]	Φ_{ISC}[g]	Φ_{RISC}[h]	Kc[i]	$k_{r,S1}$ (10^7 s^{-1})	$k_{nr,S1}$ (10^7 s^{-1})	k_{ISC} (10^8 s^{-1})	$k_{r,T1}$ (s^{-1})	$k_{nr,T1}$ (10^5 s^{-1})	k_{RISC} (10^5 s^{-1})
4DP-IPN	2.55	2.38	2.11	74.67	465	518	0.0750	0.26	0.07 (0.04)[j]	0.19 (0.22)[j]	0.75	1.00	3.71	3.1 [k]	8.9	3.5	-	-	0.53
									(0.04)[j]	(0.22)[j]	0.96	0.89	6.50	1.9†	-	4.6†	-	0.10†	0.77†
4Cz-IPN	2.77	2.68	17.28	4.12	422	524	0.0742	0.64	0.47 (0.24)[j]	0.17 (0.40)[j]	0.26	1.00	1.36	2.7 [k]	1.5	0.15	-	-	3.3
									(0.24)[j]	(0.40)[j]	0.76	0.82	2.67	1.4†	-	0.44†	-	1.2†	5.3†

E_{00} were extracted from the [a]onset of PL and [b]onset of gated PL in ethyl acetate at 65 K, respectively. The onsets were obtained by tangential method, i.e., the intersection of the tangent, set at the high energy slope of the spectrum, with the x-axis. [c]Oscillator strengths were obtained by TD-DFT calculation. [d]Φ_F are relatively measured against the coumarin C153.[9] [e]Φ_{PF} are obtained from the following relationship, $\Phi_{PF} = k_{r,S1} / (k_{r,S1} + k_{nr,S1} + k_{ISC})$. [f]$\Phi_{DF}$ are obtained from the following relationship, $\Phi_{DF} = \Phi_F - \Phi_{PF}$. [g]$\Phi_{ISC}$ are obtained from the following relationship, $\Phi_{ISC} = k_{ISC} / (k_{r,S1} + k_{nr,S1} + k_{ISC})$. [h]$\Phi_{RISC}$ are obtained from the following relationship, $\Phi_{RISC} = k_{RISC} / (k_{r,T1} + k_{nr,T1} + k_{RISC})$. [i]$K_C$ are correlated with the number of TADF cycles determined from the following relationship, $K_C = \Phi_F / \Phi_{PF}$.[10] [j]Values in parentheses of both PCs were determined from the total PLQY (Φ_F) and the proportion of the integrated area of individual components in the TCSPC decay to the total integrated area.[11,12] [k]$k_{r,S1}$ was estimated via the Strickler-Berg equation.[13,14] †Photophysical values were obtained following by the procedure[15,16] under assumption of $k_{nr,S1}$ ~ 0 and $k_{r,T1}$ ~ 0.

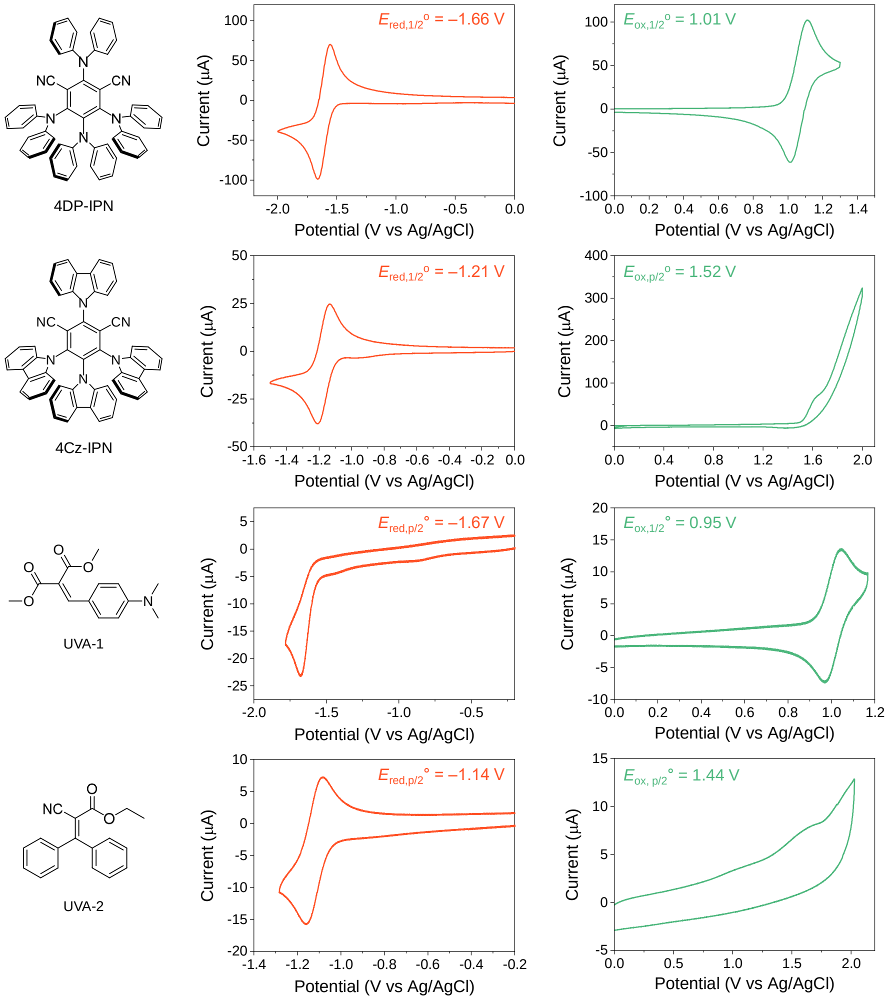

Supplementary Fig. 9 Chemical structures and CV spectra of PCs and UVAs studied in this work. CV spectra were obtained in CH_3CN (2.0×10^{-4} M) at RT after the degassing process by purging with 99.9999% argon for 15 min. For reversible CV cycle, the potentials were evaluated as their half-potentials ($E_{1/2}^{0}$), however, for irreversible CV cycle, the potentials were obtained from their half-peak potentials ($E_{p/2}^{0}$).[4] All potentials were converted to the SCE scale by using E^{0} (Fc^{+}/Fc) = 0.42 V vs SCE in CH_3CN.[4]

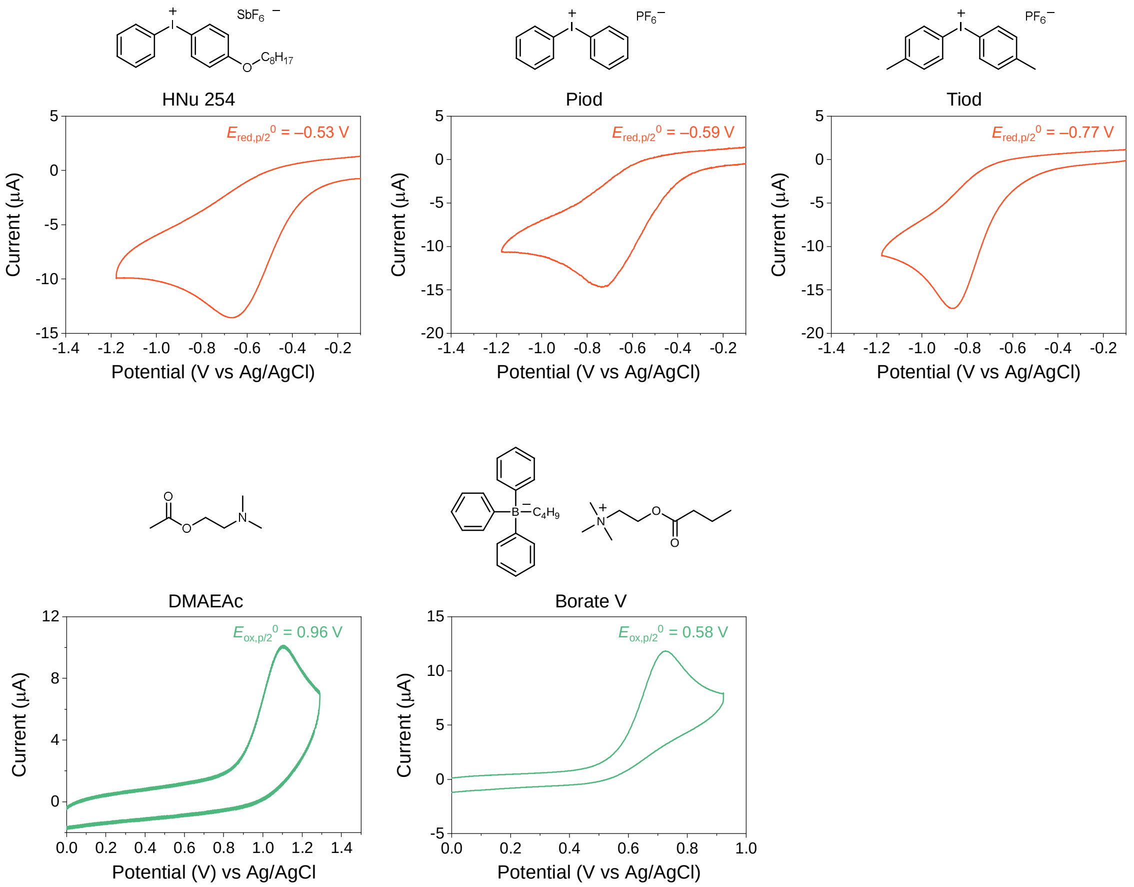

Supplementary Fig. 10 Chemical structures and CV spectra of co-initiators studied in this work. CV spectra were obtained in CH_3CN (2.0×10^{-4} M) at RT after the degassing process by purging with 99.9999% argon for 15 min. For irreversible CV cycle, the potentials were obtained from their half-peak potentials ($E_{p/2}^0$).[4] All potentials were converted to the SCE scale by using E^0 (Fc^+/Fc) = 0.42 V vs SCE in CH_3CN.[4]

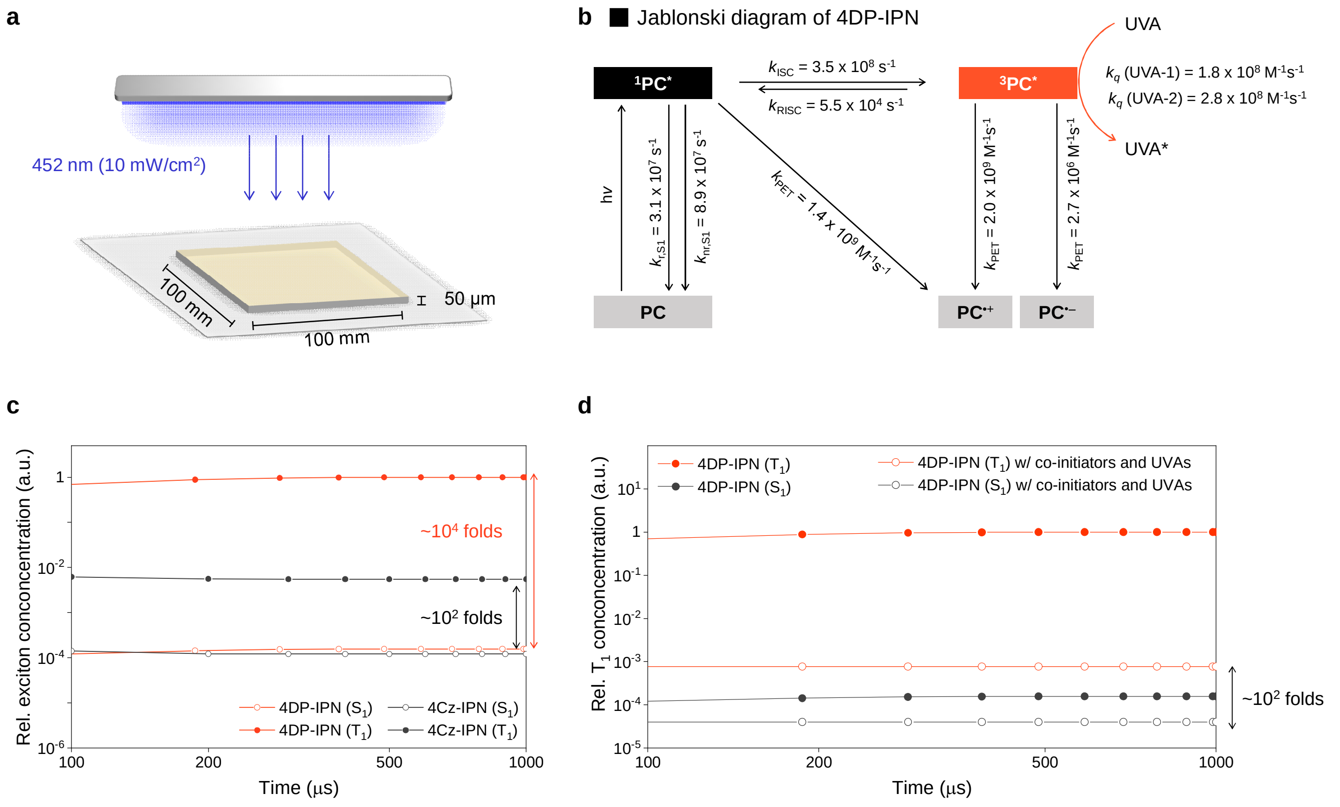

Supplementary Fig. 11 Comparison of contributions to photoinduced electron transfer (PET) from the singlet (S_1) and triplet (T_1) excited states. **a** Scheme of the kinetic simulation for the exciton population. **b** Jablonski diagram of 4DP-IPN in ethyl acetate including measured PET rate constants (k_{PET}, M^{-1} s^{-1}) between the PCs and quenchers (i.e., co-initiators and UVAs). These quenching rate constants for both excited states of the 4DP-IPN were determined by monitoring the changes in prompt fluorescence (PF) and delayed fluorescence (DF) using TCSPC techniques at λ_{ex} = 377 nm and λ_{det} = 520 nm (see Supplementary Fig. 12 and 13). **c** Simulated relative populations of the lowest S_1 and T_1 excited states of the 4DP-IPN and 4Cz-IPN in ethyl acetate (4.75 × 10^{-5} M) were calculated under the photostationary state during continuous illumination with a 452 nm LED at an intensity of 10 mW cm^{-2} (see Supplementary Note 2.3 for the more detail). **d** Relative population of T_1 (orange line) and S_1 state (black line) of 4DP-IPN (4.75 × 10^{-5} M) are simulated without (solid circle) or with UVA-1 (9.50 × 10^{-3} M), UVA-2 (3.04 × 10^{-2} M), HNu 254 (4.75 × 10^{-3} M) and Borate V (2.85 × 10^{-3} M) (hollow circle).

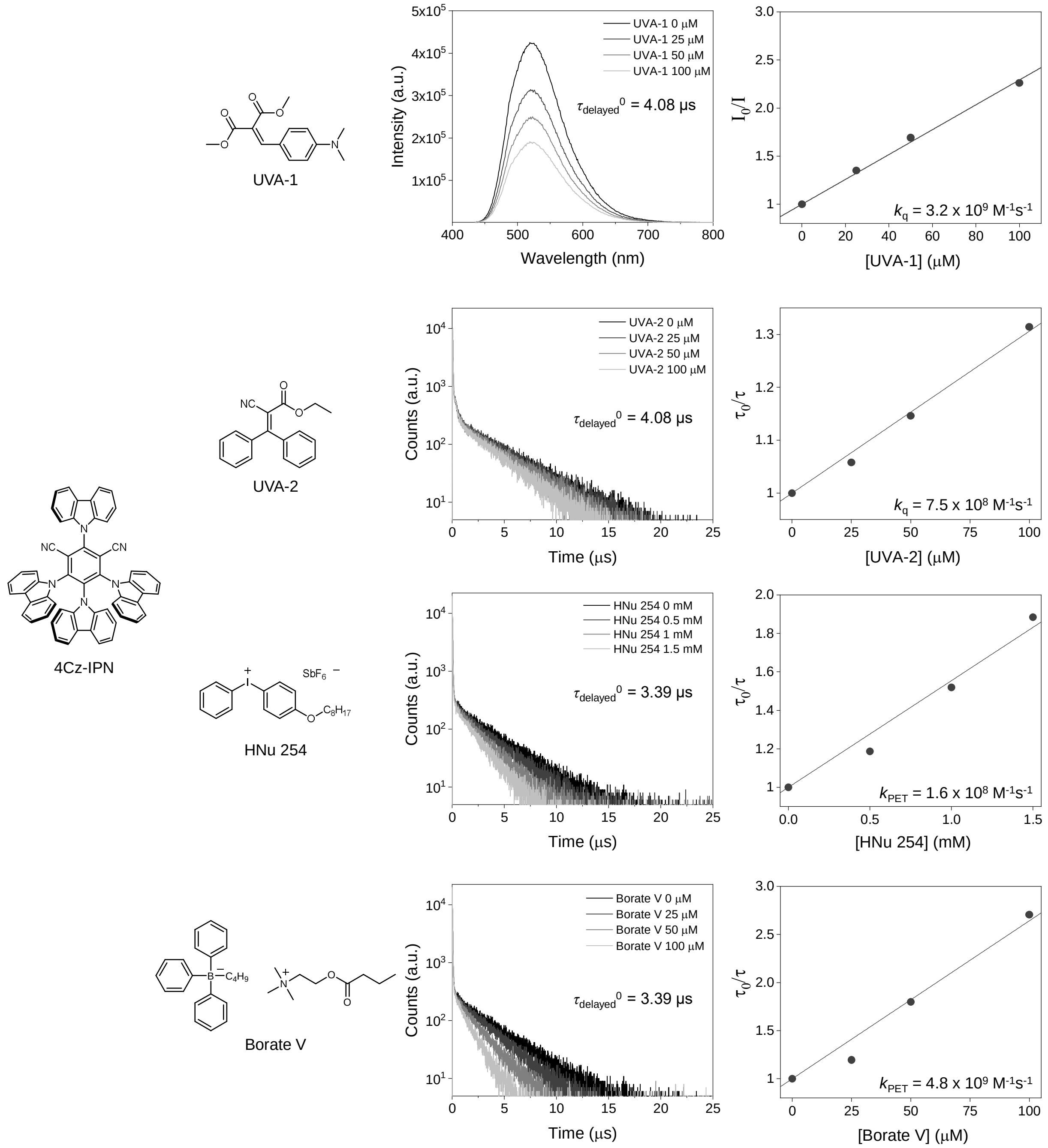

Supplementary Fig. 12 Chemical structures and Stern-Volmer plots of PL quenching of 4Cz-IPN at RT along with addition of UVAs and co-initiators; here PET denote photoinduced electron transfer. Generally, PL quenching experiments were conducted by measurements of the change of delayed fluorescence decay taken from the degassed solutions of 4Cz-IPN in ethyl acetate (1.0×10^{-5} M) varying the concentration of quenchers with TCSPC techniques monitored at λ_{ex} = 377 nm and λ_{det} = 520 nm. PL quenching experiments with UVA-1 were conducted by monitoring the change in intensity maxima of steady-state PL emission at λ_{ex} = 420 nm, as there was no intensity in TCSPC, this is probably because UVA-1 would predominantly absorb the excitation wavelength (λ_{ex} = 377 nm) used for TCSPC.

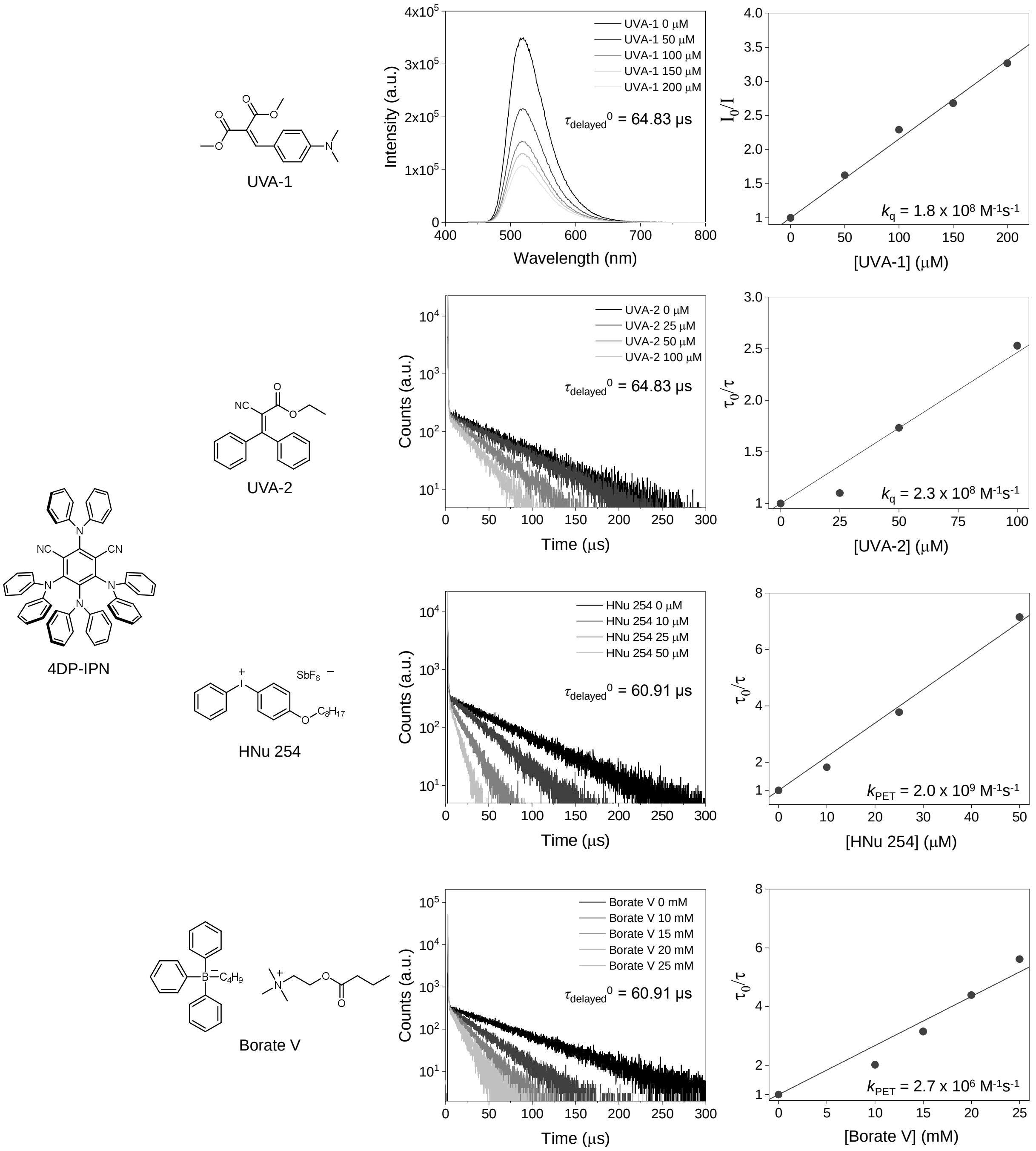

Supplementary Fig. 13 Chemical structures and Stern-Volmer plots of PL quenching of 4DP-IPN at RT along with addition of UVAs and co-initiators. Generally, PL quenching experiments were conducted by measurements of the change of delayed fluorescence decay taken from the degassed solutions of 4DP-IPN in ethyl acetate (1.0×10^{-5} M) varying the concentration of quenchers with TCSPC techniques monitored at λ_{ex} = 377 nm and λ_{det} = 520 nm. PL quenching experiments with UVA-1 were conducted by monitoring the change in intensity maxima of steady-state PL emission at λ_{ex} = 420 nm, as there was no intensity in TCSPC, this is probably because UVA-1 would predominantly absorb the excitation wavelength (λ_{ex} = 377 nm) used for TCSPC.

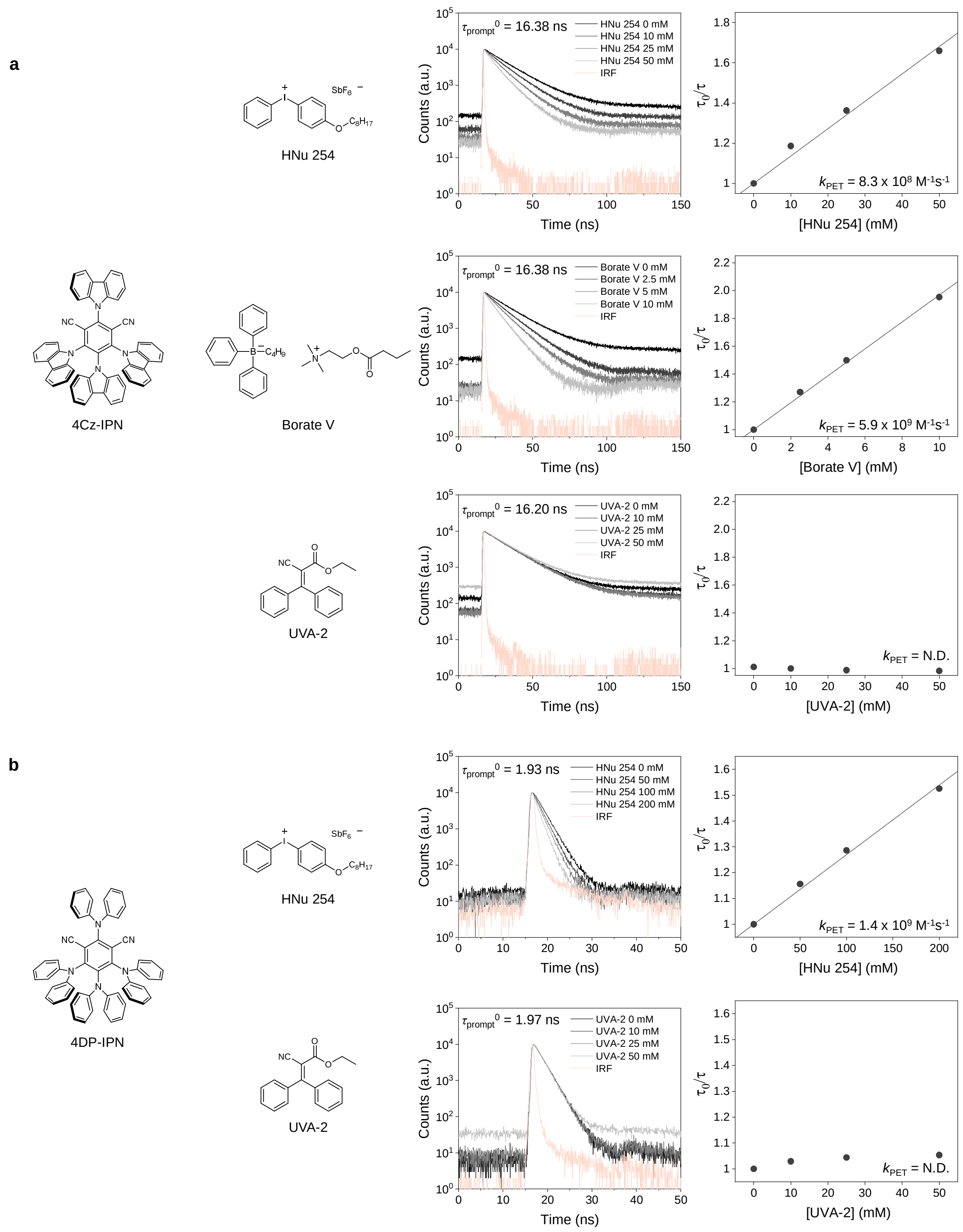

Supplementary Fig. 14 Chemical structures and Stern-Volmer plots of PL quenching of **a** 4Cz-IPN and **b** 4DP-IPN at RT along with addition of co-initiators. Generally, PL quenching experiments were conducted by measurements of the change of prompt fluorescence decay taken from the undegassed solutions of PCs in ethyl acetate (1.0×10^{-5} M) varying the concentration of co-initiators with TCSPC techniques monitored at λ_{ex} = 377 nm and λ_{det} = 520 nm. Stern-Volmer relationship between 4DP-IPN and Borate V were not determined which is attributed to slow PET rate constant being out of our measurement timescale. PL quenching experiments with UVA-1 were not conducted because UVA-1 would predominantly absorb the excitation wavelength (λ_{ex} = 377 nm) used for TCSPC.

Supplementary Table 2 Electronic structure of PCs and UVAs. Ionization potential (IP) and electron affinity (EA) of each species were evaluated from CV measurement in CH_3CN and DFT calculations on the optimized geometries of radical cation/anion in ethyl acetate employing B3LYP functional, 6–311++G* basis set and the PCM solvation model.

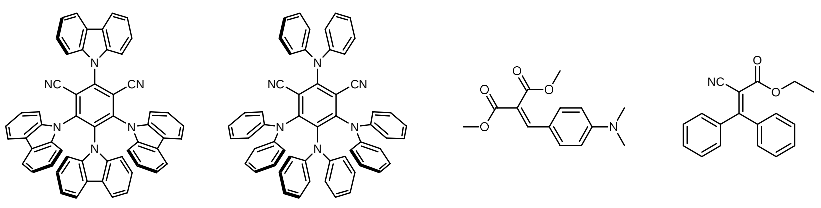

4Cz-IPN 4DP-IPN UVA-1 UVA-2

	IP (eV)			EA (eV)			E (T_1) (eV)	
	calc.[a]	calc.[b]	exp.[c]	calc.[a]	calc.[b]	exp.[c]	calc.[d]	exp.[e]
4Cz-IPN	6.05	6.04	5.90	2.98	3.26	3.17	2.34	2.68
4DP-IPN	5.61	5.57	5.39	2.56	2.71	2.72	2.21	2.38
UVA-1	5.91	5.85	5.33	2.07	2.39	2.71	2.37	2.36
UVA-2	6.87	6.83	5.82	2.72	3.04	3.24	2.50	< 2.59 [f]

a: IP and EA were computationally evaluated from energy level of HOMO and LUMO by Koopmans' theorem.
b: IP and EA were computationally evaluated from calculated free energy differences between the radical cation/anion and neutral state, respectively.
c: IP and EA were experimentally evaluated from CV measurements converted by IP = E_{ox}^0 (V vs SCE) + 4.8 eV - 0.42 eV and EA = E_{red}^0 (V vs SCE) + 4.8 eV - 0.42 eV, respectively. This value 4.8 eV is the HOMO energy level of ferrocene against vacuum[17] and 0.42 eV is the half-potential of ferrocene against SCE calibrated for CV measurements in this work.[4]
d: E (T_1) were computationally evaluated from TD-DFT for vertical transition (from S_0 to T_1) obtained by single-point calculations on the optimized geometry of neutral state.
e: E (T_1) were experimentally evaluated from the onset of gated PL emission spectra in ethyl acetate at low temperature (66 or 77K).
f: E (T_1) of UVA-2 was referred to the literature.[8]

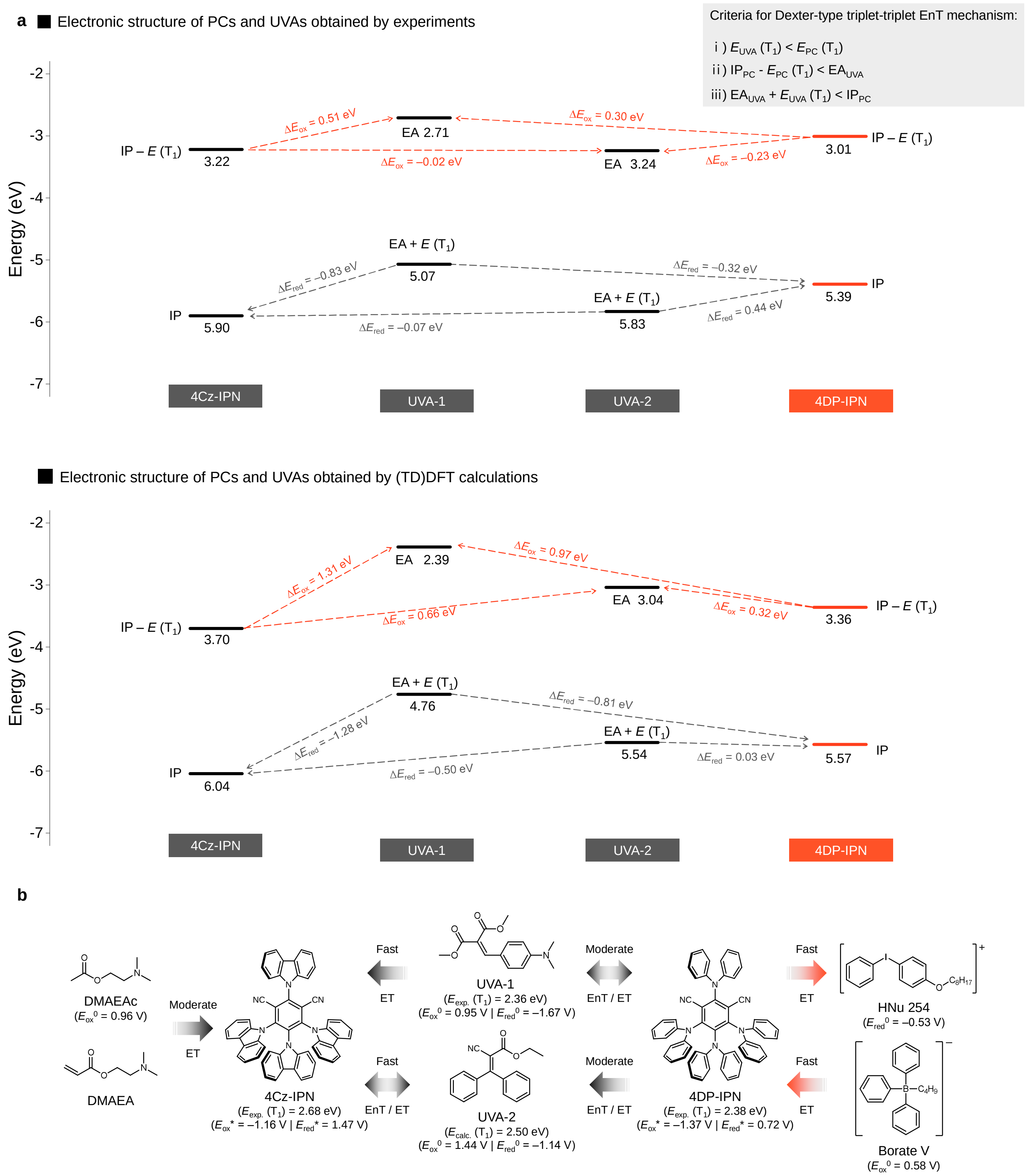

Supplementary Fig. 15 Schematic illustration for interactions between PCs and UVAs. **a** Electronic structure of PCs and UVAs obtained by experiments (upper part) and (TD)DFT calculations (lower part). The driving forces (i.e., ΔE_{ox} and ΔE_{red}) for each step of electron exchange in Dexter-type EnT between $^3PC^*$ and UVAs are given; initial transfer pathway (orange dashed line) and following transfer pathway (dark grey dashed line). The energy values (i.e., IP, EA and E (T_1)) obtained by experiments (i.e., CV measurement and onset of gated PL emission at low temperature) and (TD)DFT calculations are listed in Supplementary Table 2. **b** Schematic illustration of the reaction pathways of PCs in the excited state including energy transfer (EnT) or electron transfer (ET) process with UVAs or co-initiators studied in this work.

Supplementary Table 3 Experimental evaluation of driving forces for PET between PCs and UVAs. It is notable that errors from experimental or computational method would be inherent.

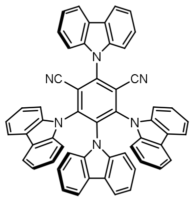

4Cz-IPN

$E_{ox,\ exp.}$* (S_1) = –1.25 V $E_{ox,\ exp.}$* (T_1) = –1.16 V
$E_{red,\ exp.}$* (S_1) = 1.56 V $E_{red,\ exp.}$* (T_1) = 1.47 V

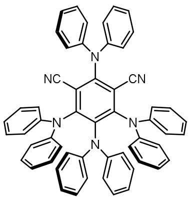

4DP-IPN

$E_{ox,\ exp.}$* (S_1) = –1.54 V $E_{ox,\ exp.}$* (T_1) = –1.37 V
$E_{red,\ exp.}$* (S_1) = 0.89 V $E_{red,\ exp.}$* (T_1) = 0.72 V

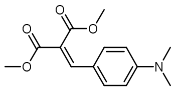

UVA-1

E_{ox}^0 = 0.95 V
E_{red}^0 = –1.67 V

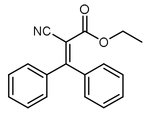

UVA-2

E_{ox}^0 = 1.44 V
E_{red}^0 = –1.14 V

–ΔG (eV)		Oxidative quenching		Reductive quenching	
PC	UVA	from ^{1}PC*	from ^{3}PC*	to ^{1}PC*	to ^{3}PC*
4Cz-IPN	UVA-1	–0.42	–0.51	0.61	0.52
	UVA-2	0.11	0.02	0.12	0.03
4DP-IPN	UVA-1	–0.13	–0.30	–0.06	–0.23
	UVA-2	0.40	0.23	–0.55	–0.72

2.3. Computational characterization of PIS: kinetic simulation

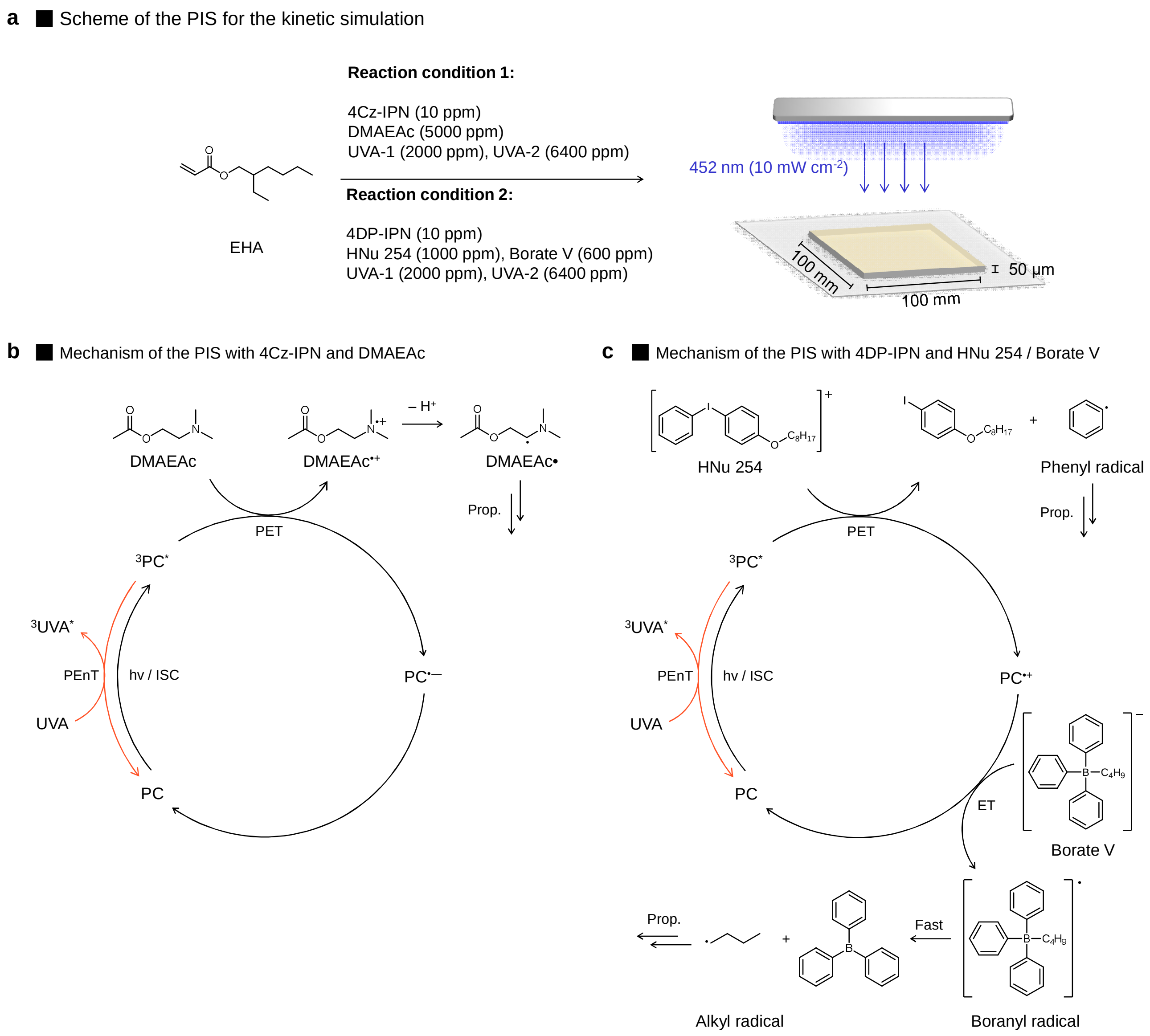

Supplementary Fig. 16 Kinetic simulation of visible-driven PIS. **a** Scheme of the kinetic simulation for each PIS. **b** Proposed mechanism of the PIS with 4Cz-IPN and DMAEAc. **c** Proposed mechanisms of the PIS with 4DP-IPN, HNu 254 and Borate V.

Kinetic simulations for the homopolymerization of EHA were performed based on the rate law (Supplementary Table 4 and 5). To simplify the kinetic simulation, we involved the following assumptions; 1) internal conversion (IC) is fast compared to our time scale of interest, 2) a main contributor to PET or photoinduced triplet-triplet energy transfer (PEnT) with co-initiators or UVAs is $^3PC^*$, and then it is assumed that $^1PC^*$ have negligibly small contribution to PET or PEnT, 3) side-reactions such as triplet-triplet annihilation and photodegradation of PC are not considered, 4) no back-electron transfer (BET) is considered because spin-flipping is forbidden where $^3PC^*$ are involved,[18] 5) PEnT between PCs and UVAs is considered as main $^3PC^*$ quenching process and then the generated $^1UVA^*$ is unreactive, 6) oxygen species (e.g., $^{1/3}O_2$ and $O_2^{\bullet -}$) are not involved, and 7) in the kinetic simulation of 4Cz-IPN and DMAEAc, there is no reaction to close the photocatalytic cycle, nevertheless there is no significant error as the only tiny amount of 4Cz-IPN and DMAEAc are consumed at initial kinetics (< 100 ms). For the photocatalytic cycle of 4DP-IPN, although both oxidative and reductive quenching cycle are involved in the kinetic simulation, but only the oxidative quenching cycle are described in Supplementary Fig. 16c as it mainly attributes to PIS.

Supplementary Table 4 General form of mass balance equations for all species in visible-light driven PIS.

Species	Mass balance equations
PC (S_0)	$\frac{d[S_0]}{dt} = -k_{abs}\left(1-10^{-\varepsilon\times l\times[S_0]}\right) + \left(k_{r,S_1}+k_{nr,S_1}\right)[S_1] + k_{ET,1}[DC][Borate\ V] + k_{ET,2}[DA][HNu\ 254] + k_{q,1}[T_1][UVA1] + k_{q,2}[T_1][UVA2]$
$^1PC^*$ (S_1)	$\frac{d[S_1]}{dt} = k_{abs}(1-10^{-\varepsilon\times l\times[S_0]}) + k_{RISC}[T_1] - (k_{ISC}+k_{nr,S_1}+k_{nr,S_1})[S_1]$
$^3PC^*$ (T_1)	$\frac{d[T_1]}{dt} = k_{ISC}[S_1] - k_{RISC}[T_1] - k_{PET,1}[T_1][HNu254] - k_{PET,2}[T_1][Borate\ V] - k_{PET,3}[T_1][DMAEAc] - k_{q,1}[T_1][UVA1] - k_{q,2}[T_1][UVA2]$
$^2PC^{\bullet+}$ (DC)	$\frac{d[DC]}{dt} = k_{PET,1}[T_1][HNu254] - k_{ET,1}[DC][Borate\ V]$
$^2PC^{\bullet-}$ (DA)	$\frac{d[DA]}{dt} = k_{PET,2}[T_1][Borate\ V] - k_{ET,2}[DA][HNu\ 254]$
HNu 254	$\frac{d[HNu254]}{dt} = -k_{PET,1}[T_1][HNu254] - k_{ET,2}[DA][HNu\ 254]$
Phenyl radical (PR)	$\frac{d[PR]}{dt} = k_{PET,1}[T_1][HNu254] + k_{ET,2}[DA][HNu\ 254]$
Borate V	$\frac{d[Borate\ V]}{dt} = -k_{ET,1}[DC][Borate\ V] - k_{PET,2}[T_1][Borate\ V]$
Boranyl radical (BR)	$\frac{d[BR]}{dt} = k_{ET,1}[DC][Borate\ V] + k_{PET,2}[T_1][Borate\ V] - k_{disso.}[BR]$
Alkyl radical (AR)	$\frac{d[AR]}{dt} = k_{disso.}[BR]$
DMAEAc	$\frac{d[DMAEAc]}{dt} = -k_{PET,3}[T_1][DMAEAc]$
$DMAEAc^{\bullet+}$(DRC)	$\frac{d[DRC]}{dt} = k_{PET,3}[T_1][DMAEAc] - k_{amino.}[DRC][DMAEAc]$
DMAEAc• (DAR)	$\frac{d[DAR]}{dt} = k_{amino.}[DRC][DMAEAc]$
UVA-1 (UVA1)	$\frac{d[UVA1]}{dt} = -k_{q,1}[T_1][UVA1]$
$^3UVA\text{-}1^*$ (UVA1*)	$\frac{d[UVA1^*]}{dt} = k_{q,1}[T_1][UVA1]$
UVA-2 (UVA2)	$\frac{d[UVA2]}{dt} = -k_{q,2}[T_1][UVA2]$
$^3UVA\text{-}2^*$ (UVA2*)	$\frac{d[UVA2^*]}{dt} = k_{q,2}[T_1][UVA2]$
Counts of radical generation (RG)	$\frac{d[RG]}{dt} = k_{PET,1}[T_1][HNu254] + k_{ET,2}[DA][HNu\ 254] + k_{disso.}[BR] + k_{amino.}[DRC]$

Supplementary Table 5 Rate constants for all reactions and the concentrations of each species in visible-light driven PIS for the kinetic simulation. All rate constants were obtained from experimental/computational methods[2,3] in the this work or were referred to literature.[19]

<table>
<tr><th rowspan="2">Reaction</th><th colspan="4">Previous PIS</th><th colspan="5">Newly designed PIS</th></tr>
<tr><th>4Cz-IPN (4.75×10^{-5} M)</th><th>DMAEAc (2.38×10^{-2} M)</th><th>UVA-1 (9.50×10^{-3} M)</th><th>UVA-2 (3.04×10^{-2} M)</th><th>4DP-IPN (4.75×10^{-5} M)</th><th>HNu 254 (4.75×10^{-3} M)</th><th>Borate V (2.85×10^{-3} M)</th><th>UVA-1 (9.50×10^{-3} M)</th><th>UVA-2 (3.04×10^{-2} M)</th></tr>
<tr><td>Photoexcitation: k_{abs} (a.u.)
$PC \rightarrow {}^1PC^*$</td><td colspan="4">7.6×10^{-4}</td><td colspan="5">7.6×10^{-4}</td></tr>
<tr><td>Radiative decay from S_1: k_r (s^{-1})
${}^1PC^* \rightarrow PC$</td><td colspan="4">2.7×10^{7} [a]</td><td colspan="5">3.1×10^{7} [a]</td></tr>
<tr><td>Non-radiative decay from S_1: k_{nr} (s^{-1})
${}^1PC^* \rightarrow PC$</td><td colspan="4">1.5×10^{7} [a]</td><td colspan="5">8.9×10^{7} [a]</td></tr>
<tr><td>Intersystem crossing from S_1 to T_1: k_{ISC} (s^{-1})
${}^1PC^* \rightarrow {}^3PC^*$</td><td colspan="4">$1.5 \times 10^{7}$ [a]</td><td colspan="5">3.5×10^{8} [a]</td></tr>
<tr><td>Reverse intersystem crossing from ${}^3PC^*$ to S_1: k_{RISC} (s^{-1})
${}^3PC^* \rightarrow {}^1PC^*$</td><td colspan="4">$3.3 \times 10^{5}$ [a]</td><td colspan="5">5.3×10^{4} [a]</td></tr>
<tr><td>PET from ${}^3PC^*$ to HNu 254: $k_{PET,1}$ (M^{-1} s^{-1})
${}^3PC^*$ + HNu 254 → ${}^2PC^{\bullet+}$ + Phenyl radical + Aryl iodide</td><td colspan="4">–</td><td colspan="5">2.0×10^{9}</td></tr>
<tr><td>ET from Borate V to ${}^2PC^{\bullet+}$: $k_{ET,1}$ (M^{-1} s^{-1})
${}^2PC^{\bullet+}$ + Borate V → PC + Boranyl radical</td><td colspan="4">–</td><td colspan="5">2.8×10^{7}</td></tr>
<tr><td>PET from Borate V to ${}^3PC^*$: $k_{PET,2}$ (M^{-1} s^{-1})
${}^3PC^*$ + Borate V → ${}^2PC^{\bullet-}$ + Boranyl radical</td><td colspan="4">–</td><td colspan="5">2.7×10^{6}</td></tr>
<tr><td>ET from ${}^2PC^{\bullet-}$ to HNu 254: $k_{ET,2}$ (M^{-1} s^{-1})
${}^2PC^{\bullet-}$ + HNu 254 → PC + Phenyl radical + Aryl iodide</td><td colspan="4">–</td><td colspan="5">6.5×10^{10}</td></tr>
<tr><td>PET from DMAEAc to ${}^3PC^*$: $k_{PET,3}$ (M^{-1} s^{-1})
${}^3PC^*$ + DMAEAc → ${}^2PC^{\bullet-}$ + DMAEAc$^{\bullet+}$</td><td colspan="4">2.1×10^{7}</td><td colspan="5">–</td></tr>
<tr><td>C-B bond dissociation of boranyl radical: $k_{disso.}$ (s^{-1})
Boranyl radical → Alkyl radical + Triphenyl borane</td><td colspan="4">–</td><td colspan="5">1.0×10^{11} [b]</td></tr>
<tr><td>PEnT from ${}^3PC^*$ to UVA-1: $k_{q,1}$ (M^{-1} s^{-1})
${}^3PC^*$ + UVA-1 → PC + ^{3}UVA-1*</td><td colspan="4">3.2×10^{9}</td><td colspan="5">1.8×10^{8}</td></tr>
<tr><td>PEnT from ${}^3PC^*$ to UVA-2: $k_{q,2}$ (M^{-1} s^{-1})
${}^3PC^*$ + UVA-2 → PC + ^{3}UVA-2*</td><td colspan="4">7.5×10^{8}</td><td colspan="5">2.3×10^{8}</td></tr>
<tr><td>Amino radical formation of DMAEAc$^{\bullet+}$: $k_{amino.}$ (M^{-1} s^{-1})
DMAEAc$^{\bullet+}$ + DMAEAc → DMAEAc-H$^+$ + DMAEAc •</td><td colspan="4">2.3×10^{5}</td><td colspan="5">–</td></tr>
</table>

a: Photophysical parameters of PCs were from Supplementary Table 1 obtained from experimental/computational methods.[2,3]
b: Rate constants of C-B bond dissociation in boranyl radical was approximated as ~ 1.0×10^{11} s^{-1}.[19]

■ **Derivation of k_{abs}**

Our LED setups are based on 452 nm (I_0 = 10 mW cm^{-2}) LEDs, therefore with consideration of photonflux (m^{-2} s^{-1}), P_n, the rate of S_n generation from S_0 via photoexcitation (i.e., $S_0 \rightarrow S_n$), can be expressed by following equation (3),[20,21]

$$P_n = \phi \times \frac{A}{V_0 N_A} \times \frac{I_0}{h\nu} \times \varepsilon c l \times F, \quad \text{where F is photo kinetic factor,} \; F = \frac{1-10^{-\varepsilon c l}}{\varepsilon c l} \tag{3}$$

where ϕ is quantum yield of the transformation for $S_0 \rightarrow S_n$, A is cross-sectional area (100 cm^2), V_0 is the reaction volume (5 mL), N_A is Avogadro number, h is Planck constant, ν is frequency of the photon, ε is the extinction coefficient of PCs in ethyl acetate (e.g., ε_{452nm} = 7.8 × 10^3 M^{-1} cm^{-1} for 4DP-IPN and ε_{452nm} = 2.1 × 10^3 M^{-1} cm^{-1} for 4Cz-IPN), c is the concentration of PC (i.e., [S_0] = 4.75 × 10^{-5} M for the 10 ppm in the synthesis of poly(EHA)) and l is the optical path length (50 μm). Because it is tricky to evaluate all the factors affecting photonflux (e.g., refractive index and surface curvature of release film), we excluded them in this kinetic simulation. Furthermore, as the internal conversion (i.e., $S_n \rightarrow S_1$) is highly fast, we would assume the lowest S_1 state are mainly generated. Quantum yield (ϕ) for $S_0 \rightarrow S_1$ is assumed as unity, hence, in accordance with our experimental conditions, equation (3) can be converted to equation (4).

$$P_1 = k_{\text{abs}} \times \left(1 - 10^{-0.005\,cm \times \varepsilon_{452\text{nm}} \times [S_0]}\right) = 7.6 \times 10^{-4}\ \text{M s}^{-1} \times \left(1 - 10^{-0.005\,cm \times \varepsilon_{452\text{nm}} \times [S_0]}\right) \tag{4}$$

■ **Evaluation of $k_{r,S1}$, $k_{nr,S1}$, k_{ISC} and k_{RISC}.**

The general TADF kinetics can be obtained from the differential equations for the singlet and triplet excited state (S_1 and T_1) deactivation.

$$\frac{d[S_1]}{dt} = P_1 + k_{\text{RISC}}[T_1] - k_S[S_1] \tag{5}$$
$$\frac{d[T_1]}{dt} = k_{\text{ISC}}[S_1] - k_T[T_1] \tag{6}$$

where P_1 is the rate of S_1 generation from S_0 via photoexcitation, $k_S = k_{r,S1} + k_{nr,S1} + k_{ISC}$ and $k_T = k_{r,T1} + k_{nr,T1} + k_{RISC}$; the solutions are described below

$$[S_1] = \frac{[S_1]_0}{A_1 - A_2}\left[(k_S - A_2)\,exp(-A_1 t) + (A_1 - k_S)\,exp(-A_2 t)\right] \tag{7}$$
$$[T_1] = \frac{[S_1]_0 \cdot k_{ISC}}{A_1 - A_2}\left[-exp(-A_1 t) + exp(-A_2 t)\right] \tag{8}$$

and the total (i.e. experimentally observed) luminescence time trace is given by[22]

$$I(t) = \Phi_F[S_1] + \Phi_{PH}[T_1] \tag{9}$$
$$= \frac{[S_1]_0}{A_1 - A_2}\left[(\Phi_F(k_S - A_2) - \Phi_{PH}k_{ISC})\,exp(-A_1 t) + (\Phi_F(A_1 - k_S) + \Phi_{PH}k_{ISC})\,exp(-A_2 t)\right] \tag{10}$$

The exponents $A_{1,2}$ (which correspond to the reciprocal values of the prompt/delayed PL lifetime constants, i.e., τ_{PF}^{-1} and τ_{DF}^{-1}, respectively) are given by

$$A_{1,2} = \frac{1}{2}\left(k_S + k_T \mp (k_T - k_S)\sqrt{1 + 4 \cdot k_{ISC}k_{RISC}/(k_T - k_S)^2}\right) \tag{11}$$

The total PL quantum yield Φ_{PL} is given as the sum of fluorescence and phosphorescence quantum yields ($\Phi_{PL} = \Phi_F + \Phi_{PH}$), where Φ_F consists of a prompt fluorescence (Φ_{PF}) and delayed fluorescence (Φ_{DF}) part; the prompt part is defined by

$$\Phi_{\mathrm{PF}} = \frac{k_{\mathrm{r,S_1}}}{k_{\mathrm{S}}} \tag{12}$$

In the presence of large number of TADF cycles, the total Φ_F under steady state is obtained as,[10]

$$\Phi_{\mathrm{F}} = \Phi_{\mathrm{PF}} \frac{1}{(1-\eta_{\mathrm{ISC}} \cdot \eta_{\mathrm{RISC}})} \tag{13}$$

where $\eta_{ISC} = k_{ISC}/k_S$ and $\eta_{RISC} = k_{RISC}/k_T$ are the efficiencies for ISC and RISC, respectively. Similarly, Φ_{PH} is obtained as

$$\Phi_{\mathrm{PH}} = \frac{\eta_{\mathrm{ISC}} k_{\mathrm{PH}}}{k_{\mathrm{T}}(1-\eta_{\mathrm{ISC}} \cdot \eta_{\mathrm{RISC}})} \tag{14}$$

The condition for strong TADF (i.e., large Φ_F and large Φ_{DF}/Φ_F) emitters translates to $k_{RISC} >> k_{r,T1}, k_{nr,T1}$, so that $\eta_{RISC} \approx 1$; this simplifies equation (13) to

$$\Phi_{\mathrm{F}} = \frac{\Phi_{\mathrm{PF}}}{(1-\eta_{\mathrm{ISC}} \cdot \eta_{\mathrm{RISC}})} \approx \frac{\Phi_{\mathrm{PF}}}{(1-\eta_{\mathrm{ISC}})} = \frac{k_{\mathrm{S}} \Phi_{\mathrm{PF}}}{(k_{\mathrm{r,S_1}} + k_{\mathrm{nr,S_1}})} = \frac{k_{\mathrm{r,S_1}}}{(k_{\mathrm{r,S_1}} + k_{\mathrm{nr,S_1}})} \tag{15}$$

Furthermore, for TADF compounds with a non-negligible ΔE_{ST}, RISC is much smaller than ISC (i.e., $k_{RISC} << k_{ISC}$).[2] Under these conditions, with a Taylor expansion ($y = \sqrt{1+x} \approx 1 + \frac{x}{2}$ for x << 1), the solutions for $A_{1,2}$ simplify to

$$A_1 = \tau_{\mathrm{PF}}^{-1} = \frac{1}{2}\left(k_{\mathrm{S}} + k_{\mathrm{T}} - (k_{\mathrm{T}} - k_{\mathrm{S}})\sqrt{1 + 4 \cdot k_{\mathrm{ISC}} k_{\mathrm{RISC}}/(k_{\mathrm{T}} - k_{\mathrm{S}})^2}\right) = k_{\mathrm{S}} - \frac{k_{\mathrm{ISC}} k_{\mathrm{RISC}}}{k_{\mathrm{T}} - k_{\mathrm{S}}} \approx k_{\mathrm{S}} \tag{16}$$

$$A_2 = \tau_{DF}^{-1} = \frac{1}{2}\left(k_S + k_T + (k_{\mathrm{T}} - k_{\mathrm{S}})\sqrt{1 + 4 \cdot k_{\mathrm{ISC}} k_{\mathrm{RISC}}/(k_{\mathrm{T}} - k_{\mathrm{S}})^2}\right) = k_T \left(1 - \frac{k_{\mathrm{ISC}} k_{\mathrm{RISC}}}{k_{\mathrm{T}} k_{\mathrm{S}}}\right) \approx k_{\mathrm{RISC}}(1 - \eta_{\mathrm{ISC}}) \tag{17}$$

Finally, the radiative rate constant $k_{\mathrm{r,S_1}}$ can be estimated from the Strickler-Berg formula, which in its simplified form reads,[13,14]

$$k_{\mathrm{r,S_1,SB}} = 0.667(\mathrm{s^{-1}cm^2}) \frac{E_{\mathrm{F,vert}}^3}{E_{\mathrm{A,vert}}} n^2 f = 4.34 \cdot 10^7 (\mathrm{s^{-1}eV^{-2}}) \frac{E_{\mathrm{F,vert}}^3}{E_{\mathrm{A,vert}}} n^2 f \tag{18}$$

where f is the TD-DFT calculated oscillator strength of vertical absorption, n is the refractive index of solvent and E is the energy of vertical absorption and emission respectively for the lowest energetic CT transition.

In summary, the photophysical rate constants of PCs in Jablonski diagram were evaluated by experimental (i.e., prompt/delayed fluorescence decays) and computational method (i.e., TD-DFT), which each relation is simplified to

$$k_{\mathrm{r,S_1}} = 4.34 \cdot 10^7 (\mathrm{s^{-1}eV^{-2}}) \frac{E_{\mathrm{F,vert}}^3}{E_{\mathrm{A,vert}}} n^2 f \tag{19}$$

$$k_{\mathrm{nr,S_1}} = \frac{k_{\mathrm{r,S_1}}}{\Phi_{\mathrm{F}}} - k_{\mathrm{r,S_1}} = k_{\mathrm{r,S_1}} \left(\frac{1}{\Phi_{\mathrm{F}}} - 1\right) \tag{20}$$

$$k_{\mathrm{ISC}} = \tau_{\mathrm{PF}}^{-1} - \frac{k_{\mathrm{r,S_1}}}{\Phi_{\mathrm{F}}} \tag{21}$$

$$k_{\mathrm{RISC}} = \frac{\tau_{\mathrm{DF}}^{-1}}{1 - k_{\mathrm{ISC}} \tau_{\mathrm{PF}}} \tag{22}$$

where f, n, E, Φ_F, τ_{PF}, and τ_{DF} have been defined earlier.

■ **Evaluation of k_{PET} and k_q**

To obtain the rate constant of PET between $^3PC^*$ and co-initiators and those of $^3PC^*$ quenching with UVAs, we monitored the change of delayed fluorescence of PCs along with addition of quenchers. In their Stern-Volmer plots (Supplementary Fig. 12 and 13), a linear relationship was observed.

■ **Evaluation of k_{ET}**

Because k_{ET} is the rate constants in dark reaction, it is tricky to estimate the rate constants from the direct experimental observation, therefore, we indirectly evaluated k_{ET} with computational and experimental methods (see below) using Marcus−Savéant theory where the rate constant of ET can be described by following equations,[23,24]

$$k_{ET} = Z\exp(-\frac{\Delta G^{\ddagger}}{RT}) \tag{23}$$

$$\Delta G^{\ddagger} = \frac{D_{R-X}+\lambda}{4}\left(1+\frac{\Delta G^{\circ}}{D_{R-X}+\lambda}\right)^2 \tag{24}$$

$$\Delta G^{\ddagger} \approx \frac{D_{R-X}}{4}\left(1+\frac{\Delta G^{\circ}}{D_{R-X}}\right)^2 \quad \text{for concerted ET} \tag{25}$$

$$\Delta G^{\ddagger} \approx \frac{\lambda}{4}\left(1+\frac{\Delta G^{\circ}}{\lambda}\right)^2 \quad \text{for stepwise ET} \tag{26}$$

where Z is the pre-exponential factor, $\Delta G^{\ddagger}$ is the activation energy of the reaction, ΔG° is the driving force of the reaction, D_{R-X} is the bond dissociation energy and λ is the external (solvent) reorganization energy.

■ **Evaluation of reorganization energy**

For the concerted ET process, the reorganization energy, λ, can be approximated to bond dissociation energy (D_{R-X}).[24] Therefore, to obtain the rate constant of ET between $PC^{\bullet-}$ and iodonium cation, the bond dissociation energy of iodonium was referred as D_{R-X} = 59.4 kcal mol^{-1}.[25] Meanwhile, for the stepwise ET between $PC^{\bullet+}$ and borate anion, because there is no significant change of molecular size of involved chemicals, internal reorganization energy, λ_i, is fairly negligible. Hence, the sum of reorganization energy, λ, was approximated to only external (solvent) reorganization energy, $\lambda = \lambda_i + \lambda_o \approx \lambda_o$.[23]

$$\lambda_o = \frac{e^2}{4\pi\varepsilon_0}\left(\frac{1}{\varepsilon_{op}}-\frac{1}{\varepsilon_s}\right)\left(\frac{1}{2r_A}+\frac{1}{2r_B}-\frac{1}{r_A+r_B}\right) \tag{27}$$

where e is the electron charge, ε_0 is the vacuum permittivity, ε_{op} and ε_s are the solvent optical and static dielectric constants, respectively. The r_s are the radii of the equivalent spheres of the subscript species. We calculated the radii of each species (Supplementary Fig. 17) with DFT calculations.

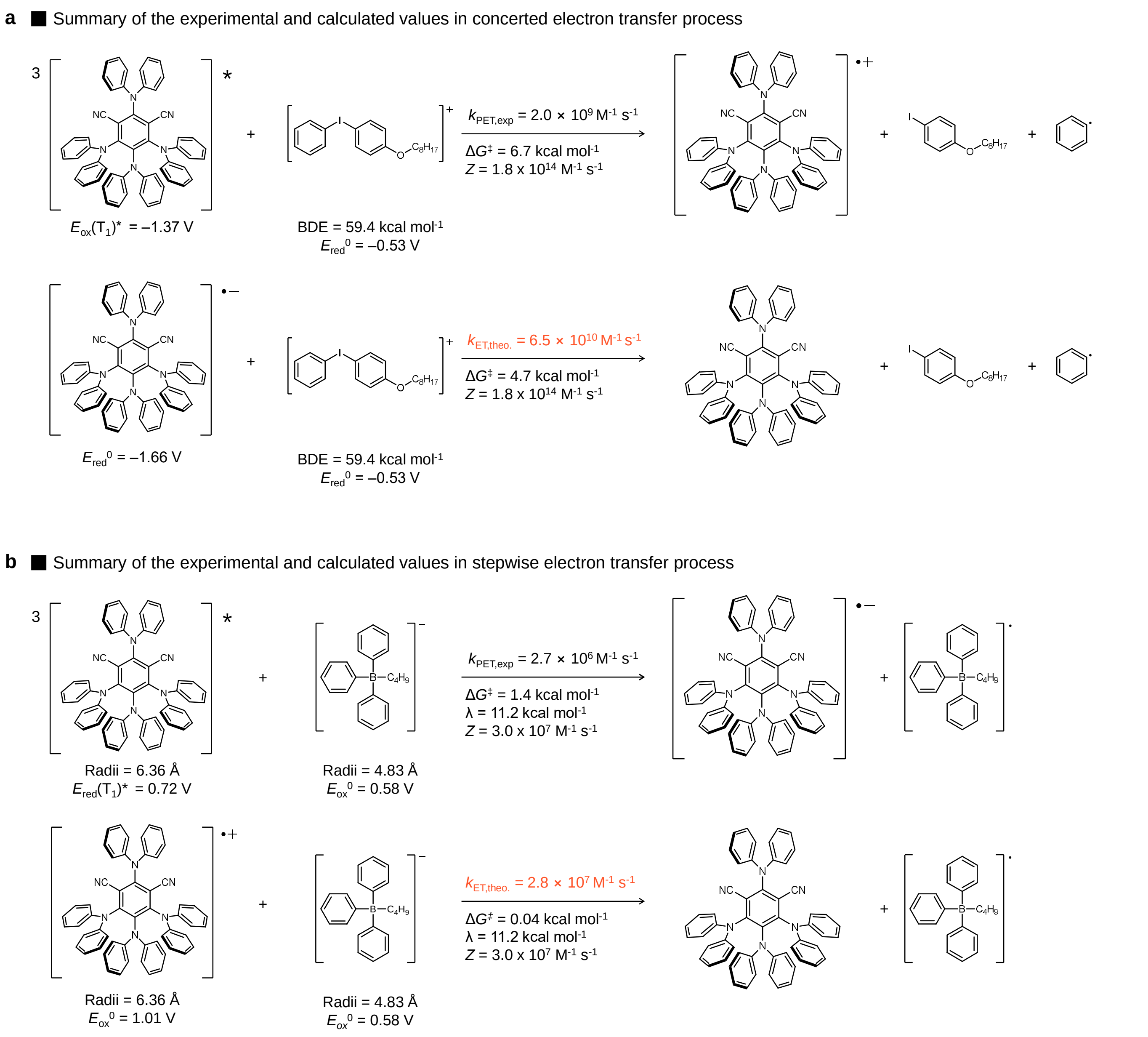

Supplementary Fig. 17 Summary of the experimental and calculated values to obtain the rate constant for ET between 4DP-IPN and co-initiators. **a** Summary of the experimental and calculated values in concerted ET process. **b** Summary of the experimental and calculated values in stepwise ET process. Molecular radii of each species are calculated by DFT calculations using the Gaussian16 program package, Z is the pre-exponential factor, $\Delta G^{\ddagger}$ is the activation energy of the ET reaction, and λ is the external (solvent) reorganization energy.

■ Evaluation of pre-exponential factor, *Z*

Because the size difference among PC species (i.e., $^3PC^*$, $PC^{\bullet-}$ and $PC^{\bullet+}$) is negligible, the pre-exponential factor, *Z*, for ET between PC species and co-initiators can be obtained from the rate constant for PET between $^3PC^*$ to co-initiators within reductive and oxidative quenching cycle, respectively (Supplementary Fig. 17).[2] The equations (23–26) can provide the pre-exponential factors for PET between $^3PC^*$ and co-initiators using the reorganization energy, λ, and the driving force, ΔG. These pre-exponential factor values should be same for the ET between $^3PC^*$ and co-initiators in the same manner. It is notable that the previously reported pre-exponential factor, *Z*, is ~10^{11} M^{-1} s^{-1},[26,27] thus, our k_{ET} might be under/overestimated and this discrepancy might be from the inaccurate extraction of values from experimental results. Nevertheless, our kinetic simulations were appropriately modeled to analyze the relative population of each species.

■ **Evaluation of $k_{amino.}$**

The α-amino radical species are generated from the oxidized tertiary amines associated with intramolecular proton transfer to neutral tertiary amines.[28] Hence, we evaluated the bimolecular rate constant from the activation energy ($\Delta G^{\ddagger}_{calc}$) for α-amino radical generation at RT using following Eyring equation (Supplementary Fig. 18).[3]

$$k = \kappa \frac{k_B T}{h} e^{-\frac{\Delta G^{\ddagger}}{RT}} \quad (28)$$

where k_B is the Boltzmann constant, T is the temperature, h is the Planck constant, and κ is the transmission coefficient and is assumed as a unity. In the case of DMAEAc, the activation energy is calculated as $\Delta G^{\ddagger}_{calc.}$ = 10.15 kcal mol^{-1} and the rate constant of α-amino radical generation is evaluated as $k_{amino.}$ = 2.3 × 10^5 M^{-1} s^{-1}.

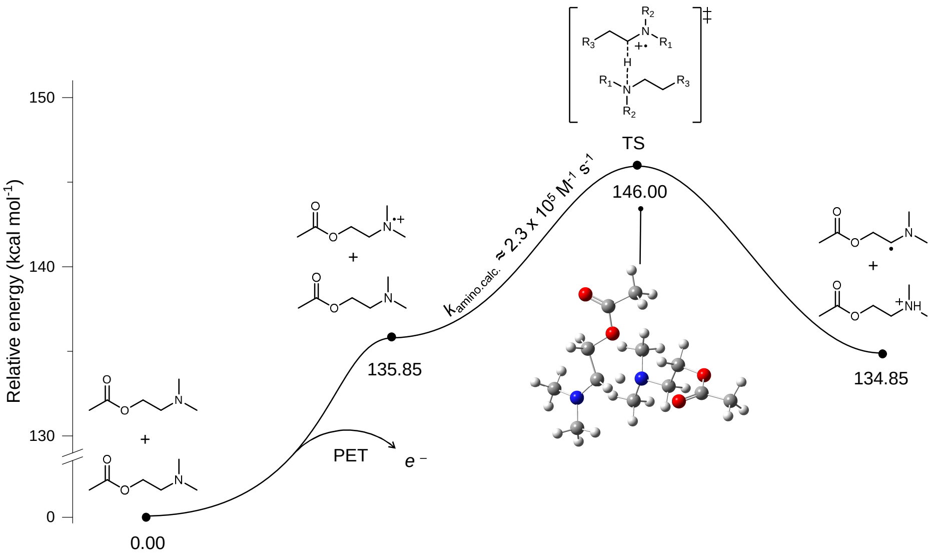

Supplementary Fig. 18 Energy profiles for α-amino radical generation from DMAEAc$^{•+}$ using DFT calculation. The rate constant for α-amino radical generation was evaluated with Eyring equation at RT.

Supplementary Note 3. Visible-light driven PIS for synthesis of UV-blocking OCA

3.1. Characterization of OCA film

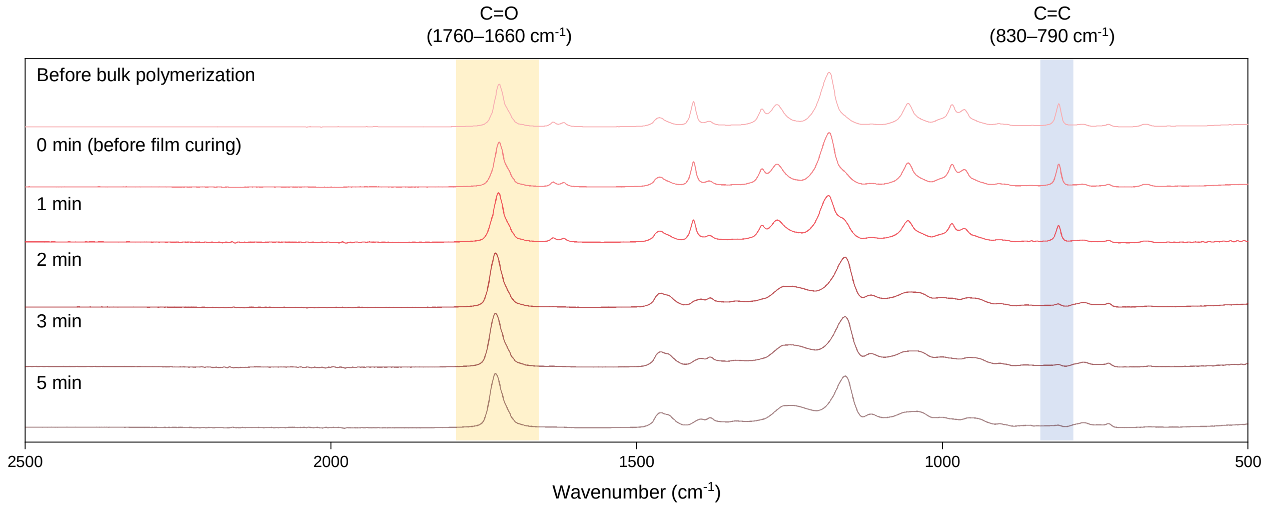

Supplementary Fig. 19 FR-IR spectra of a representative OCA preparation. The prepared OCA was conducted with [EHA]:[HBA] = 3:1 using 4DP-IPN (3 ppm), HNu 254 (1000 ppm) and Borate V (600 ppm).

Conversion of monomers from FT-IR spectra was calculated by following equation (29),

$$\text{Conversion (\%)} = \frac{\frac{A_{0(C=C)}}{A_{0(C=O)}} - \frac{A_{t(C=C)}}{A_{t(C=O)}}}{\frac{A_{0(C=C)}}{A_{0(C=O)}}} \times 100 \quad (29)$$

where $A_{0(C=C)}$, $A_{0(C=O)}$, $A_{t(C=C)}$ and $A_{t(C=O)}$, denote integrated area of representative peaks of C=C (830-790 cm^{-1}) at initial time, C =O (1760-1660 cm^{-1}) at initial time, C=C at time *t*, and C=O time at time *t*, respectively. If the integrated area is negative, the monomer conversion was approximated as 100%.

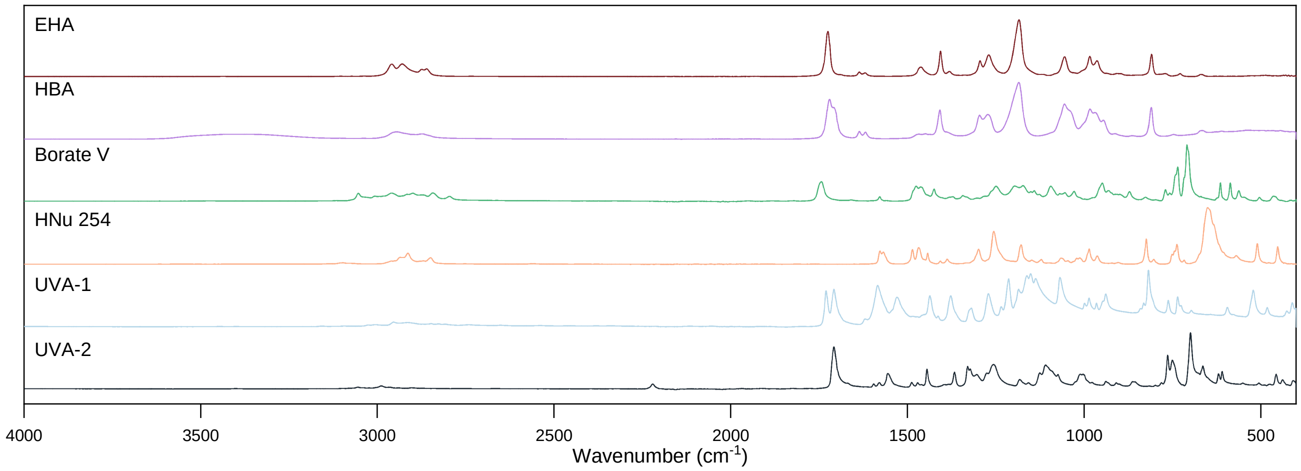

Supplementary Fig. 20 FT-IR spectra data of monomers, co-initiators and UVAs studied in this work.

3.2. Synthesis of UV-blocking OCA

Supplementary Table 6 Results of the previous PIS (with [BA]:[HBA] = 4:1) for UV-blocking OCAs using 4Cz-IPN and tertiary amines. PIS reaction conditions were followed the general procedures to prepare acrylic syrup and film curing. All conversions were determined by FT-IR.

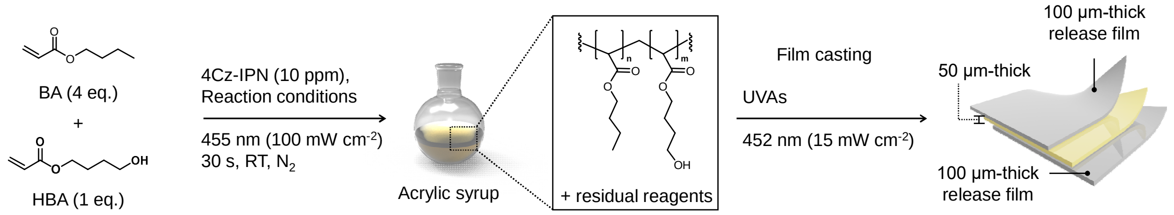

Entry	Co-initiator (ppm)	UVA (ppm)		Time $_{\text{Film}}$ (min)	Dosage $_{\text{Film}}$ (mJ cm^{-2})	Conversion $_{\text{FT-IR}}$ (%)	
		UVA-1	UVA-2			Acrylic syrup	Film
1	DMAEAc (5000)	–	–	4	3600	10.7[a]	95.1
2	DMAEAc (5000)	1500 (0.3 phr)	–	10	9000	10.7[a]	97.7
3	DMAEAc (5000)	–	4800 (1 phr)	20	18000	10.7[a]	96.3
4	DMAEAc (5000)	1500 (0.3 phr)	4800 (1 phr)	30	27000	10.7[a]	95.7
5	DMAEAc (5000)	–	–	5	3000[b]	11.4	94.4[b]
6	DMAEAc (5000)	1500 (0.3 phr)	–	20	12000[b]	11.4	94.1[b]
7	DMAEAc (5000)	–	4800 (1 phr)	20	12000[b]	11.4	95.3[b]
8	DMAEAc (5000)	1500 (0.3 phr)	4800 (1 phr)	30	18000[b]	11.4	94.7[b]
9	DMAEAc (2000) DMAEA (3000)	–	–	6	5400	11.9	95.9
10	DMAEAc (2000) DMAEA (3000)	1500 (0.3 phr)	4800 (1 phr)	30	27000	11.9	96.1

a: bulk polymerization was conducted under air in closed vial.
b: film curing was conducted under 452 nm (10 mW cm^{-2}).

Supplementary Table 7 Results of the newly designed PIS (with [EHA]:[HBA] = 3:1) using 4Cz-IPN, HNu 254 and Borate V. PIS reaction conditions were followed the general procedures to prepare acrylic syrup and film curing. All conversions were determined by FT-IR.

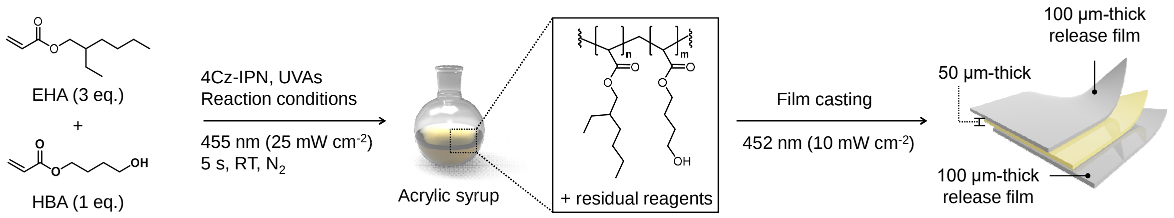

Entry	PC	PC loading (ppm)	Co-initiator (ppm)		UVA (ppm)		Dosage Film (mJ cm^{-2})	Conversion FT-IR (%)	
			HNu 254	Borate V	UVA-1	UVA-2		Acrylic syrup	Film
1	4Cz-IPN	1	500	300	–	–	6000	2.4	96.6
2	4Cz-IPN	1	500	300	2000 (0.3 phr)	–	12000	1.0	98.0
3	4Cz-IPN	1	500	300	–	6400 (1 phr)	12000	1.2	100
4	4Cz-IPN	1	500	300	2000 (0.3 phr)	6400 (1 phr)	18000	2.2[a]	100
5	4Cz-IPN	10	500	300	–	–	3000	10.3	94.3
6	4Cz-IPN	10	500	300	2000 (0.3 phr)	–	4800	2.1	96.9
7	4Cz-IPN	10	500	300	–	6400 (1 phr)	4800	5.1	93.4
8	4Cz-IPN	10	500	300	2000 (0.3 phr)	6400 (1 phr)	6000	4.6[a]	97.7
9	4Cz-IPN	10	1000	600	–	–	1800	10.9	98.9
10	4Cz-IPN	10	1000	600	2000 (0.3 phr)	–	3000	3.3	98.6
11	4Cz-IPN	10	1000	600	–	6400 (1 phr)	3000	5.2	97.3
12	4Cz-IPN	10	1000	600	2000 (0.3 phr)	6400 (1 phr)	3600	7.6[a]	99.3

a: bulk polymerization was conducted for 10 s due to slow polymerization.

Supplementary Table 8 Results of the newly designed PIS (with [EHA]:[HBA] = 3:1) using 4DP-IPN (1–3 ppm), HNu 254 (500–1000 ppm) and Borate V (300–600 ppm). PIS reaction conditions were followed the general procedures to prepare acrylic syrup and film curing. All conversions were determined by FT-IR.

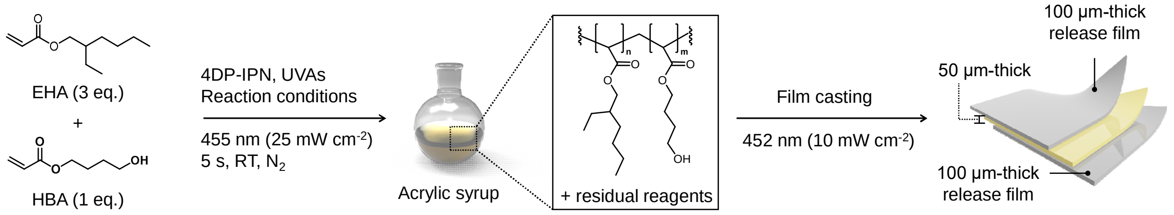

Entry	PC	PC loading (ppm)	Co-initiator (ppm)		UVA (ppm)		Dosage $_{Film}$ (mJ cm^{-2})	Conversion $_{FT-IR}$ (%)	
			HNu 254	Borate V	UVA-1	UVA-2		Acrylic syrup	Film
1	4DP-IPN	1	500	300	–	–	3000	9.6	97.5
2	4DP-IPN	1	500	300	2000 (0.3 phr)	–	6000	3.5	99.5
3	4DP-IPN	1	500	300	–	6400 (1 phr)	9000	2.3	96.2
4	4DP-IPN	1	500	300	2000 (0.3 phr)	6400 (1 phr)	6000	6.0[a]	97.4
5	4DP-IPN	2	1000	600	–	–	1800	19.3	98.5
6	4DP-IPN	2	1000	600	2000 (0.3 phr)	–	1800	15.9	94.9
7	4DP-IPN	2	1000	600	–	6400 (1 phr)	3000	16.7	94.9
8	4DP-IPN	2	1000	600	2000 (0.3 phr)	6400 (1 phr)	3000	10.8	98.0
9	4DP-IPN	3	1000	600	–	–	600	13.5[b]	97.8
10	4DP-IPN	3	1000	600	2000 (0.3 phr)	–	1800	9.5	100
11	4DP-IPN	3	1000	600	–	6400 (1 phr)	3000	8.9	100
12	4DP-IPN	3	1000	600	2000 (0.3 phr)	6400 (1 phr)	2400	8.3	97.8

a: bulk polymerization was conducted for 10 s due to slow polymerization.
b: bulk polymerization was conducted for 2 s due to fast polymerization.

Supplementary Table 9 Results of control experiments in the newly designed PIS (with [EHA]:[HBA] = 3:1) using PC (4DP-IPN (3 ppm) or 4Cz-IPN (10 ppm)), HNu 254 (1000 ppm) and Borate V (600 ppm). PIS reaction conditions were followed the general procedures to prepare acrylic syrup and film curing. All conversions were determined by FT-IR.

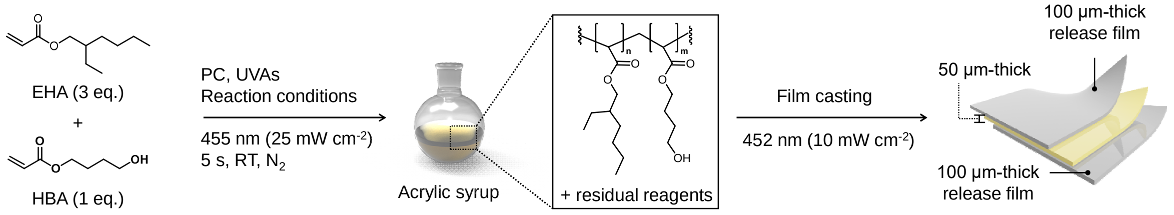

Entry	PC	PC loading (ppm)	Co-initiator (ppm)		UVA (ppm)		Dosage $_{Film}$ (mJ cm^{-2})	Conversion $_{FT-IR}$ (%)	
			HNu 254	Borate V	UVA-1	UVA-2		Acrylic syrup	Film
1	4DP-IPN	3	1000	–	–	–	2400	2.2	41.8
2	4DP-IPN	3	1000	–	2000 (0.3 phr)	–	2400	2.4	65.1
3	4DP-IPN	3	1000	–	–	6400 (1 phr)	2400	2.4	3.0
4	4DP-IPN	3	1000	–	2000 (0.3 phr)	6400 (1 phr)	2400	0.3	55.1
5	4DP-IPN	3	–	600	–	–	2400	0.3	0.8
6	4DP-IPN	3	–	600	2000 (0.3 phr)	–	2400	0.3	1.9
7	4DP-IPN	3	–	600	–	6400 (1 phr)	2400	0.2	1.5
8	4DP-IPN	3	–	600	2000 (0.3 phr)	6400 (1 phr)	2400	0.3	0.6
9	4Cz-IPN	10	1000	–	–	–	2400	1.9	80.7
10	4Cz-IPN	10	1000	–	2000 (0.3 phr)	–	2400	0.3	25.6
11	4Cz-IPN	10	1000	–	–	6400 (1 phr)	2400	1.0	19.7
12	4Cz-IPN	10	1000	–	2000 (0.3 phr)	6400 (1 phr)	2400	0.8	20.6
13	4Cz-IPN	10	–	600	–	–	2400	0.9	61.9
14	4Cz-IPN	10	–	600	2000 (0.3 phr)	–	2400	0.2	22.5
15	4Cz-IPN	10	–	600	–	6400 (1 phr)	2400	0.7	7.6
16	4Cz-IPN	10	–	600	2000 (0.3 phr)	6400 (1 phr)	2400	0.3	14.1

Supplementary Table 10 Reproducibility test in the previous PIS (with [BA]:[HBA] = 4:1) using 4Cz-IPN (10 ppm) and tertiary amines (5000 ppm). PIS reaction conditions were followed the general procedures to prepare acrylic syrup and film curing. All conversions were determined by FT-IR.

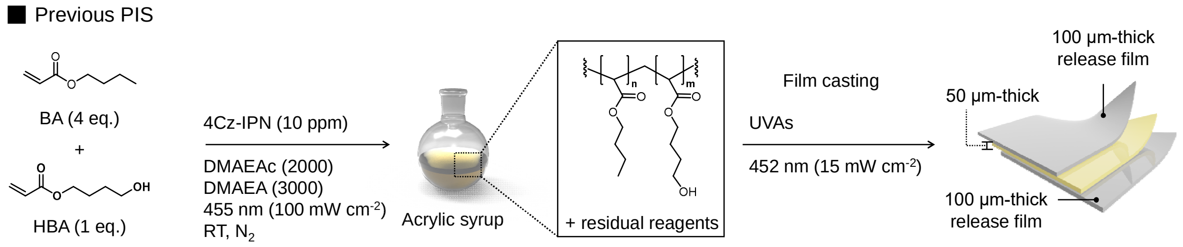

Entry	UVA (ppm)	Dosage $_{Acrylic\ syrup}$ (mJ cm^{-2})	Conversion $_{Acrylic\ syrup}$ (%)	Dosage $_{Film}$ (mJ cm^{-2})	Conversion $_{Film}$ (%)
1			15.3		95.3
2			15.0		95.3
3	-	3000	15.0	5400	95.8
4			15.3		94.7
5			15.0		95.4
6			15.3		96.1
7			15.0		95.4
8	UVA-1 (1500)<br>UVA-2 (4800)	3000	15.0	27000	94.6
9			15.3		97.3
10			15.0		91.2

Supplementary Table 11 Reproducibility test in the newly designed PIS (with [EHA]:[HBA] = 3:1) using 4DP-IPN (3 ppm), HNu 254 (1000 ppm) and Borate V (600 ppm). PIS reaction conditions were followed the general procedures to prepare acrylic syrup and film curing. All conversions were determined by FT-IR.

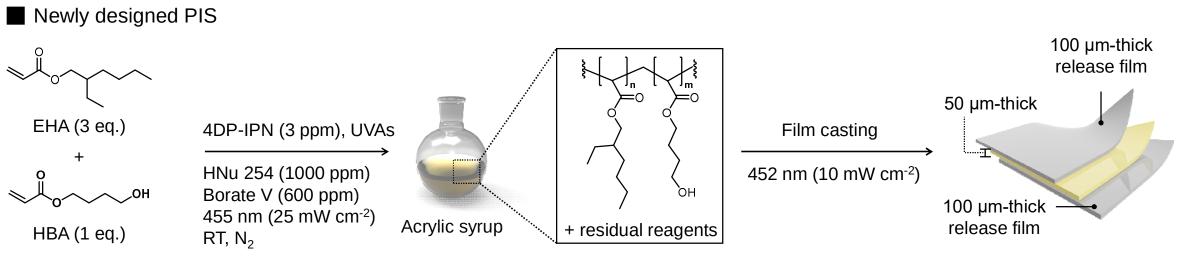

Entry	UVA (ppm)	Dosage $_{\text{Acrylic syrup}}$ (mJ cm^{-2})	Conversion $_{\text{Acrylic syrup}}$ (%)	Dosage $_{\text{Film}}$ (mJ cm^{-2})	Conversion $_{\text{Film}}$ (%)
1			13.6		98.4
2			13.7		96.3
3	-	50	12.9	600	96.8
4			13.1		97.5
5			14.2		99.8
6			8.3		97.8
7			7.3		94.6
8	UVA-1 (2000) UVA-2 (6400)	125	7.7	2400	100
9			8.1		98.4
10			7.6		99.9

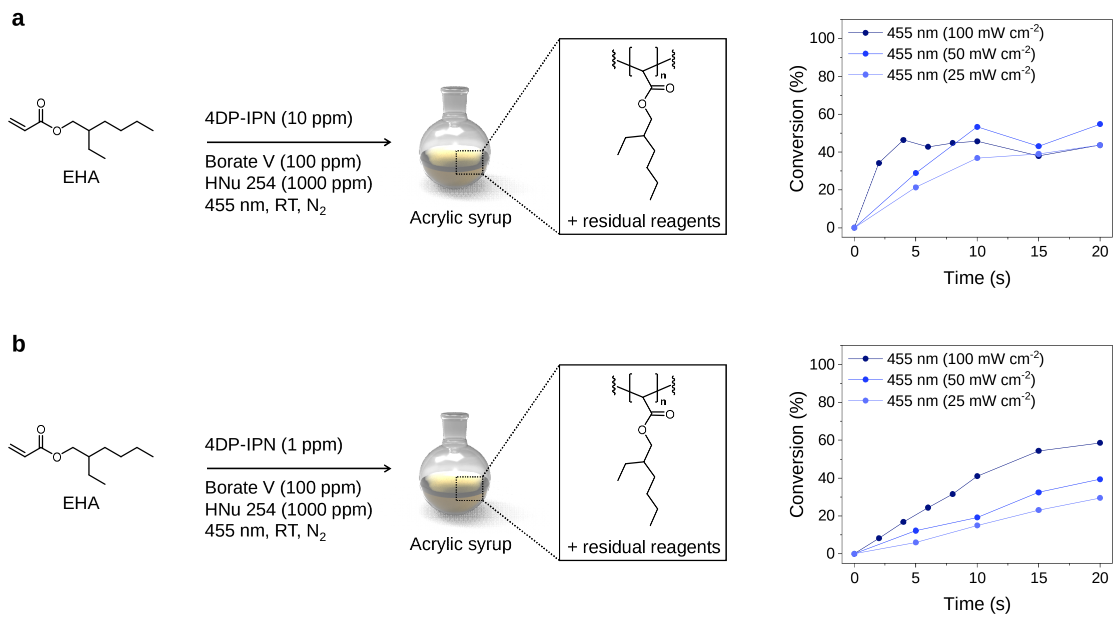

Supplementary Fig. 21 Conversion of acrylic syrup using 4DP-IPN, HNu254 (1000 ppm) and Borate V (100 ppm) with various light intensity (λ_{max} = 455 nm, 25–100 mW cm^{-2}). The polymerization of EHA was conducted with **a** 10 ppm (relative to monomer) and **b** 1 ppm of 4DP-IPN. All conversions were determined by FT-IR.

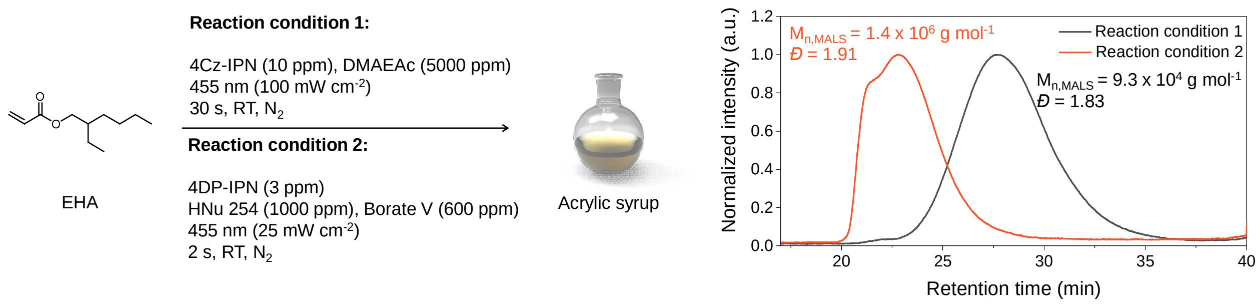

Supplementary Fig. 22 GPC traces of acrylic syrup of synthesized poly(EHA) with the previous PIS (Reaction condition 1, black line) and the newly designed PIS (Reaction condition 2, orange line) conditions. Molecular weights of the acrylic syrups are determined by MALS detector (dn/dc = 0.0702 for poly(EHA)).[29]

Supplementary Note 4. Characterization of UV-blocking OCA

4.1. Optical properties of UV-blocking OCA

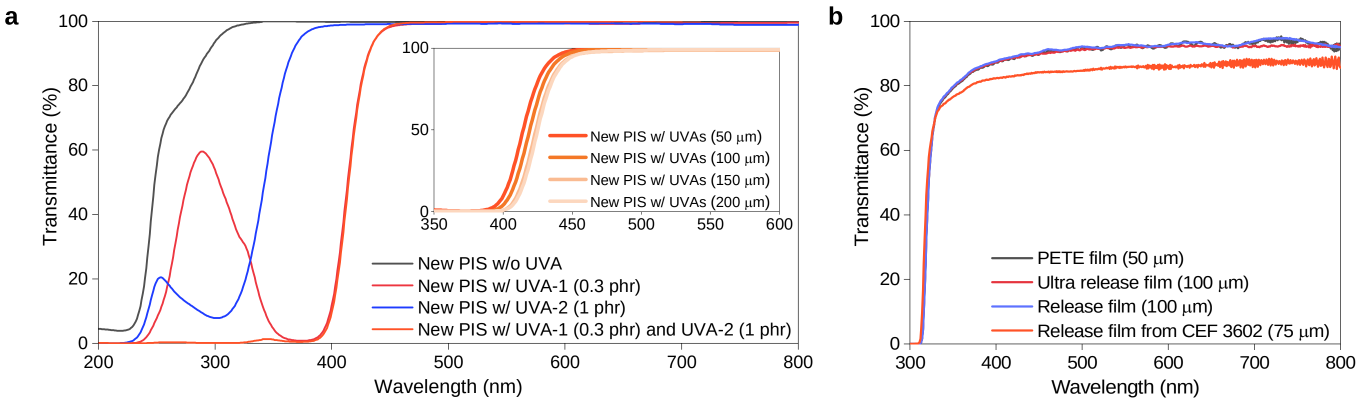

Supplementary Fig. 23 UV/vis transmission of **a** prepared OCAs synthesized following the general procedures and **b** films used in this work; PETE (50 μm, youngwoo trading), release film (silicon-treated PETE film, 100 μm, youngwoo trading), super release film (silicon-treated PETE film, 100 μm, youngwoo trading) and release film from CEF 3602 provided together to protect the OCA.

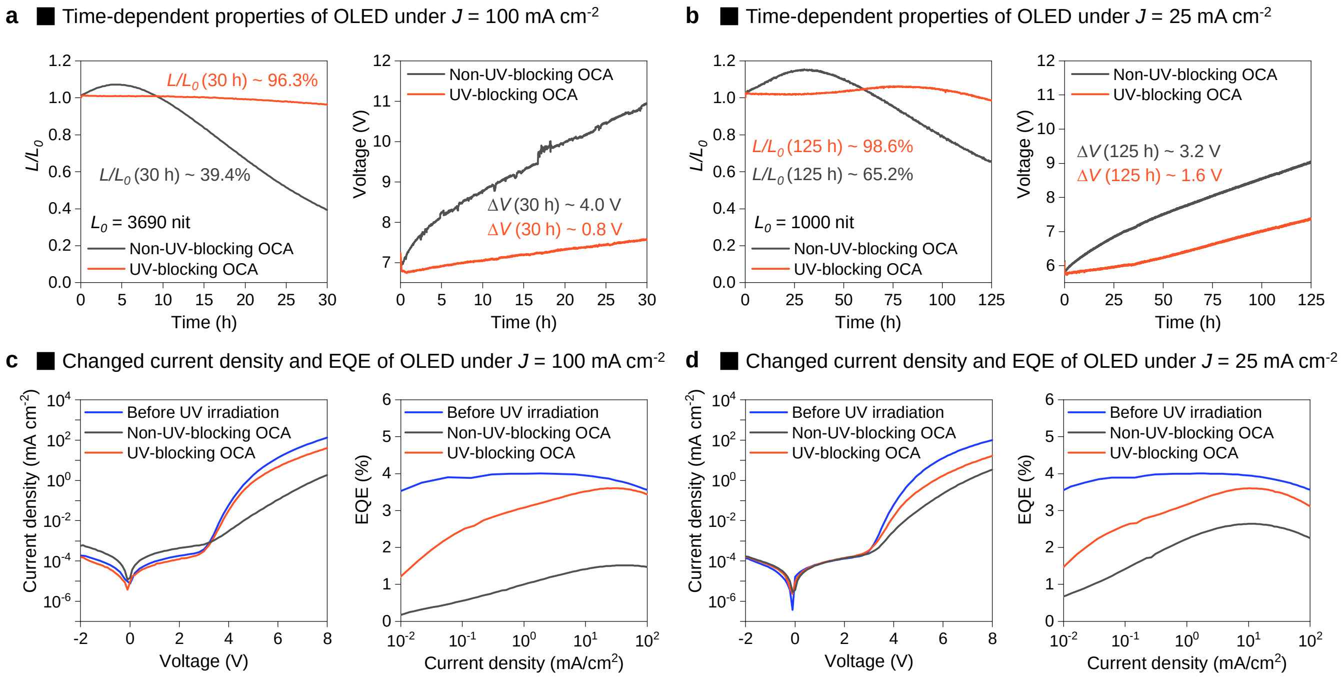

Supplementary Fig. 24 UV-blocking test of OLEDs covered by OCAs. **a, b** Time-dependent changes of luminance (left) and voltage (right) of blue OLED covered by the prepared non-UV-blocking OCA film (black line) and UV-blocking OCA film (orange line) exposed by UV-irradiation **a** under constant current (J = 100 mA cm^{-2}) and **b** under constant current (J = 25 mA cm^{-2}). **c, d** Change in current density (left) and external quantum efficiency (EQE) (right) of blue OLED covered by the prepared non-UV-blocking OCA film (black line) and UV-blocking OCA film (orange line) exposed by UV irradiation over voltage **c** under constant current (J = 100 mA cm^{-2}) and **d** under constant current (J = 25 mA cm^{-2}). As control experiment, non-UV-irradiated OLEDs were also monitored (blue line). All measurements in electronic properties of blue OLED were recorded every 10 min.

4.2. Mechanical properties of UV-blocking OCA

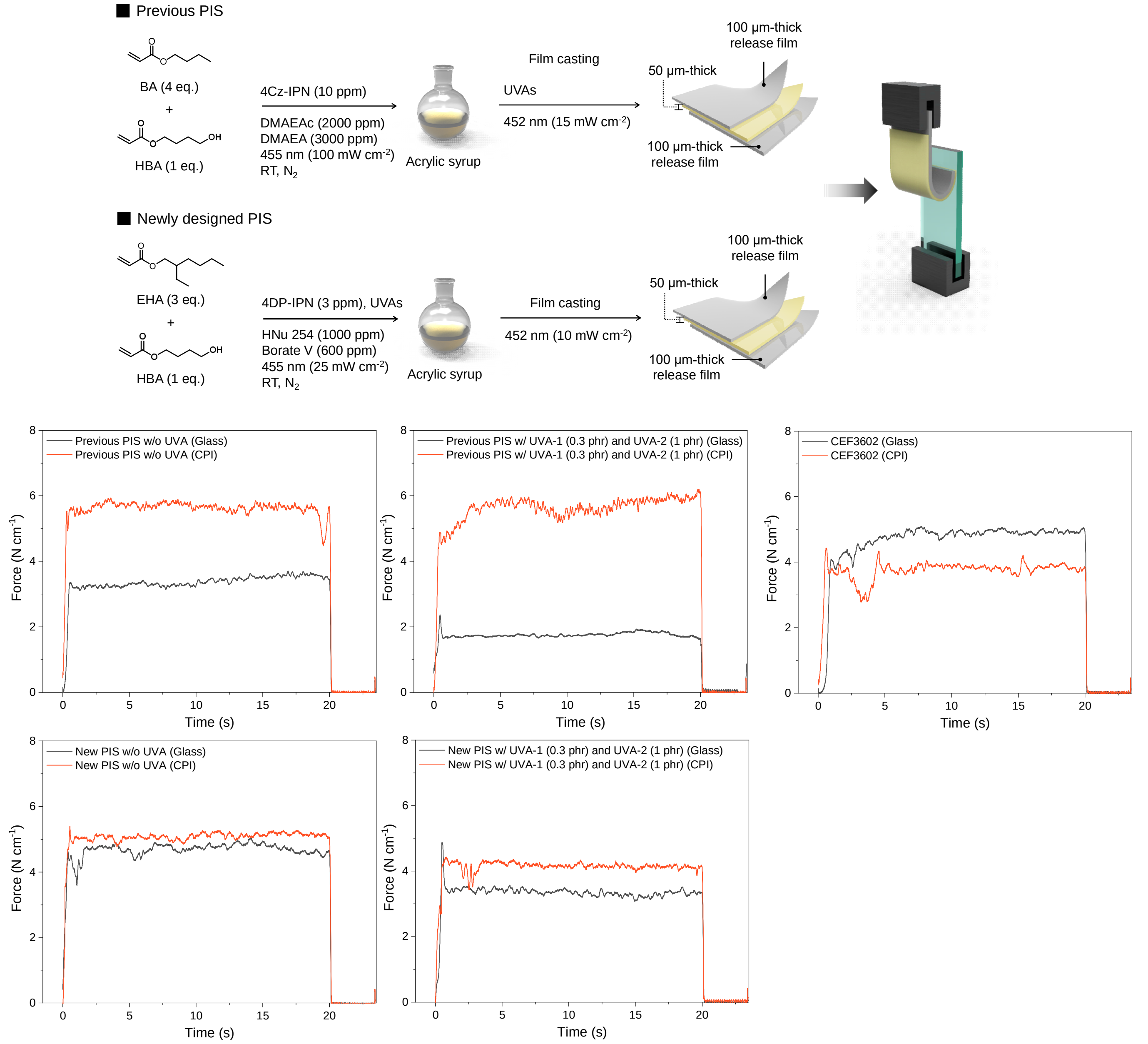

Supplementary Fig. 25 Results of peel strength of prepared OCA films with the previous PIS and the new PIS conditions. PIS reaction conditions were followed the general procedures to prepare acrylic syrup and film curing. The previous PIS (with [BA]:[HBA] = 4:1) were conducted with 4Cz-IPN (10 ppm), DMAEAc (2000 ppm), DMAEA (3000 ppm), UVA-1 (1500 ppm) and UVA-2 (4800 ppm). For the newly designed PIS (with [EHA]:[HBA] = 3:1), 4DP-IPN (3 ppm), HNu 254 (1000 ppm), Borate V (600 ppm), UVA-1 (2000 ppm) and UVA-2 (6400 ppm) were used. Peel strengths of prepared OCA films were evaluated as the average of the measured forces in the time range (t = 5–15 s, 5 mm s^{-1}). CEF3602 was measured as a control experiment.

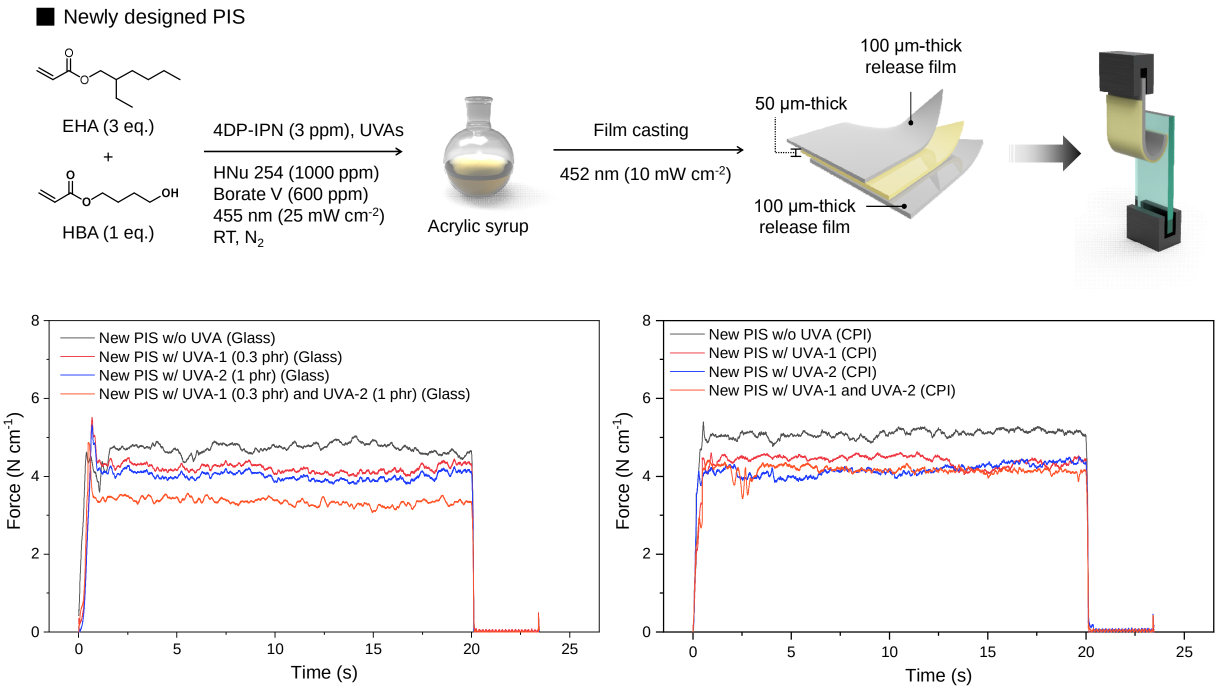

Supplementary Fig. 26 Results of peel strength of prepared OCA films with the newly designed PIS with glass (left part) and CPI (right part). For the newly designed PIS (with [EHA]:[HBA] = 3:1), 4DP-IPN (3 ppm), HNu 254 (1000 ppm), Borate V (600 ppm), UVA-1 (2000 ppm) and UVA-2 (6400 ppm) were used. Peel strengths of prepared OCA films were evaluated as the average of the measured forces in the time range (t = 5–15 s, 5 mm s^{-1}).

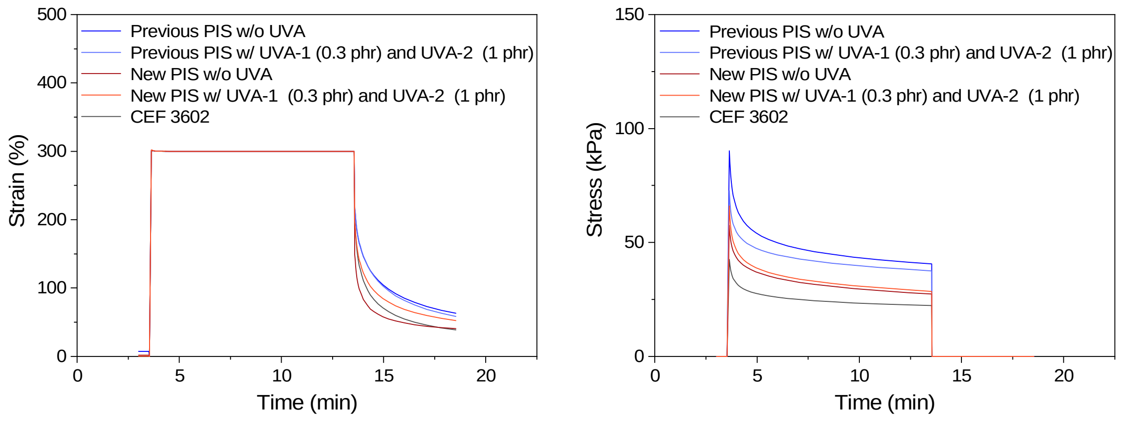

Supplementary Fig. 27 Results of stain recovery (left part) and stress relaxation (right part) of prepared OCA films with the previous PIS and the newly designed PIS conditions with or without UVA. The previous PIS (with [BA]:[HBA] = 4:1) were conducted with 4Cz-IPN (10 ppm), DMAEAc (2000 ppm), DMAEA (3000 ppm), UVA-1 (1500 ppm) and UVA-2 (4800 ppm). For the newly designed PIS (with [EHA]:[HBA] = 3:1), 4DP-IPN (3 ppm), HNu 254 (1000 ppm), Borate V (600 ppm), UVA-1 (2000 ppm) and UVA-2 (6400 ppm) were used. Strain recovery and stress relaxation of prepared OCAs were evaluated at 25 ℃. The measurements of strain recovery were carried out at 25 ℃ over 5 min, after the specimen was kept for 10 min at 300% strain,[6] with preload force set as 0.001 N. CEF3602 was measured as a control experiment.

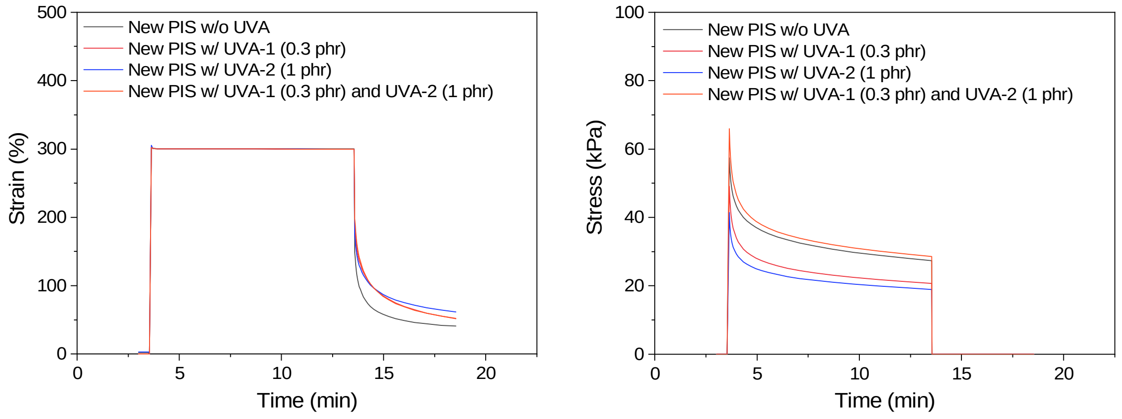

Supplementary Fig. 28 Results of stain recovery (left part) and stress relaxation (right part) of prepared OCA films with the newly designed PIS along with additions of UVAs. For the newly designed PIS (with [EHA]:[HBA] = 3:1), 4DP-IPN (3 ppm), HNu 254 (1000 ppm), Borate V (600 ppm), UVA-1 (2000 ppm) and UVA-2 (6400 ppm) were used. Strain recovery and stress relaxation of prepared OCAs were evaluated at 25 ℃. The measurements of strain recovery were carried out at 25 ℃ over 5 min, after the specimen was kept for 10 min at 300% strain,[6] with preload force set as 0.001 N.

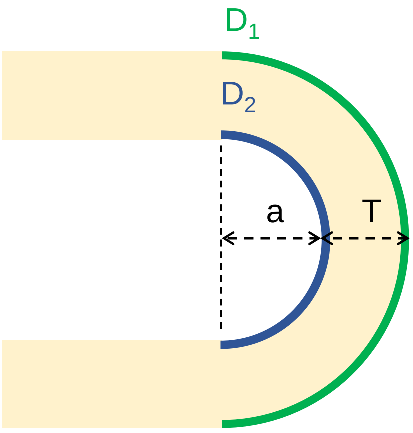

Supplementary Fig. 29 Schematic illustration of 180° folded OCA in the foldable display.

Applying a 300% strain is a common standard for testing the strain recovery of OCAs used in foldable displays.[6] This level of strain correlates with the curvature that these displays typically experience in practical applications (Supplementary Fig. 29); the shear behavior of an OCA can be illustrated by the difference in length between an inner curve (Supplementary Fig. 29, blue line) and an outer curve (Supplementary Fig. 29, green line), as represented by following equations (30−32).

$$D_1 = (a + T) \times \pi \quad (30)$$

$$D_2 = a \times \pi \quad (31)$$

$$\text{Shear strain} = \frac{D}{T} = \frac{D_1 - D_2}{T} = \frac{T \times \pi}{T} = \pi \quad (32)$$

In the given context, D_1 and D_2 represent the outer and inner folding curves, respectively, while D signifies a displacement in the shear strain. T corresponds to the thickness of the OCA, and a is the folding radius. It follows from the relationship in the shear strain that the displacement is likely to increase in proportion to the thickness, multiplied by π. Consequently, under the conditions of 180° folding, we applied a strain of approximately 300% in order to assess the strain recovery and stress relaxation.

Supplementary Table 12 Results of storage modulus (G'), loss modulus (G''), and damping factor (tan δ) of prepared OCA films with the previous PIS and newly designed PIS conditions obtained by dynamic temperature sweep. The previous PIS (with [BA]:[HBA] = 4:1) were conducted with 4Cz-IPN (10 ppm), DMAEAc (2000 ppm), DMAEA (3000 ppm), UVA-1 (1500 ppm) and UVA-2 (4800 ppm). For the newly designed PIS (with [EHA]:[HBA] = 3:1), 4DP-IPN (3 ppm), HNu 254 (1000 ppm), Borate V (600 ppm), UVA-1 (2000 ppm) and UVA-2 (6400 ppm) were used. The viscoelastic properties of prepared OCAs were evaluated at -20 °C, 25 °C, 60 °C, and 85 °C, respectively. CEF3602 was measured as a control experiment.

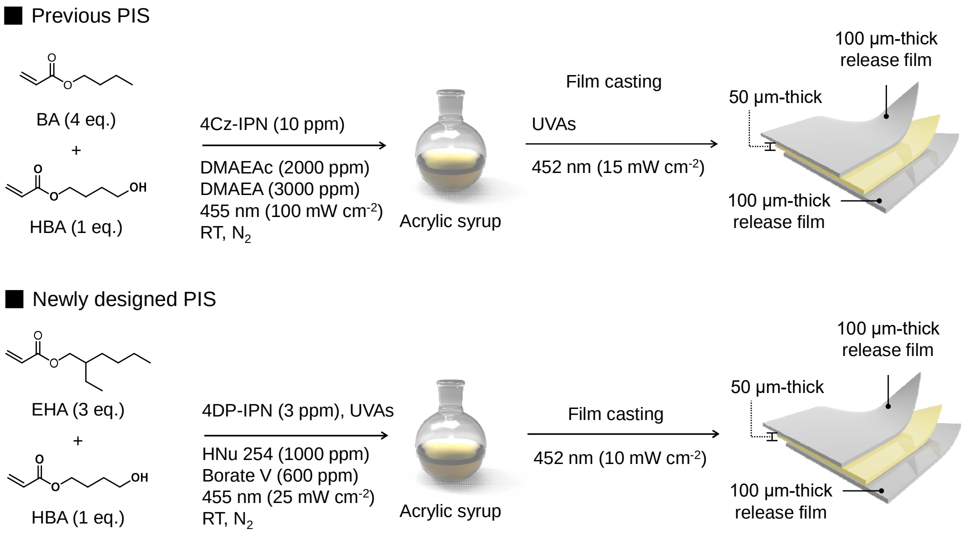

Entry	OCA	Storage modulus (G') (kPa)				Loss modulus (G'') (kPa)				Damping factor (tan δ)			
		–20 °C	25 °C	60 °C	85 °C	–20 °C	25 °C	60 °C	85 °C	–20 °C	25 °C	60 °C	85 °C
1	CEF 3602	115.0	45.3	32.3	27.6	78.8	12.4	10.0	8.7	0.69	0.27	0.31	0.32
2	Previous PIS w/o UVA	194.0	76.1	54.9	45.5	147.0	17.4	15.9	14.0	0.76	0.23	0.29	0.31
3	Previous PIS w/ UVAs	115.2	68.6	57.7	53.4	59.3	13.2	13.2	12.7	0.51	0.19	0.23	0.24
4	New PIS w/o UVA	145.0	41.2	28.0	22.8	134.6	13.4	11.1	10.0	0.93	0.33	0.40	0.44
5	New PIS w/ UVAs	150.3	45.0	36.1	32.7	145.4	11.1	8.8	8.1	0.97	0.25	0.24	0.25

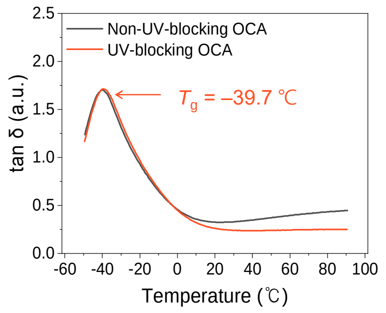

Supplementary Fig. 30 Results of damping factor (tan δ) of the prepared OCA film measured from rheometer with dynamic temperature sweep from -50 °C to 90 °C. For the preparation of OCAs (with [EHA]:[HBA] = 3:1), 4DP-IPN (3 ppm), HNu 254 (1000 ppm), and Borate V (600 ppm) were used. For the preparation of UV-blocking OCA, UVA-1 (2000 ppm) and UVA-2 (6400 ppm) were added.

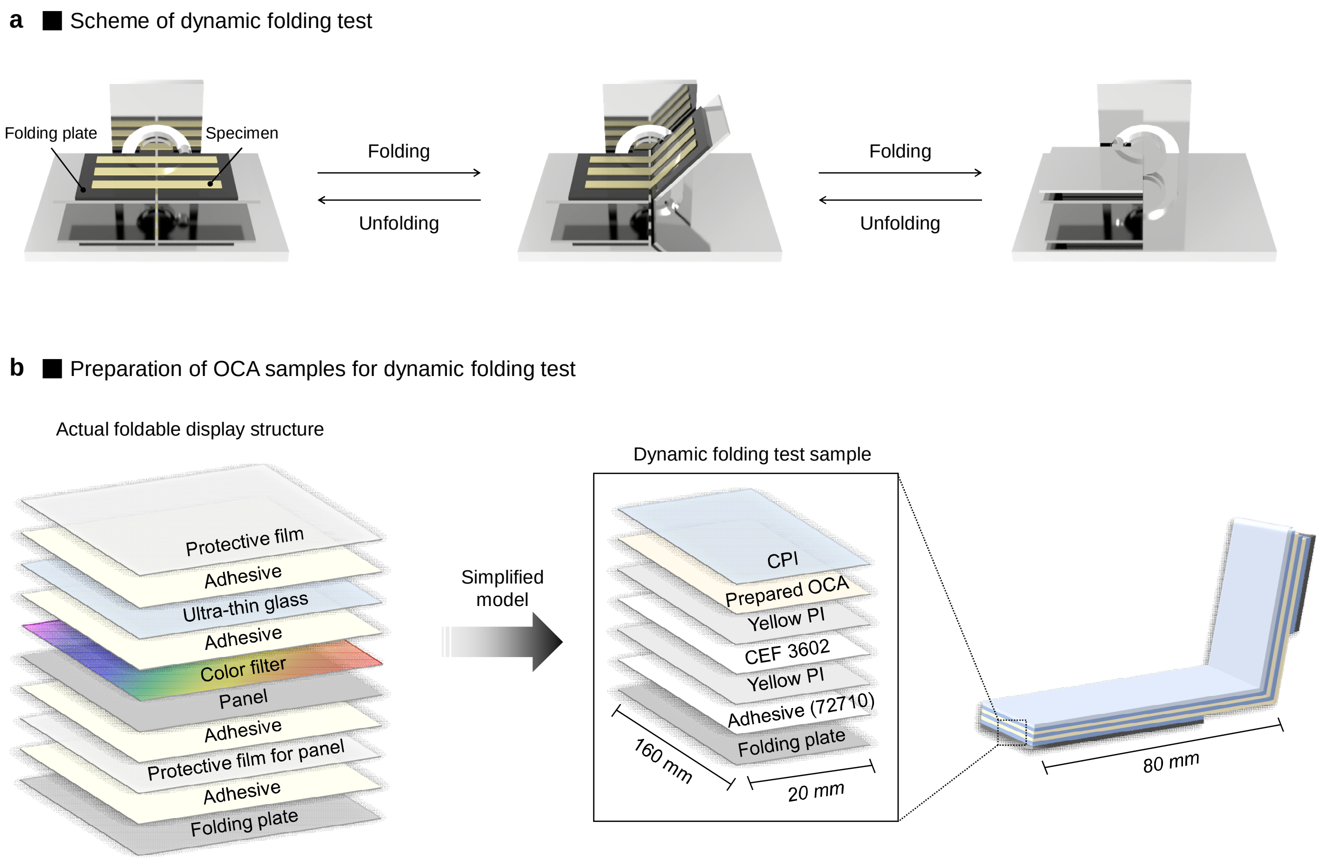

Supplementary Fig. 31 Dynamic folding test of prepared OCAs. **a** Schematic illustration of dynamic folding test. **b** Preparation of OCA samples for dynamic folding test. The OCA samples were prepared by stacking colorless polyimide (CPI, Kolon Industries, 50 μm), prepared OCA (50 μm), yellow PI (GL140A, 35 μm), CEF 3602 (3M, 50 μm), and yellow PI (GL140A, 35 μm) in this order. After preparing the sample, commercial acrylic adhesive (3M, 72710) was used as an adhesive layer to attach to the folding plate.

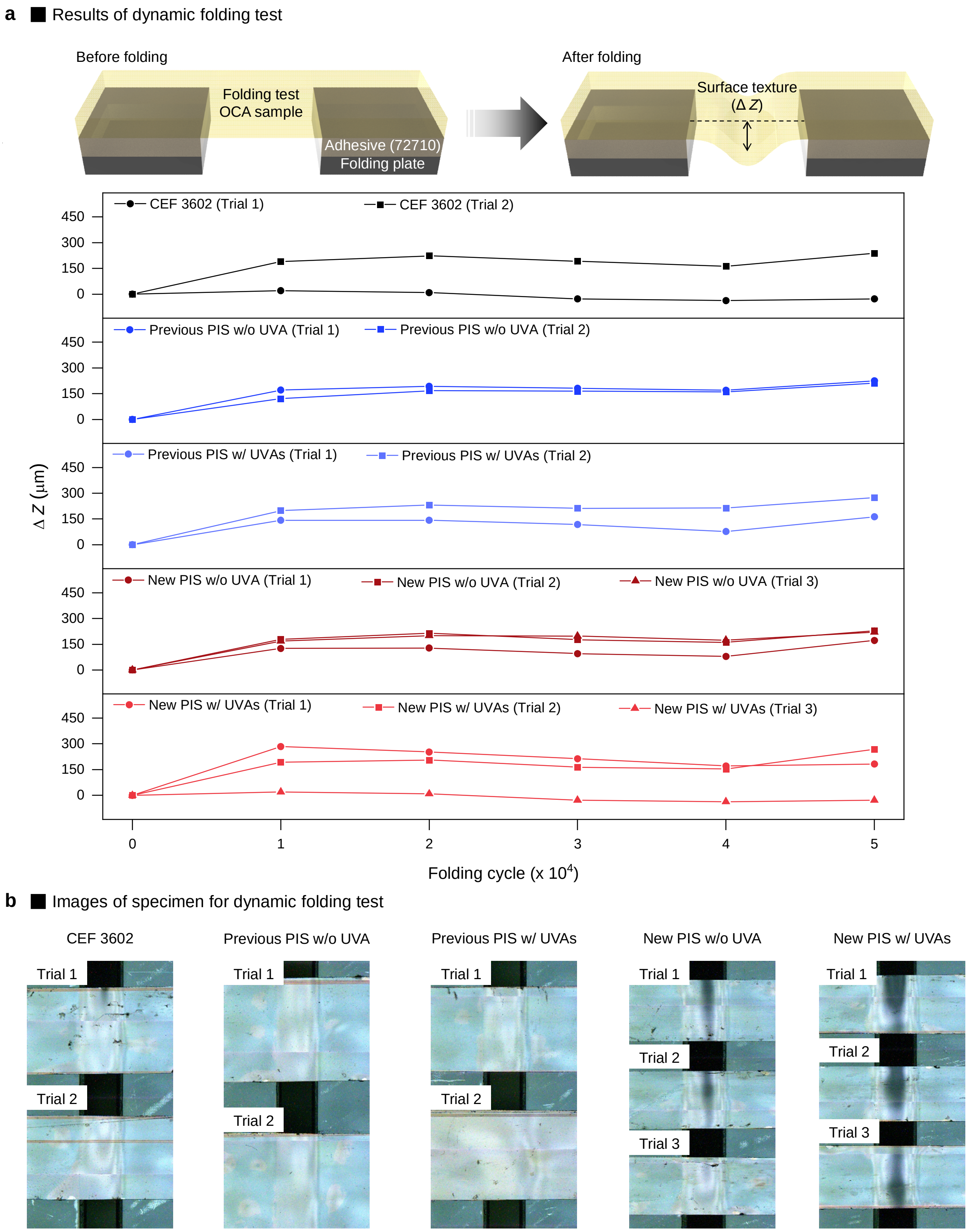

Supplementary Fig. 32 Results of dynamic folding test of prepared OCAs. **a** Surface texture (ΔZ) values after the dynamic folding test. The values of ΔZ of each OCA sample were measured after folds by 5 x 10^4 times at -20 °C. CEF 3602 and previous PIS OCAs were tested with two identical samples, and new PIS OCAs were measured three identical samples. The OCA samples were cured by following conditions: the previous PIS (with [BA]:[HBA] = 4:1) were conducted with 4Cz-IPN (10 ppm), DMAEAc (2000 ppm), DMAEA (3000 ppm), UVA-1 (1500 ppm) and UVA-2 (4800 ppm) and the newly designed PIS (with [EHA]:[HBA] = 3:1) were conducted with 4DP-IPN (3 ppm), HNu 254 (1000 ppm), Borate V (600 ppm), UVA-1 (2000 ppm) and UVA-2 (6400 ppm) were used. Error bars represent the standard deviation. **b** Images of specimen for dynamic folding test. No defect or delamination in the tested OCA samples were observed at -20 °C.

Supplementary Note 5. Coordinates of molecular structures obtained by DFT calculation

The geometries optimizations of each species were obtained DFT calculations were performed with the B3LYP functional and 6–311++G* basis set in ethyl acetate solution employing the PCM solvation model as all implemented in the Gaussian16 program package. In all geometry optimization calculations, the frequency calculations were performed both to verify that the geometries were true minima and to obtain thermochemistry-correction values.

■ 4Cz-IPN (neutral)

Electronic energy = -2482.329703 (hartree)

Electronic energy + zero-point energy = -2481.610451 (hartree)

Electronic energy + thermal energy correction = -2481.564656 (hartree)

Electronic energy + thermal enthalpy correction = -2481.563711 (hartree)

Electronic energy + thermal free energy correction = -2481.691857 (hartree)

C	-0.08420600	1.19635600	0.25000800
C	-1.49644800	1.17540600	0.27507100
C	-2.20758800	0.00014000	0.00009300
C	-1.49653700	-1.17520100	-0.27470600
C	-0.08429500	-1.19624400	-0.24958500
C	0.63227400	0.00004000	0.00009800
C	-2.22612600	2.33811800	0.67578200
C	-2.22629700	-2.33792600	-0.67522500
N	-2.82812000	-3.25821200	-1.02641500
N	-2.82788100	3.25839000	1.02712300
N	-3.61951500	0.00019700	-0.00001700
C	-4.43821900	-0.44288500	1.05133000
C	-4.43792800	0.44357000	-1.05145100
C	-4.07713900	-0.94625600	2.29921000
C	-5.78996700	-0.28013300	0.66818900
C	-5.78978400	0.28139600	-0.66845200
C	-4.07648400	0.94669400	-2.29933000
C	-5.10369200	-1.30351700	3.17030300
H	-3.03896600	-1.05398500	2.59340800
C	-6.80153900	-0.64493400	1.56231000
C	-6.80109800	0.64658100	-1.56271000
C	-5.10278600	1.30434800	-3.17055600
H	-3.03822000	1.05389000	-2.59341300
C	-6.45174200	-1.15716900	2.80769300
H	-4.85296500	-1.69946200	4.14876600
H	-7.84564800	-0.52820900	1.29079800

C	-6.45093900	1.15860700	-2.80807800
H	-7.84528900	0.53029100	-1.29132600
H	-4.85178800	1.70012100	-4.14901900
H	-7.22765300	-1.44371900	3.50940800
H	-7.22665100	1.44545200	-3.50989100
N	0.57331600	-2.41352100	-0.51183100
C	1.39186600	-2.69986600	-1.62288100
C	0.47574200	-3.58006900	0.27667500
C	1.70087800	-1.90543800	-2.72349300
C	1.84308400	-4.03519000	-1.52000000
C	1.26734000	-4.59081400	-0.31229000
C	-0.22199600	-3.79544300	1.46335600
C	2.51184000	-2.45580400	-3.71309800
H	1.32562000	-0.89526900	-2.82321500
C	2.65617500	-4.56687900	-2.52513100
C	1.35872800	-5.84213100	0.30416300
C	-0.11992200	-5.05109200	2.05740100
H	-0.83538700	-3.02672800	1.91874700
C	2.99295300	-3.76982200	-3.61426300
H	2.77071500	-1.85383800	-4.57759100
H	3.01027900	-5.59067000	-2.46254500
C	0.66237700	-6.06620900	1.48714900
H	1.96511700	-6.62811100	-0.13398800
H	-0.66094300	-5.24425100	2.97760900
H	3.62275200	-4.16991500	-4.40159500
H	0.72143800	-7.03381500	1.97369000
N	2.04355900	0.00009600	-0.00020300
C	2.87136800	-0.70965200	0.89345500
C	2.87077800	0.70974800	-0.89447500
C	2.52743700	-1.50889700	1.98093200
C	4.22122000	-0.44867200	0.56709700
C	4.22084400	0.44858900	-0.56916100
C	2.52609100	1.50908700	-1.98164000
C	3.55907700	-2.07211400	2.72788300
H	1.49666500	-1.69512200	2.25381200
C	5.24133700	-1.02336900	1.33094900
C	5.24043700	1.02318200	-1.33378800
C	3.55722500	2.07219600	-2.72938000
H	1.49512200	1.69544600	-2.25370000

C	4.90427600	-1.83819000	2.40640600
H	3.31212700	-2.70347600	3.57482900
H	6.28229800	-0.83196700	1.09143700
C	4.90264100	1.83807900	-2.40896200
H	6.28156000	0.83165000	-1.09508600
H	3.30969600	2.70361600	-3.57611300
H	5.68558400	-2.29236300	3.00636800
H	5.68354200	2.29217400	-3.00951400
N	0.57350300	2.41349400	0.51264500
C	1.39292200	2.69902100	1.62326700
C	0.47624200	3.58033500	-0.27550300
C	1.70193700	1.90414000	2.72355100
C	1.84498200	4.03406000	1.52046600
C	1.26889400	4.59037900	0.31324200
C	-0.22218300	3.79656100	-1.46161300
C	2.51381800	2.45370600	3.71284500
H	1.32595000	0.89424400	2.82327300
C	2.65896300	4.56496200	2.52529500
C	1.36073600	5.84181300	-0.30290200
C	-0.11963600	5.05231300	-2.05536800
H	-0.83650700	3.02845300	-1.91674300
C	2.99578600	3.76741400	3.61405200
H	2.77272800	1.85136600	4.57706700
H	3.01370500	5.58853700	2.46278300
C	0.66376400	6.06671700	-1.48536600
H	1.96793800	6.62725100	0.13509400
H	-0.66118700	5.24612200	-2.97512800
H	3.62627800	4.16689400	4.40114100
H	0.72315600	7.03442800	-1.97165800

■ 4Cz-IPN (radical anion)

Electronic energy = -2482.443823 (hartree)

Electronic energy + zero-point energy = -2481.728293 (hartree)

Electronic energy + thermal energy correction = -2481.682071 (hartree)

Electronic energy + thermal enthalpy correction = -2481.681126 (hartree)

Electronic energy + thermal free energy correction = -2481.811736 (hartree)

C	-0.03191400	-1.20095400	-0.23462500
C	-1.49194600	-1.18653400	-0.28134500

C	-2.17802500	0.00003100	0.00000600
C	-1.49190700	1.18657500	0.28135200
C	-0.03187600	1.20094600	0.23463800
C	0.65826800	-0.00001500	0.00001000
C	-2.21240800	-2.34158600	-0.65258800
C	-2.21233300	2.34165400	0.65258300
N	-2.79939600	3.29732900	0.96204000
N	-2.79958700	-3.29720800	-0.96198600
N	-3.60353600	0.00005300	0.00000400
C	-4.41791200	0.40317700	-1.05976000
C	-4.41793000	-0.40305700	1.05976100
C	-4.05526100	0.86997900	-2.32312700
C	-5.77439900	0.25647100	-0.67735100
C	-5.77441100	-0.25632500	0.67733900
C	-4.05530000	-0.86986400	2.32313200
C	-5.07788400	1.19820100	-3.20863500
H	-3.01445700	0.97444400	-2.60826100
C	-6.78286300	0.59319600	-1.58670700
C	-6.78288900	-0.59302900	1.58668500
C	-5.07793700	-1.19806600	3.20863000
H	-3.01450100	-0.97435000	2.60827500
C	-6.42909600	1.06325500	-2.84750700
H	-4.82359000	1.56490000	-4.19794600
H	-7.82844000	0.48793200	-1.31424500
C	-6.42914300	-1.06309400	2.84748900
H	-7.82846100	-0.48774600	1.31421400
H	-4.82366000	-1.56477000	4.19794400
H	-7.20241000	1.32762600	-3.56126800
H	-7.20246900	-1.32744900	3.56124300
N	0.63253700	2.44085200	0.43127300
C	1.28116600	2.86780600	1.59586100
C	0.60864400	3.51469800	-0.46611100
C	1.48075200	2.18710200	2.79646300
C	1.72805100	4.20131600	1.41908100
C	1.29547900	4.61538000	0.10135900
C	0.02002100	3.59516100	-1.72874100
C	2.16022900	2.84810600	3.81558300
H	1.11840600	1.17640800	2.93548400
C	2.41399600	4.84316700	2.45573800

C	1.41093200	5.80589500	-0.62422100
C	0.14192200	4.79162800	-2.42894400
H	-0.52539400	2.75857600	-2.15026300
C	2.63079500	4.16140800	3.64882400
H	2.32784100	2.33618400	4.75786800
H	2.76490600	5.86357400	2.33520100
C	0.83504600	5.88769000	-1.88793800
H	1.93747800	6.65802200	-0.20531100
H	-0.31208500	4.87893100	-3.41100600
H	3.16028600	4.64833200	4.46134100
H	0.91265300	6.80720800	-2.45895000
N	2.08817100	-0.00003000	0.00000200
C	2.90861600	0.55276100	-0.98918600
C	2.90862500	-0.55281600	0.98918700
C	2.56079500	1.18995400	-2.18012200
C	4.26482100	0.35144100	-0.63182700
C	4.26482600	-0.35153200	0.63179500
C	2.56081500	-1.18998300	2.18014000
C	3.58688200	1.63784500	-3.00607100
H	1.52570400	1.34186800	-2.45694800
C	5.27993300	0.80968500	-1.47839700
C	5.27994700	-0.80979400	1.47834600
C	3.58690900	-1.63789100	3.00607000
H	1.52572700	-1.34186800	2.45699100
C	4.93626300	1.45327900	-2.66223600
H	3.33568300	2.14145800	-3.93405400
H	6.32311700	0.66252700	-1.21602200
C	4.93628700	-1.45336700	2.66219900
H	6.32312800	-0.66266400	1.21594600
H	3.33571900	-2.14148800	3.93406400
H	5.71369700	1.81555300	-3.32697000
H	5.71372700	-1.81565400	3.32691900
N	0.63245500	-2.44088400	-0.43126500
C	1.28107600	-2.86785500	-1.59585200
C	0.60851800	-3.51473500	0.46611200
C	1.48068900	-2.18715500	-2.79645100
C	1.72792200	-4.20137800	-1.41907200
C	1.29532600	-4.61543400	-0.10135500
C	0.01987600	-3.59518700	1.72873500

C	2.16015400	-2.84817600	-3.81556800
H	1.11837300	-1.17645000	-2.93547200
C	2.41385600	-4.84324600	-2.45572500
C	1.41073900	-5.80595400	0.62422200
C	0.14173500	-4.79166000	2.42893400
H	-0.52552100	-2.75858800	2.15025400
C	2.63068100	-4.16149100	-3.64880900
H	2.32778600	-2.33625700	-4.75785000
H	2.76473400	-5.86366500	-2.33518800
C	0.83483700	-5.88773900	1.88793200
H	1.93726400	-6.65809500	0.20531300
H	-0.31228400	-4.87895400	3.41099000
H	3.16016300	-4.64842900	-4.46132400
H	0.91241200	-6.80726100	2.45894200

■ 4Cz-IPN (radical cation)

Electronic energy = -2482.108894 (hartree)

Electronic energy + zero-point energy = -2481.389959 (hartree)

Electronic energy + thermal energy correction = -2481.344382 (hartree)

Electronic energy + thermal enthalpy correction = -2481.343438 (hartree)

Electronic energy + thermal free energy correction = -2481.469748 (hartree)

C	-0.13366500	-1.19301900	-0.26009200
C	-1.54331300	-1.18391200	-0.26412900
C	-2.25681700	-0.00056200	0.00005600
C	-1.54374500	1.18303700	0.26429900
C	-0.13409100	1.19275000	0.26027800
C	0.57408600	-0.00001900	0.00010500
C	-2.25683800	-2.35499700	-0.66528000
C	-2.25774900	2.35379400	0.66557500
N	-2.83341300	3.29154400	1.01331100
N	-2.83209500	-3.29305900	-1.01284600
N	-3.66008400	-0.00080500	-0.00003000
C	-4.48160300	0.61961400	-0.96154100
C	-4.48157700	-0.62108300	0.96159400
C	-4.12107500	1.30991300	-2.11628300
C	-5.83136300	0.39133000	-0.61088600
C	-5.83134600	-0.39271200	0.61102700
C	-4.12101600	-1.31140000	2.11631000

C	-5.14873500	1.80399100	-2.91685200
H	-3.08487500	1.45691700	-2.39791300
C	-6.84332500	0.89230600	-1.43130300
C	-6.84328400	-0.89363700	1.43150400
C	-5.14865300	-1.80541000	2.91695100
H	-3.08480500	-1.45845400	2.39786800
C	-6.49420800	1.60213500	-2.57851400
H	-4.89942800	2.34965700	-3.82029100
H	-7.88676700	0.72779900	-1.18488200
C	-6.49413600	-1.60348500	2.57869500
H	-7.88673100	-0.72906100	1.18515300
H	-4.89931900	-2.35108700	3.82037600
H	-7.27176100	1.99722600	-3.22280800
H	-7.27167100	-1.99852600	3.22304000
N	0.55460200	2.37799600	0.58548000
C	1.34873000	2.58129700	1.73746900
C	0.57007700	3.55541400	-0.17915700
C	1.53484600	1.75082000	2.83774700
C	1.90714200	3.87609800	1.67397300
C	1.41870700	4.49493900	0.45625900
C	-0.07745200	3.84209800	-1.38286900
C	2.34664100	2.22041000	3.87166100
H	1.05752800	0.78239900	2.91503800
C	2.71332500	4.32688200	2.71949000
C	1.61879800	5.74369000	-0.13030400
C	0.13406400	5.09557600	-1.94575100
H	-0.73485100	3.13061000	-1.86723800
C	2.93668100	3.48759900	3.81140500
H	2.51158300	1.59153800	4.73932900
H	3.14990100	5.31937800	2.69302800
C	0.97292000	6.03751100	-1.32958300
H	2.26508100	6.47770400	0.33806200
H	-0.36371000	5.34897400	-2.87499300
H	3.55969900	3.82744000	4.63108100
H	1.11649800	7.00715300	-1.79295100
N	1.99171400	0.00017700	0.00003500
C	2.81214400	0.70608800	-0.89674900
C	2.81246900	-0.70534800	0.89682900
C	2.45438500	1.48959000	-1.99482700

C	4.16474600	0.44898000	-0.57173900
C	4.16495000	-0.44764500	0.57179700
C	2.45509700	-1.48901300	1.99491400
C	3.48079500	2.05452000	-2.74918200
H	1.42191600	1.65472800	-2.27127400
C	5.17359400	1.01797000	-1.34065000
C	5.17407100	-1.01618700	1.34068100
C	3.48177700	-2.05348400	2.74925000
H	1.42271600	-1.65459000	2.27142200
C	4.82278900	1.82903100	-2.42518300
H	3.23009000	2.67404900	-3.60245200
H	6.21751400	0.83371700	-1.11279200
C	4.82366300	-1.82740600	2.42522400
H	6.21790000	-0.83147200	1.11278200
H	3.23136100	-2.67311200	3.60253300
H	5.60185600	2.27909400	-3.02991200
H	5.60294300	-2.27712100	3.02993500
N	0.55553000	-2.37797800	-0.58543500
C	1.34940700	-2.58089500	-1.73765600
C	0.57177900	-3.55533100	0.17919500
C	1.53478700	-1.75031800	-2.83797600
C	1.90848400	-3.87542100	-1.67429800
C	1.42068500	-4.49448900	-0.45643600
C	-0.07535000	-3.84225400	1.38308600
C	2.34652700	-2.21950300	-3.87212700
H	1.05693300	-0.78215000	-2.91512900
C	2.71458200	-4.32579700	-2.72004800
C	1.62145900	-5.74312100	0.13013000
C	0.13683600	-5.09561700	1.94593800
H	-0.73287500	-3.13095700	1.86756700
C	2.93720900	-3.48638900	-3.81203200
H	2.51092300	-1.59055300	-4.73984200
H	3.15166400	-5.31807200	-2.69373600
C	0.97596900	-6.03718400	1.32957000
H	2.26796000	-6.47688800	-0.33832200
H	-0.36058200	-5.34924100	2.87530700
H	3.56016100	-3.82592700	-4.63188300
H	1.12008700	-7.00675700	1.79291500

■ 4DP-IPN (neutral)

Electronic energy = -2487.048336 (hartree)

Electronic energy + zero-point energy = -2486.244362 (hartree)

Electronic energy + thermal energy correction = -2486.193870 (hartree)

Electronic energy + thermal enthalpy correction = -2486.192925 (hartree)

Electronic energy + thermal free energy correction = -2486.333901 (hartree)

C	0.06399300	-1.21863800	0.12013700
C	-1.35122000	-1.19790200	0.12861300
C	-2.07383600	-0.00015600	-0.00022900
C	-1.35135400	1.19766500	-0.12908300
C	0.06385300	1.21853400	-0.12041700
C	0.78799200	-0.00000800	-0.00008300
C	-2.08481000	2.41071400	-0.30888600
C	-2.08456900	-2.41105000	0.30821100
N	-2.69813600	3.37645900	-0.46868500
N	-2.69775200	-3.37692300	0.46778500
N	-3.49250200	-0.00025200	-0.00020100
C	-4.18325200	-0.30857600	1.20832600
C	-4.18343700	0.30817500	-1.20856000
C	-5.36825600	-1.05641500	1.18521400
C	-3.67379000	0.12306200	2.43960200
C	-3.67382000	-0.12277900	-2.44001700
C	-5.36878800	1.05546600	-1.18515800
C	-6.03145100	-1.35237800	2.37140700
H	-5.76273600	-1.41164700	0.24079900
C	-4.33417100	-0.19614200	3.62496000
H	-2.76711500	0.71683600	2.47385800
C	-4.33438100	0.19654800	-3.62524100
H	-2.76686600	-0.71610900	-2.47452100
C	-6.03214500	1.35155100	-2.37122800
H	-5.76342000	1.41018600	-0.24061500
C	-5.51861800	-0.92947800	3.59923500
H	-6.94653900	-1.93471900	2.33575900
H	-3.92536000	0.14718000	4.56984800
C	-5.51916100	0.92933300	-3.59922700
H	-3.92542600	-0.14623900	-4.57026100
H	-6.94749900	1.93346100	-2.33533800
H	-6.03573900	-1.17018900	4.52185700

H	-6.03641800	1.17014000	-4.52174800
N	0.73344700	-2.46195900	0.26293400
C	1.38983900	-2.73344900	1.50858900
C	0.47132500	-3.51985000	-0.65689200
C	2.63975300	-3.36246800	1.52565100
C	0.77024400	-2.41010300	2.72076000
C	0.15373900	-3.22973100	-1.99144500
C	0.51487000	-4.86264400	-0.25426800
C	3.25874300	-3.65570400	2.73682800
H	3.11762100	-3.62495700	0.59027800
C	1.40159100	-2.69375800	3.93115100
H	-0.21116100	-1.94898800	2.72382800
C	-0.11428600	-4.25420300	-2.89478600
H	0.11965200	-2.20160800	-2.32851100
C	0.26219400	-5.88049800	-1.16947700
H	0.73172000	-5.11564300	0.77545900
C	2.64696300	-3.31780400	3.94551200
H	4.22815500	-4.14344300	2.73570300
H	0.90795700	-2.43793100	4.86298900
C	-0.05599000	-5.58830600	-2.49529800
H	-0.35887100	-4.00284400	-3.92197900
H	0.29873300	-6.91173300	-0.83286500
H	3.13485600	-3.54470800	4.88746100
H	-0.25817900	-6.38527300	-3.20272200
N	2.20780000	0.00011900	0.00004800
C	2.92358700	0.69029800	1.02521100
C	2.92392700	-0.68996000	-1.02496200
C	4.12233100	1.35820800	0.73343600
C	2.45909300	0.69293900	2.34491000
C	2.45963100	-0.69279600	-2.34472300
C	4.12281100	-1.35752700	-0.73297200
C	4.83240300	2.00520200	1.73958900
H	4.49997100	1.36688700	-0.28099500
C	3.16669800	1.35629600	3.34525200
H	1.55446700	0.15566300	2.59824000
C	3.16756700	-1.35601400	-3.34492900
H	1.55489700	-0.15578700	-2.59822600
C	4.83320100	-2.00438900	-1.73898100
H	4.50029700	-1.36605100	0.28151800

C	4.35918000	2.01384400	3.05272800
H	5.75778700	2.51529600	1.49105900
H	2.78670700	1.34286100	4.36178600
C	4.36017800	-2.01322600	-3.05219500
H	2.78772200	-1.34272300	-4.36151900
H	5.75868100	-2.51422900	-1.49028700
H	4.91401200	2.52218600	3.83407700
H	4.91527000	-2.52145500	-3.83343300
N	0.73320200	2.46192000	-0.26307200
C	0.47075300	3.51974000	0.65678400
C	1.38990400	2.73354200	-1.50850300
C	0.51402500	4.86254800	0.25419500
C	0.15312200	3.22949000	1.99129000
C	0.77083000	2.40990300	-2.72086700
C	2.63961800	3.36297900	-1.52521100
C	0.26107100	5.88031600	1.16942000
H	0.73088900	5.11562100	-0.77551200
C	-0.11518700	4.25387700	2.89465100
H	0.11922200	2.20134700	2.32832400
C	1.40247900	2.69368700	-3.93106700
H	-0.21041100	1.94844000	-2.72423300
C	3.25890300	3.65634500	-2.73620600
H	3.11711600	3.62569700	-0.58971400
C	-0.05713700	5.58800400	2.49521400
H	0.29740000	6.91157100	0.83284900
H	-0.35979100	4.00242300	3.92181600
C	2.64764200	3.31816000	-3.94507300
H	0.90923700	2.43761300	-4.86304600
H	4.22815200	4.14441100	-2.73477800
H	-0.25953200	6.38490900	3.20265000
H	3.13576000	3.54516800	-4.88687800

■ **4DP-IPN (radical anion)**

Electronic energy = -2487.147799 (hartree)

Electronic energy + zero-point energy = -2486.347117 (hartree)

Electronic energy + thermal energy correction = -2486.296488 (hartree)

Electronic energy + thermal enthalpy correction = -2486.295544 (hartree)

Electronic energy + thermal free energy correction = -2486.436694 (hartree)

C	0.06116400	-1.22058800	0.10322900
C	-1.39967000	-1.20687200	0.08309700
C	-2.09093000	0.00896800	-0.00279800
C	-1.39298200	1.22115100	-0.09083400
C	0.06828400	1.22503500	-0.10876300
C	0.76045700	0.00047100	0.00034700
C	-2.12056400	2.42663100	-0.18550100
C	-2.13450200	-2.40832900	0.17437500
N	-2.71965900	3.42110200	-0.27529100
N	-2.73927700	-3.39946200	0.26134200
N	-3.52519000	0.01131900	0.00048000
C	-4.19975000	-0.23434200	1.22437200
C	-4.21062600	0.25431800	-1.21571000
C	-5.40136700	-0.95901500	1.26040000
C	-3.65429800	0.22344100	2.43301800
C	-3.64967000	-0.15306400	-2.43588700
C	-5.44294400	0.92723700	-1.23532700
C	-6.04470400	-1.19701100	2.47097000
H	-5.82419400	-1.34479400	0.34073700
C	-4.29609100	-0.03536400	3.64215600
H	-2.73009000	0.78958400	2.42269300
C	-4.30527100	0.10401000	-3.63777800
H	-2.70185600	-0.67853300	-2.44089100
C	-6.09861100	1.16350700	-2.43956800
H	-5.88127700	1.27382200	-0.30741800
C	-5.49858200	-0.74052400	3.67176100
H	-6.97104700	-1.76329200	2.47515900
H	-3.85596600	0.33064200	4.56467900
C	-5.53711400	0.75712200	-3.65114500
H	-3.85156100	-0.22265400	-4.56849700
H	-7.04893100	1.68853700	-2.42938800
H	-6.00023000	-0.93551900	4.61388500
H	-6.04900400	0.95047600	-4.58804800
N	0.73174700	-2.47107200	0.28231700
C	1.22340900	-2.78077600	1.58813000
C	0.64622200	-3.47320100	-0.71000800
C	2.51469900	-3.29680500	1.75976300
C	0.42931700	-2.55890500	2.72126400
C	0.30189200	-3.13509600	-2.03071000

C	0.86590400	-4.83273600	-0.41826900
C	2.99761100	-3.58478600	3.03366100
H	3.13670000	-3.46753200	0.88924800
C	0.92052700	-2.83886200	3.99503200
H	-0.57440500	-2.16723400	2.60424900
C	0.20719200	-4.10992400	-3.01835700
H	0.11141900	-2.09998900	-2.28082600
C	0.77551100	-5.79908500	-1.41617100
H	1.09610700	-5.14121600	0.59303100
C	2.20609200	-3.35507700	4.16002500
H	4.00294000	-3.97912700	3.14669700
H	0.28900500	-2.66187100	4.86019100
C	0.44956900	-5.45239700	-2.72733800
H	-0.05835100	-3.81156300	-4.02834900
H	0.94977500	-6.83909000	-1.15562300
H	2.58649300	-3.57518400	5.15222500
H	0.37654300	-6.21034300	-3.50031800
N	2.19610800	-0.00220300	0.00893600
C	2.89553800	0.59218300	1.09178600
C	2.90979100	-0.60172400	-1.06097400
C	4.09746600	1.28785100	0.87848300
C	2.41043400	0.49073600	2.40240700
C	2.43938800	-0.51155800	-2.37777700
C	4.11239500	-1.29071600	-0.82933500
C	4.79268100	1.84814900	1.94552900
H	4.48862100	1.38431000	-0.12684200
C	3.10515200	1.06407600	3.46447200
H	1.49513700	-0.05496700	2.59144500
C	3.14828100	-1.08986700	-3.42761700
H	1.52458900	0.02968800	-2.58118900
C	4.82165500	-1.85601100	-1.88442600
H	4.49267300	-1.37889400	0.18086300
C	4.30334300	1.74273100	3.24877000
H	5.71859700	2.38198400	1.75307200
H	2.70834200	0.96697800	4.47041700
C	4.34663200	-1.76224100	-3.19380100
H	2.76236200	-1.00162500	-4.43860300
H	5.74754000	-2.38453600	-1.67759400
H	4.84570300	2.18364200	4.07875900

H	4.89992900	-2.20697200	-4.01447700
N	0.74749200	2.46945500	-0.29695900
C	0.66798800	3.48130000	0.68588800
C	1.24517400	2.76307900	-1.60400600
C	0.88840300	4.83747800	0.37963400
C	0.32994800	3.15627800	2.01132300
C	0.45490700	2.53111200	-2.73783700
C	2.53880900	3.27315400	-1.77638200
C	0.80475600	5.81361300	1.36850200
H	1.11404200	5.13544600	-0.63587900
C	0.24224800	4.14078100	2.98996300
H	0.14047400	2.12367500	2.27222900
C	0.95177800	2.79614400	-4.01262300
H	-0.55036400	2.14353300	-2.62039700
C	3.02738200	3.54620700	-3.05138200
H	3.15814100	3.45139600	-0.90544900
C	0.48518200	5.47998700	2.68464700
H	0.97928800	6.85078900	1.09708900
H	-0.01781900	3.85267200	4.00435700
C	2.23932600	3.30721900	-4.17826000
H	0.32301600	2.61180800	-4.87826600
H	4.03436700	3.93617400	-3.16472600
H	0.41773900	6.24541300	3.45073500
H	2.62401700	3.51583300	-5.17127900

■ **4DP-IPN (radical cation)**

Electronic energy = -2486.843827 (hartree)

Electronic energy + zero-point energy = -2486.038249 (hartree)

Electronic energy + thermal energy correction = -2485.988253 (hartree)

Electronic energy + thermal enthalpy correction = -2485.987309 (hartree)

Electronic energy + thermal free energy correction = -2486.125674 (hartree)

C	-0.04518900	1.23091800	0.08097500
C	1.36631900	1.20701900	0.11814100
C	2.09352800	0.00032300	-0.00015800
C	1.36661700	-1.20654900	-0.11837400
C	-0.04487400	-1.23082400	-0.08116300
C	-0.76138000	-0.00004300	-0.00010100
C	2.07900600	-2.43396700	-0.27179700

C	2.07844100	2.43455500	0.27184900
N	2.65729300	-3.42452200	-0.40504400
N	2.65660400	3.42513600	0.40544900
N	3.49904600	0.00048800	-0.00027900
C	4.20732800	0.55442600	1.10877800
C	4.20723100	-0.55334500	-1.10945100
C	5.36723500	1.31316600	0.90428100
C	3.74809100	0.32397400	2.41192300
C	3.74768000	-0.32306600	-2.41251800
C	5.36731800	-1.31186600	-0.90515000
C	6.05389800	1.83289900	1.99446900
H	5.71654900	1.50586700	-0.10288800
C	4.43394400	0.86389600	3.49653100
H	2.87141400	-0.29236700	2.57772100
C	4.43339800	-0.86294600	-3.49723200
H	2.87084800	0.29309100	-2.57818200
C	6.05384000	-1.83155700	-1.99544700
H	5.71687000	-1.50445000	0.10195900
C	5.58976700	1.61678700	3.29412900
H	6.94715600	2.42504600	1.82761400
H	4.07366800	0.67731300	4.50251700
C	5.58939400	-1.61562200	-3.29502400
H	4.07287000	-0.67650600	-4.50315500
H	6.94723100	-2.42354400	-1.82873800
H	6.12697700	2.03019200	4.14056600
H	6.12649000	-2.02900200	-4.14154600
N	-0.72564000	2.46814700	0.16018500
C	-1.47196200	2.76811400	1.34851600
C	-0.42450900	3.50576400	-0.77622900
C	-2.69914800	3.43732800	1.26301700
C	-0.95148600	2.44305600	2.60605200
C	-0.07611500	3.18266400	-2.09526000
C	-0.46900300	4.85539400	-0.39796600
C	-3.39528900	3.76459000	2.42212100
H	-3.09526400	3.71389400	0.29438600
C	-1.66092700	2.76337900	3.76218300
H	0.01783700	1.96419200	2.68640300
C	0.21490800	4.18684900	-3.01359700
H	-0.02690700	2.14814400	-2.40902200

C	-0.19291700	5.85176300	-1.32773900
H	-0.70275800	5.12857200	0.62263600
C	-2.88419800	3.42418600	3.67587800
H	-4.34310400	4.28659500	2.34460400
H	-1.24344300	2.51152000	4.73115900
C	0.15035700	5.52805900	-2.64077600
H	0.48379600	3.91545300	-4.02901600
H	-0.22870000	6.88985900	-1.01469900
H	-3.43047900	3.68266700	4.57636800
H	0.37134300	6.30935500	-3.35949100
N	-2.18274100	-0.00027200	0.00005800
C	-2.89243100	-0.67592900	1.02574100
C	-2.89300600	0.67507100	-1.02538300
C	-4.12486800	-1.29966900	0.75116200
C	-2.38594400	-0.69505900	2.33589600
C	-2.38700900	0.69442800	-2.33574000
C	-4.12567600	1.29821800	-0.75041300
C	-4.83097400	-1.91504400	1.77377700
H	-4.51162800	-1.31582400	-0.25856800
C	-3.10167700	-1.31686000	3.34980400
H	-1.46168100	-0.18309200	2.56544800
C	-3.10337100	1.31593000	-3.34938400
H	-1.46266700	0.18279400	-2.56568700
C	-4.83240900	1.91327900	-1.77276900
H	-4.51210000	1.31420400	0.25944800
C	-4.32657900	-1.92684800	3.07714700
H	-5.77526600	-2.39929800	1.55142700
H	-2.70872100	-1.31132300	4.36019300
C	-4.32845300	1.92535900	-3.07631300
H	-2.71076800	1.31056300	-4.35991100
H	-5.77685300	2.39708600	-1.55009200
H	-4.88486100	-2.40727900	3.87271800
H	-4.88722700	2.40554000	-3.87168800
N	-0.72491800	-2.46824500	-0.16028100
C	-0.42314500	-3.50574800	0.77610700
C	-1.47157400	-2.76843100	-1.34827100
C	-0.46664500	-4.85536800	0.39773100
C	-0.07503300	-3.18243100	2.09513600
C	-0.95208400	-2.44233100	-2.60596200

C	-2.69810600	-3.43882100	-1.26242600
C	-0.18990900	-5.85159000	1.32746600
H	-0.70017200	-5.12862000	-0.62290600
C	0.21667600	-4.18645600	3.01344200
H	-0.02664600	-2.14787500	2.40889900
C	-1.66182200	-2.76281000	-3.76185900
H	0.01675600	-1.96251600	-2.68654800
C	-3.39452800	-3.76622900	-2.42131300
H	-3.09352100	-3.71609600	-0.29371000
C	0.15307700	-5.52768600	2.64053500
H	-0.22493600	-6.88969900	1.01439000
H	0.48530300	-3.91493400	4.02889600
C	-2.88442000	-3.42481300	-3.67520000
H	-1.24508400	-2.51013400	-4.73094300
H	-4.34183000	-4.28912500	-2.34352000
H	0.37457000	-6.30886500	3.35922200
H	-3.43092500	-3.68343700	-4.57551300

■ **UVA-1 (neutral)**

Electronic energy = -820.985539 (hartree)

Electronic energy + zero-point energy = -820.750080 (hartree)

Electronic energy + thermal energy correction = -820.732934 (hartree)

Electronic energy + thermal enthalpy correction = -820.731990 (hartree)

Electronic energy + thermal free energy correction = -820.796594 (hartree)

C	2.47035300	-1.16958400	-0.00257400
C	1.20014900	-0.41541200	-0.08792000
C	1.31294600	1.06618200	-0.29792500
O	1.32730800	1.61398400	-1.37803200
O	2.57503400	-2.37084200	0.15150300
O	1.40510300	1.72210100	0.86924400
O	3.53530500	-0.34774600	-0.11965100
C	1.57742300	3.15438500	0.79625600
H	1.64660300	3.48858200	1.82787300
H	2.49054900	3.39552500	0.25267000
H	0.72264600	3.61520000	0.30163600
C	4.83635800	-0.96115400	-0.05958800
H	4.95176600	-1.68664900	-0.86471100
H	5.54567200	-0.14616500	-0.17822400

H	4.97996300	-1.45456200	0.90161500
C	0.04065500	-1.10765500	0.02252800
H	0.18605100	-2.17437800	0.17798000
C	-1.34789400	-0.69795100	-0.01296900
C	-2.32449500	-1.67019300	0.29521900
C	-1.81523900	0.59480100	-0.33607100
C	-3.67607600	-1.37904500	0.30290000
H	-2.00447100	-2.67806900	0.54171800
C	-3.16368900	0.89596000	-0.33842900
H	-1.12173400	1.37721900	-0.61541800
C	-4.12688200	-0.08313300	-0.01442800
H	-4.39642600	-2.15311100	0.54941500
H	-3.49049300	1.89710300	-0.60290400
N	-5.47079000	0.20793800	-0.06411000
H	-6.10938600	-0.41635000	0.40442300
H	-5.74886600	1.17689700	-0.03379900

■ UVA-1 (radical anion)

Electronic energy = -821.073331 (hartree)

Electronic energy + zero-point energy = -820.841382 (hartree)

Electronic energy + thermal energy correction = -820.824254 (hartree)

Electronic energy + thermal enthalpy correction = -820.823310 (hartree)

Electronic energy + thermal free energy correction = -820.887508 (hartree)

C	-2.56765500	-1.01243100	-0.07761800
C	-1.36518400	-0.22724300	0.07770600
C	-1.41754400	1.22049700	0.00174900
O	-2.17591900	1.93324800	-0.65366100
O	-2.61692200	-2.24866800	-0.07759500
O	-0.45759900	1.81403400	0.80216100
O	-3.73777100	-0.29263700	-0.16516100
C	-0.35067100	3.23356300	0.71250600
H	0.47777200	3.50907500	1.36404900
H	-0.13977000	3.55355300	-0.31075600
H	-1.26619300	3.72382300	1.05059700
C	-4.93097000	-1.05429600	-0.33899900
H	-5.11649400	-1.70953300	0.51515200
H	-5.73451100	-0.32343800	-0.42499900
H	-4.88613800	-1.66314500	-1.24488600

C	-0.18911900	-0.97110400	0.43218000
H	-0.39035600	-1.95479600	0.84908300
C	1.19122800	-0.70056300	0.19642600
C	2.17857300	-1.55872600	0.76261600
C	1.69885700	0.34530200	-0.62678500
C	3.53584500	-1.37850000	0.55415600
H	1.84939000	-2.38716900	1.38516600
C	3.05866400	0.52121000	-0.83994500
H	1.00826600	1.01106500	-1.12942500
C	4.00956900	-0.32731900	-0.25109600
H	4.24575000	-2.05951000	1.01855000
H	3.39543400	1.33100000	-1.48410300
N	5.39501300	-0.09667400	-0.40834900
H	5.95757000	-0.93455300	-0.32920300
H	5.63033300	0.39020500	-1.26410400

■ UVA-1 (radical cation)

Electronic energy = -820.770482 (hartree)

Electronic energy + zero-point energy = -820.533833 (hartree)

Electronic energy + thermal energy correction = -820.516894 (hartree)

Electronic energy + thermal enthalpy correction = -820.515950 (hartree)

Electronic energy + thermal free energy correction = -820.580581 (hartree)

C	-2.45028800	-1.18367000	-0.01295800
C	-1.16730000	-0.40652700	0.07031300
C	-1.29684300	1.07661100	0.28399500
O	-1.24046900	1.58996000	1.37709600
O	-2.50811800	-2.37677800	-0.20388100
O	-1.48868900	1.71830000	-0.86331700
O	-3.50313400	-0.38229400	0.15458300
C	-1.71308800	3.15062300	-0.78510700
H	-1.88285100	3.46638400	-1.80979000
H	-2.58572500	3.35371200	-0.16662500
H	-0.83542900	3.64349200	-0.36898200
C	-4.81064200	-1.00076800	0.10566600
H	-4.89339000	-1.75682400	0.88505400
H	-5.51491000	-0.19218100	0.27609100
H	-4.97025100	-1.45335300	-0.87206900
C	-0.00727700	-1.11562900	-0.02620200

H	-0.15583400	-2.18079900	-0.17983800
C	1.36171100	-0.69333500	0.01380700
C	2.35344900	-1.68566000	-0.26861700
C	1.80837100	0.63017100	0.31983000
C	3.68475000	-1.38983000	-0.28031500
H	2.03182400	-2.69727000	-0.48711800
C	3.13466400	0.94567800	0.31707200
H	1.09884800	1.39990200	0.58545000
C	4.11426300	-0.05417100	0.00650300
H	4.42128200	-2.15313100	-0.50343600
H	3.46323700	1.95092800	0.55442000
N	5.40467800	0.25885500	-0.00824200
H	6.11756700	-0.42375500	-0.23006700
H	5.72553900	1.19644300	0.19484000

■ UVA-2 (neutral)

Electronic energy = -900.358628 (hartree)

Electronic energy + zero-point energy = -900.075359 (hartree)

Electronic energy + thermal energy correction = -900.056488 (hartree)

Electronic energy + thermal enthalpy correction = -900.055544 (hartree)

Electronic energy + thermal free energy correction = -900.124790 (hartree)

C	2.02319600	-0.22300300	-0.46081000
O	2.18816900	0.71011100	-1.21267400
O	3.01743700	-0.89827100	0.13074400
C	4.37815300	-0.48946600	-0.18741100
H	4.51158000	-0.56363700	-1.26812300
H	4.49642500	0.55674500	0.10006000
C	-0.47239000	-0.08561000	-0.05385400
C	-0.46436000	1.39650000	-0.01803300
C	-1.31985200	2.12669000	-0.85704400
C	0.35098600	2.08955200	0.88882400
C	-1.32607400	3.51783100	-0.81919500
H	-1.96505200	1.60315400	-1.55390000
C	0.32381400	3.47948200	0.94330000
H	0.98992900	1.53666300	1.56919900
H	-1.97552300	4.07110300	-1.48930900
H	0.94929400	4.00207200	1.65932700
C	0.68606800	-0.81436500	-0.10850500

C	0.69452700	-2.21888600	0.14845200
N	0.74404100	-3.35423600	0.36729000
C	5.31897700	-1.40054200	0.56930800
H	5.16656100	-1.32006600	1.64785500
H	5.18188900	-2.44343400	0.27575900
H	6.35181500	-1.11797100	0.34998700
C	-0.50705200	4.19771600	0.08281700
H	-0.52231700	5.28194300	0.12079400
C	-1.79890900	-0.74119800	0.02392800
C	-2.76275800	-0.27489400	0.93409000
C	-2.13163000	-1.80851000	-0.82565300
C	-4.01051900	-0.88409000	1.01604400
H	-2.52459800	0.55518100	1.58954800
C	-3.38969500	-2.39951300	-0.75839900
H	-1.41489200	-2.15776100	-1.55978900
C	-4.32865000	-1.94564100	0.16779200
H	-4.73741300	-0.52648000	1.73754900
H	-3.63719700	-3.21282800	-1.43215900
H	-5.30664100	-2.41206800	0.22370800

■ UVA-2 (radical anion)

Electronic energy = -900.470483 (hartree)

Electronic energy + zero-point energy = -900.189751 (hartree)

Electronic energy + thermal energy correction = -900.171051 (hartree)

Electronic energy + thermal enthalpy correction = -900.170107 (hartree)

Electronic energy + thermal free energy correction = -900.238500 (hartree)

C	1.96855000	-0.12156500	-0.39535200
O	2.03841800	0.78831100	-1.21917500
O	3.10808800	-0.78080000	0.02033900
C	4.34131300	-0.40494800	-0.61263000
H	4.24812500	-0.53640500	-1.69434900
H	4.53791600	0.65429500	-0.42516700
C	-0.52616500	-0.01668600	0.07103900
C	-0.64838400	1.44023900	0.09509300
C	-1.62853100	2.12339000	-0.66320200
C	0.21896700	2.23705000	0.87815100
C	-1.73614200	3.50941000	-0.63534100
H	-2.29598700	1.55305300	-1.30006500

C	0.10645900	3.62147800	0.91009800
H	0.97658800	1.74690700	1.48002700
H	-2.49349600	3.99895600	-1.24132900
H	0.78330100	4.19878200	1.53386100
C	0.77537700	-0.63902000	0.22762800
C	0.89596200	-1.80624300	1.00898200
N	0.97104100	-2.76542100	1.66952000
C	5.44080900	-1.27982200	-0.04161200
H	5.53168500	-1.14457500	1.03916400
H	5.24700300	-2.33715000	-0.23863400
H	6.39935700	-1.01804300	-0.49912400
C	-0.87218000	4.27382200	0.15392100
H	-0.95702600	5.35580100	0.17645000
C	-1.71753100	-0.85278800	-0.05136100
C	-2.97502200	-0.45972400	0.47123400
C	-1.66230200	-2.11917000	-0.68410600
C	-4.09703600	-1.27194400	0.35939000
H	-3.05838600	0.48862900	0.99019100
C	-2.78736500	-2.92683200	-0.79900800
H	-0.72126300	-2.45153000	-1.10778000
C	-4.01758900	-2.51311800	-0.27994200
H	-5.04064800	-0.94017800	0.78358500
H	-2.70682300	-3.88546900	-1.30364100
H	-4.89484100	-3.14639200	-0.36702900

■ UVA-2 (radical cation)

Electronic energy = -900.107562 (hartree)

Electronic energy + zero-point energy = -899.824813 (hartree)

Electronic energy + thermal energy correction = -899.805808 (hartree)

Electronic energy + thermal enthalpy correction = -899.804864 (hartree)

Electronic energy + thermal free energy correction = -899.874622 (hartree)

C	1.95945500	-0.47315800	-0.58096000
O	2.05895400	0.19863400	-1.57887400
O	2.93834500	-0.94919500	0.16356300
C	4.31724300	-0.67108600	-0.26965700
H	4.43252900	-1.08901000	-1.27012000
H	4.43436000	0.41143000	-0.32660200
C	-0.52157700	-0.07420300	-0.02655900

C	-0.34564100	1.36450600	0.00875100
C	-1.28481800	2.20009900	-0.65426100
C	0.72627700	1.96443300	0.71781800
C	-1.14072300	3.57584800	-0.62235300
H	-2.07818800	1.75181700	-1.23907900
C	0.84041000	3.33886200	0.77130300
H	1.42488400	1.34799600	1.27137300
H	-1.83921100	4.20611500	-1.15961800
H	1.64327100	3.79409800	1.33877100
C	0.60370300	-0.93408300	-0.08398600
C	0.51360300	-2.30119700	0.24339500
N	0.49897500	-3.42515500	0.53285700
C	5.24541200	-1.30638500	0.73736200
H	5.10144100	-0.88365900	1.73360000
H	5.09664100	-2.38658500	0.78801700
H	6.27873100	-1.11942500	0.43555700
C	-0.08568200	4.14823000	0.09396200
H	0.02400600	5.22651600	0.12257100
C	-1.86759700	-0.61474500	0.03143400
C	-2.84156000	0.02083500	0.84987300
C	-2.24585900	-1.75161700	-0.72820600
C	-4.12923300	-0.47925600	0.91983400
H	-2.55228600	0.86044200	1.46962700
C	-3.54349400	-2.22126600	-0.67502200
H	-1.53685500	-2.21910600	-1.39968300
C	-4.48530100	-1.59467300	0.15517900
H	-4.85623200	-0.01298100	1.57390600
H	-3.83552500	-3.07137800	-1.27991900
H	-5.49649300	-1.98256900	0.20663800

Supplementary References

1. Singh, V. K. et al. Highly efficient organic photocatalysts discovered via a computer-aided-design strategy for visible-light-driven atom transfer radical polymerization. *Nat. Catal.* **1**, 794–804 **(**2018**).**
2. Kwon, Y. et al. Formation and degradation of strongly reducing cyanoarene-based radical anions towards efficient radical anion-mediated photoredox catalysis. *Nat. Commun.* **14**, 92 (2023).
3. Back, J. et al. Visible light curable acrylic resins toward UV-light blocking adhesives for foldable displays. *Adv. Mater.* **35**, 2204776 (2023).
4. Roth, H. G., Romero, N. A. & Nicewicz, D. A. Experimental and calculated electrochemical potentials of common organic molecules for applications to single-electron redox chemistry. *Synlett* **27**, 714–723 (2016).
5. Yang, H. et al. Multi-layer in-situ for evaluation of dynamic mechanical properties of pressure sensitive adhesives. *Int. J. Adhes. Adhes.* **27**, 536–546 (2007).
6. Abrahamson, T. et al. J. Optically clear adhesives for OLED. *Lumin.* **-** *OLED Technol. Appl.* (2020).
7. Furukawa, T., Nakanotani, H., Inoue, M. & Adachi, C. Dual enhancement of electroluminescence efficiency and operational stability by rapid upconversion of triplet excitons in OLEDs. *Sci. Rep.* **5**, 8429 (2015).
8. Kikuchi, A., Nakabai, Y., Oguchi-Fujiyama, N., Miyazawa, K. & Yagi, M. Energy-donor phosphorescence quenching study of triplet-triplet energy transfer between UV absorbers. *Journal of Luminescence* **166**, 203–208 (2015).
9. Würth, C., et al. Comparison of methods and achievable uncertainties for the relative and absolute measurement of photoluminescence quantum yields. *Anal. Chem.* **83**, 3431–3439 (2011).
10. Baleizão, C. & Berberan-Santos, M. N. Thermally activated delayed fluorescence as a cycling process between excited singlet and triplet states: Application to the fullerenes. *J. Chem. Phys.* **126**, 204510 (2007).
11. Patil, V. V., et al. Purely spin-vibronic coupling assisted triplet to singlet up-conversion for real deep blue organic light-emitting diodes with over 20% efficiency and y color coordinate of 0.05. *Adv. Sci.* **8**, 2101137 (2021).
12. Konidena, R. K., Lim, J., Lee, J. Y. A novel molecular design featuring the conversion of inefficient TADF emitters into efficient TADF emitters for deep-blue organic light emitting diodes. *J. Chem. Eng.* **416**, 129097 (2021).
13. Gierschner, J. *et al.* Luminescence in crystalline organic Materials: from molecules to molecular solids. *Adv. Opt. Mater.* **9**, 1–46 (2021).
14. Shi, J. *et al.* Solid state luminescence enhancement in π-conjugated materials: unraveling the mechanism beyond the framework of AIE/AIEE. *J. Phys. Chem. C* **121**, 23166–23183 (2017).
15. Goushi, K., Yoshida, K., Sato, K., Adachi, C. Organic light-emitting diodes employing efficient reverse intersystem crossing for triplet-to-singlet state conversion. *Nat. Photonics* **6**, 253–258 (2012).
16. Tanimoto, S., Suzuki, T., Nakanotani, H., Adachi, C. Thermally activated delayed fluorescence from pentacarbazorylbenzonitrile. *Chem. Lett.* **45**, 770–772 (2016).
17. D'Andrade, B. W. et al. Relationship between the ionization and oxidation potentials of molecular organic semiconductors. *Organic Electronics* **6**, 11–20 (2005).
18. Romero, N. A. & Nicewicz, D. A. Organic photoredox catalysis. *Chem Rev*. **116**, 10075–10166 (2016).
19. Chatterjee, S. et al. Photochemistry of carbocyanine alkyltriphenylborate salts: intra-ion-pair electron transfer and the chemistry of boranyl radicals. *J. Am. Chem. Soc.* **112**, 6329–6338 (1990).
20. Maafi, M. & Brown, R. G. The kinetic model for AB(1φ{symbol}) systems. A closed-form integration of the differential equation with a variable photokinetic factor. *J. Photochem. Photobiol. A Chem.* **187**, 319–324 (2007)
21. Watts, D. C. Reaction kinetics and mechanics in photo-polymerised networks. *Dent. Mater.* **21**, 27–35 (2005).
22. Tsuchiya, Y. et al. Exact solution of kinetic analysis for thermally activated delayed fluorescence materials. *J. Phys. Chem. A* **125**, 8074–8089 (2021).
23. Costentin, C., Robert, M. & Savéant, J. M. Electron transfer and bond breaking: recent advances. *Chem. Phys.* **324**, 40–56

(2006).

24. Koyama, D., Dale, H. J. A. & Orr-Ewing, A. J. Ultrafast observation of a photoredox reaction mechanism: photoinitiation in organocatalyzed atom-transfer radical polymerization. *J. Am. Chem. Soc.* **140**, 1285–1293 (2018).
25. Romańczyk, P. P. & Kurek, S. S. Reliable reduction potentials of diaryliodonium cations and aryl radicals in acetonitrile from high-level ab initio computations. *Electrochim. Acta* **351**, 136404 (2020).
26. Pause, L., Robert, M. & Savéant, J. M. Stepwise and concerted pathways in photoinduced and thermal electron-transfer/bond-breaking reactions. Experimental illustration of similarities and contrasts. *J. Am. Chem. Soc.* **123**, 4886–4895 (2001).
27. Pan, X. et al. Mechanism of photoinduced metal-free atom transfer radical polymerization: experimental and computational studies. *J. Am. Chem. Soc.* **138**, 2411–2425 (2016).
28. Hu, J., Wang, J., Nguyen, T. H. & Zheng, N. The chemistry of amine radical cations produced by visible light photoredox catalysis. *Beilstein J. Org. Chem.* **9**, 1977–2001 (2013).
29. Yu, C. et al. Defect-free acrylic polymers with a near-poisson distribution prepared via catalyst-free visible-light-driven radical polymerization. *ChemRxiv* doi:10.26434/chemrxiv-2023-dl842 (2023).
